# BisBINOL-Based Fluorescent Probes: Effect of Alkane Linkers and Chemo- and Enantioselective Recognition of Arginine

**DOI:** 10.1021/acs.joc.5c01699

**Published:** 2025-11-05

**Authors:** Yichen Li, Carson Patrick, Franklin He, Sydney Rohrbach, Yifan Mao, Lin Pu

**Affiliations:** Department of Chemistry, 2358 University of Virginia, Charlottesville, Virginia 22904, United States

## Abstract

A series of bisBINOL-based fluorescent probes, which contain two BINOL units linked by an alkane chain of varying length, were synthesized and characterized. It was found that the chain length of the alkane linker has strong effect on the fluorescence response of these compounds toward amino acids. Among them, compound (*S*,*S*)-**6** that contains a six-carbon alkyl chain linker exhibits highly chemoselective and enantioselective fluorescence enhancement with arginine in the presence of Zn^2+^, but other compounds show much less selective fluorescence response. Spectroscopic studies have been conducted to investigate the mechanism of this process.

## Graphical abstract

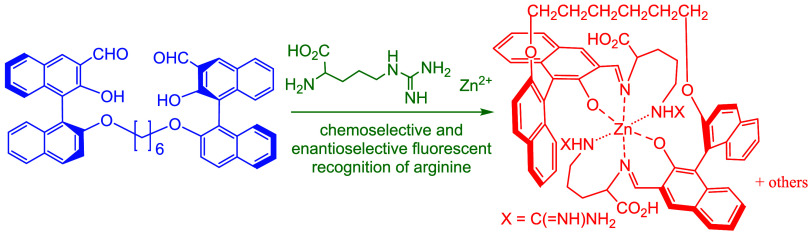

## 1 Introduction

Development of chemoselective as well as enantioselective fluorescent probes for amino acids has received significant attention in recent years because of the importance of amino acids in both nature and organic synthesis and also the advantages of the fluorescence-based detection such as easily available instrument, high sensitivity and potential for online and remote sensing.^1,2^ In this research area, the use of the 1,1′-bi-2-naphthol­(BINOL)-based compounds as fluorescent probes for selective recognition of amino acids has been actively investigated.^3^ Among these studies, a few bisBINOL-based compounds have shown excellent selectivity.^4^ For example, compound (*S*,*S*)-**1** containing two BINOL units linked with a pyridinyl unit has exhibited highly enantioselective fluorescent responses toward a variety of chiral amino acids (Chart 1).^4a^

**Chart 1:**
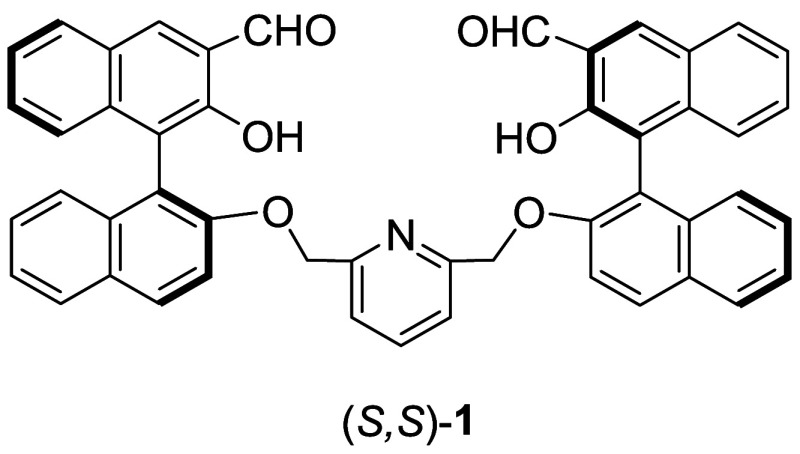
A bisBINOL-Based Enantioselective Fluorescent Probe for Amino Acids

It has been surmised that connecting two BINOL units could potentially increase the chiral bias of an individual one and enhance the enantioselective response. However, no systematic study has been conducted on the effect of the linker length of the bisBINOL probes in fluorescent recognition. We have thus designed a series of optically active bisBINOL-based compounds that contain simple alkyl linkers of various chain lengths. These compounds could react with amino acids in the presence of Zn^2+^ to generate Zn­(II) complexes with a possible structure as shown in Chart 2.^5^ It is proposed that if the R group in the amino acid units of this structure has additional functions to coordination with the Zn­(II) center, such as the guanidine group in arginine, the resulting complex could exhibit very different structural and optical properties from those generated from other amino acids, leading to selective recognition of such an amino acid. Through variation of the length of the alkyl linker, that is the number *n*, we might be able to systematically tune the selectivity and sensitivity of this probe in the fluorescent recognition of chiral amino acids. Our work on these bisBINOL compounds has revealed dramatic effects of these linkers on their fluorescence responses toward amino acids. This study has led to the discovery of a highly chemoselective and enantioselective fluorescent probe for arginine, an amino acid of great biological significance. Herein, these results are reported.

**Chart 2:**
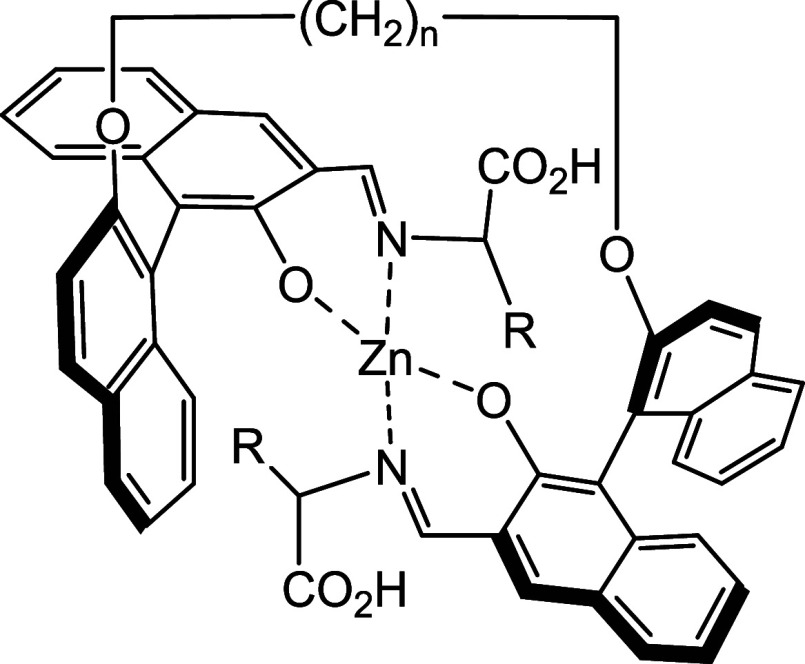
A Proposed bisBINOL-Amino Acid-Zn­(II) Complex

## 2 Results and Discussion

### 2.1 Synthesis and Spectroscopic Properties of a Series of BisBINOL Compounds with Alkyl Linkers

(*S*)-BINOL [(*S*)-**2**] was used to prepare the mono-MOM-protected BINOL (*S*)-**3** which was then converted to compound (*S*)-**4** by following a previously reported procedure (Scheme 1).^4a,6a^ Treatment of (*S*)-**4** with 1,n-dibromoalkanes in the presence of base followed by acidic hydrolysis to remove the MOM groups gave the bisBINOL compounds (*S*,*S*)-**5**,^6^ (*S*,*S*)-**6**, (*S*,*S*)-**7** and (*S*,*S*)-**8** that contain 4, 6, 8, and 10 carbon chain linkers, respectively. ^1^H NMR spectra of these compounds give similar signals for their hydroxyl, aldehyde and aromatic protons. For example, a singlet at δ 10.36 is observed for the hydroxyl proton of (*S*,*S*)-**5** which indicates strong intramolecular hydrogen bonding between the hydroxyl proton and the carbonyl group in these compounds. The UV spectra of these compounds in DMSO solution show similar absorptions (Figure 1a). For example, compound (*S*,*S*)-**6** displays absorptions at λ­(ε) = 296 (2.87 × 10^4^), 308 (2.30 × 10^4^), 340 (6.30 × 10^3^), and 400 (6.20 × 10^3^) nm. The fluorescence spectra of these compounds are also similar with λ_em_ = 480 nm while excited at 405 nm (Figure 1b).

**Figure 1:**
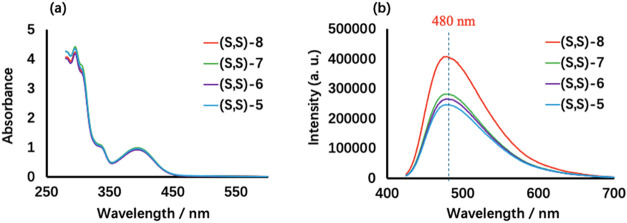
UV (a) and Fluorescence (b) Spectra of the BisBINOL Compounds (*S*,*S*)-**5**, (*S*,*S*)-**6**, (*S*,*S*)-**7** and (*S*,*S*)-**8** in DMSO (Concentration: 0.31 mM, λ_exc_ = 405 nm. Slit: 5/5 nm).

**Scheme 1:**
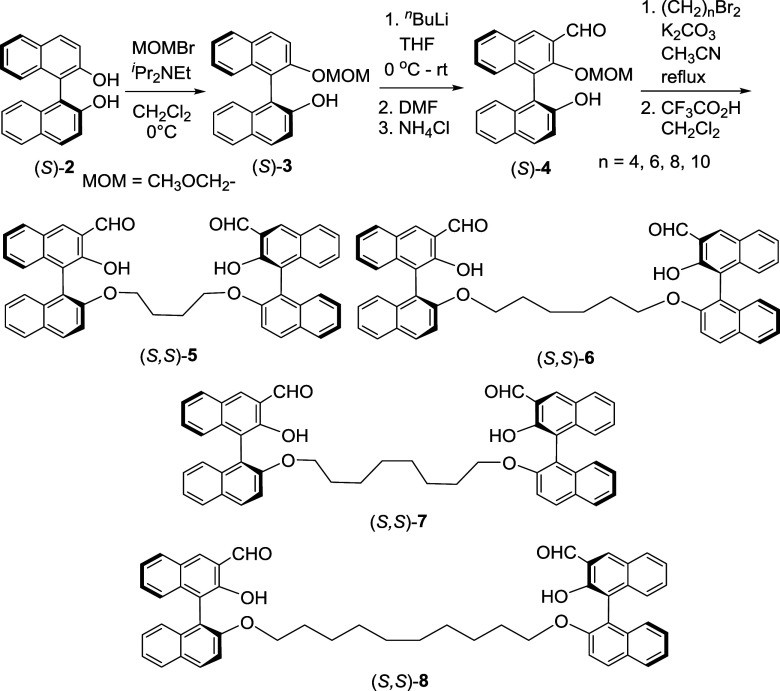
Synthesis of the BisBINOL Compounds (*S*,*S*)-**5**, (*S*,*S*)-**6**, (*S*,*S*)-**7**, and (*S*,*S*)-**8**

### 2.2. Fluorescence Responses of the BisBINOL Compounds toward Amino Acids

We investigated the fluorescence response of the bisBINOL compounds (*S*,*S*)-**5** – (*S*,*S*)-**8** toward amino acids in the presence of Zn­(OAc)_2_ (Figure 2). The samples were prepared by mixing a solution of a bisBINOL compound in DMSO with Zn­(OAc)_2_ (1.0 equiv) in water and an amino acid in phosphate buffer (pH = 7.0, 10 equiv) for 4 h before measurement. The final concentration of each sample was 0.25 mM in 1:1.5 DMSO/H_2_O. As shown in Figure 2b, when (*S*,*S*)-**6** was treated with various amino acids in the presence of Zn­(OAc)_2_ (1.0 equiv), only l-Arg greatly enhanced the fluorescence at λ = 550 nm. d-Arg as well as other amino acids and their enantiomers caused little fluorescence enhancement. The bisBINOL compounds (*S*,*S*)-**7** and (*S*,*S*)-**8** that contain a longer alkyl linker show low fluorescence responses under these conditions (Figure 2c,d). Compound (*S*,*S*)-**5** that contains a shorter alkyl linker shows lower selectivity than (*S*,*S*)-**6** toward l-Arg (Figure 2a). Thus, among the four bisBINOL compounds, (*S*,*S*)-**6** that contains a 6-carbon chain linker performs the best in the chemoselective and enantioselective fluorescent recognition of l-Arg.

**Figure 2:**
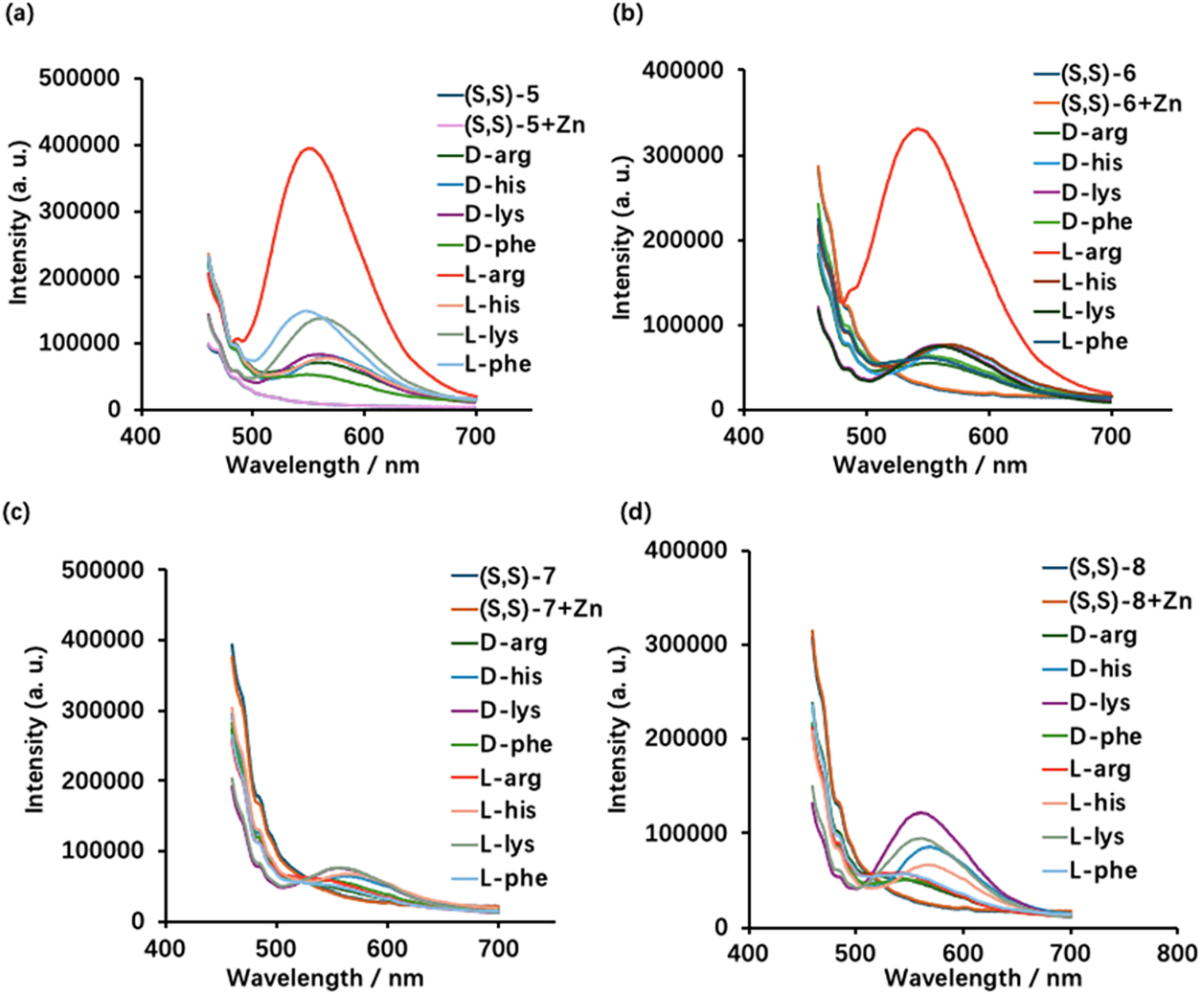
Fluorescence spectra of (a) (*S*,*S*)-**5**, (b) (*S*,*S*)-**6**, (c) (*S*,*S*)-**7**, and (d) (*S*,*S*)-**8** with various amino acids (2.5 mM, 10.0 equiv) and Zn­(OAc)_2_ (1.0 equiv) (Conc: 0.25 mM. Solvent: DMSO/60% pH 7.0 phosphate buffer. λ_exc_ = 440 nm. Slit: 5/5 nm.).

Figure 3 shows the fluorescence spectra of (*S*,*S*)-**6** (0.25 mM) with 18 pairs of amino acid enantiomers (10.0 equiv) and Zn­(OAc)_2_ (1.0 equiv) in DMSO and 60% pH = 7.0 phosphate buffer while excited at either 405 or 440 nm. The excitation at 440 nm gives higher fluorescence intensity than that at 405 nm for the emission of the probe at λ = 550 nm when treated with l-Arg. While excited at 405 nm, the fluorescence enhancement of (*S*,*S*)-**6** in the presence l-Arg is *I*/*I*_0_ = 7.4 (*I*_0_: the fluorescence intensity of the probe in the absence of amino acids). The enantioselective fluorescence enhancement ratio ef [ef = (*I*_L_ – *I*_0_)/(*I*_D_ – *I*_0_)] is 10.3. When excited at 440 nm, the fluorescence enhancement of (*S,S*)-**6** by l-Arg is *I*/*I*_0_ = 12.0 with ef = 10.8. The limit of detection is 3.9 μM (See Figure S55 in SI).

**Figure 3:**
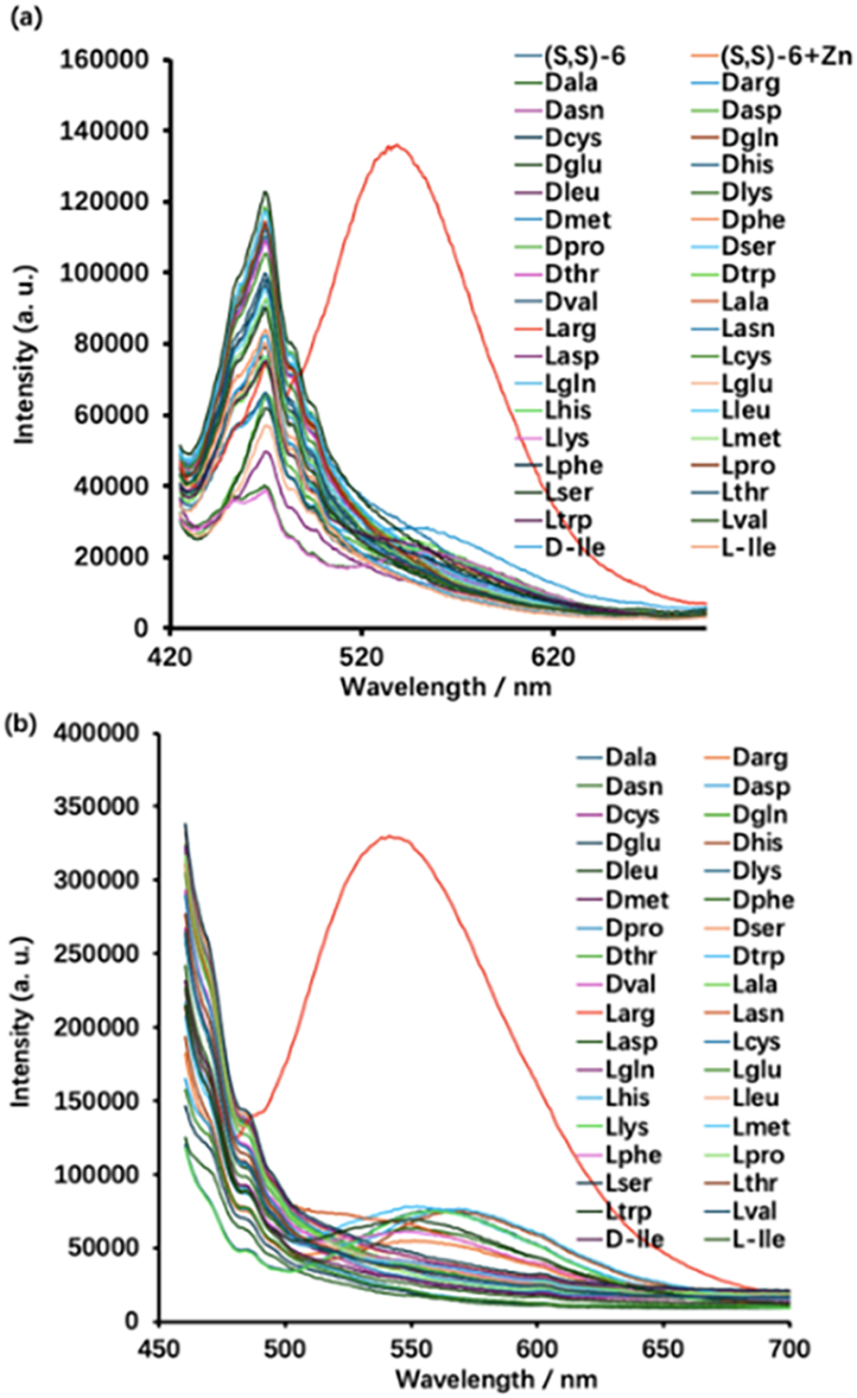
Fluorescence spectra of (*S*,*S*)-**6** (0.25 mM) + Zn­(OAc)_2_ (1.0 equiv) with 18 pairs of amino acid enantiomers (10 equiv) while excited at (a) λ_exc_ = 405 nm and (b) λ_exc_ = 440 nm (Slit: 5/5 nm. Solvent: DMSO/60% pH 7.0 phosphate buffer).

The effects of the reaction time and concentration range of l- and d-Arg on the fluorescence response of (*S,S*)-**6** were studied. As shown in Figure 4a,b, the fluorescence response of (*S,S*)-**6** toward l- and d-Arg became stable after 4 h. Figure 4c,d shows that the fluorescence of (*S*,*S*)-**6** at λ = 550 nm increases until ∼50 equiv l-Arg at λ_exc_ = 405 nm and until ∼40 equiv l-Arg at λ_exc_ = 440 nm. Thus, (*S*,*S*)-**6** can be used to analyze l-Arg up to 40–50 equiv (up to 10–12.5 mM).

**Figure 4:**
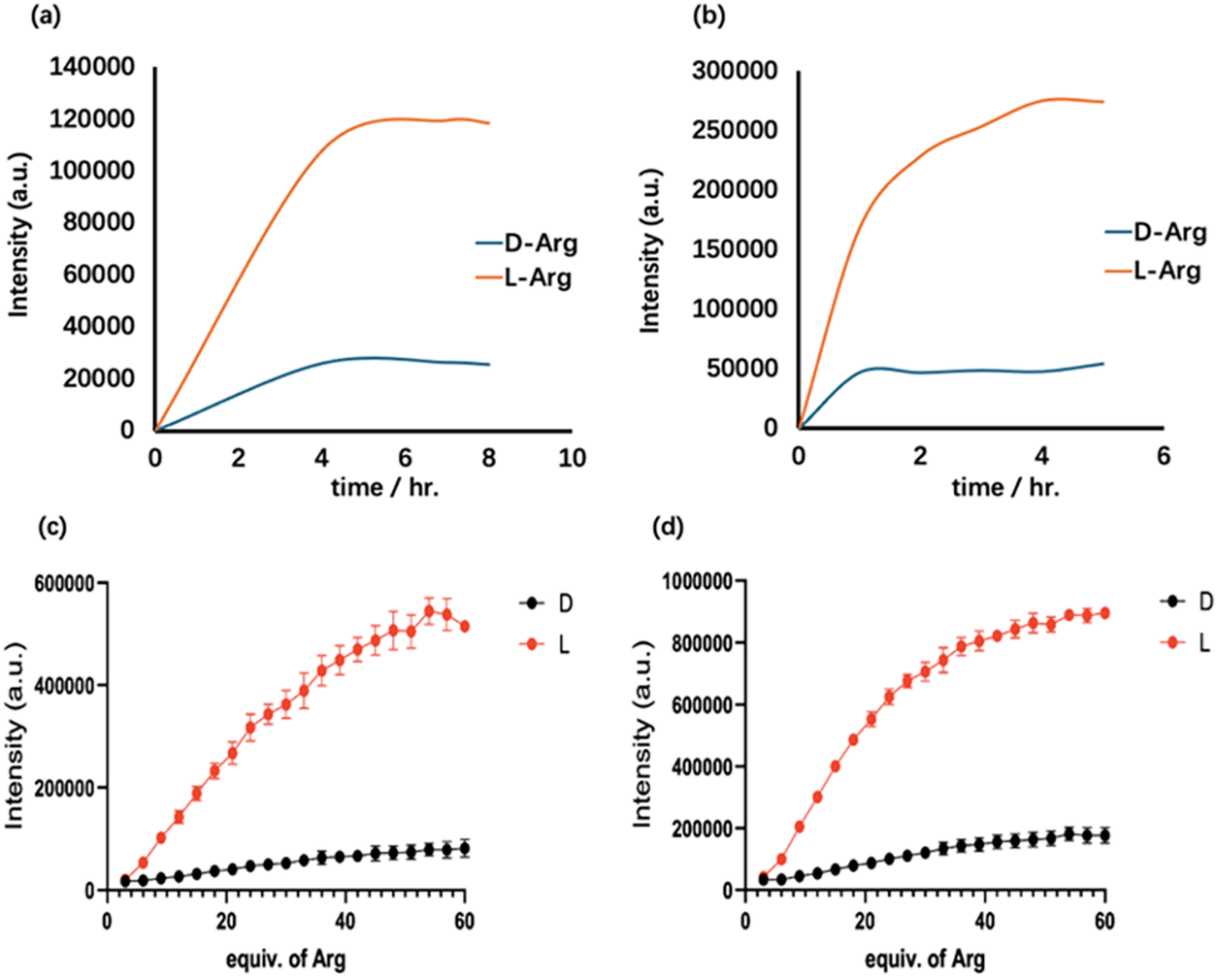
Fluorescence intensities (λ_em_ = 550 nm) of (*S,S*)-**6**, Zn­(OAc)_2_ (1.0 equiv), and d- or l-Arg (10.0 equiv) versus the reaction time (a, b), and those of (*S,S*)-**6** and Zn­(OAc)_2_ versus the concentration of d- or l-Arg after 4 h (c, d). [λ_exc_ = 405 nm for (a, c). λ_exc_ = 440 nm for (b, d). (*S*,*S*)-**6**: 0.25 mM. Solvent: DMSO/60% pH 7.0 phosphate buffer. slit = 5/5 nm. Error bars are SEM, *n* = 3].

The enantiomer of (*S*,*S*)-**6**, compound (*R*,*R*)-**6**, was prepared by starting with (*R*)-BINOL. We studied the fluorescence response of both (*S*,*S*)-**6** and (*R*,*R*)-**6** toward arginine at various ee’s [ee = enantiomeric excess = (l – d)/(l + d)]. As shown in Figure 5a–d, the fluorescence responses of (*S*,*S*)-**6** while excited at either λ_exc_ = 405 or 440 nm have a mirror image-like relationship with those of (*R*,*R*)-**6**. This confirms that the observed different fluorescence enhancement in the presence of the arginine enantiomers is due to inherent chiral recognition. Figure 5c,d demonstrate that at a given concentration, the enantiomeric composition of arginine can be determined by using the fluorescent probe. Figure 5e displays the competitive experiment results for the use of (*S*,*S*)-**6** to detect l-Arg in the presence of a smaller amount of other common amino acids and their enantiomers. It shows that under these conditions the presence of most of other amino acids had little effect on the fluorescence response of the probe toward l-Arg.

**Figure 5:**
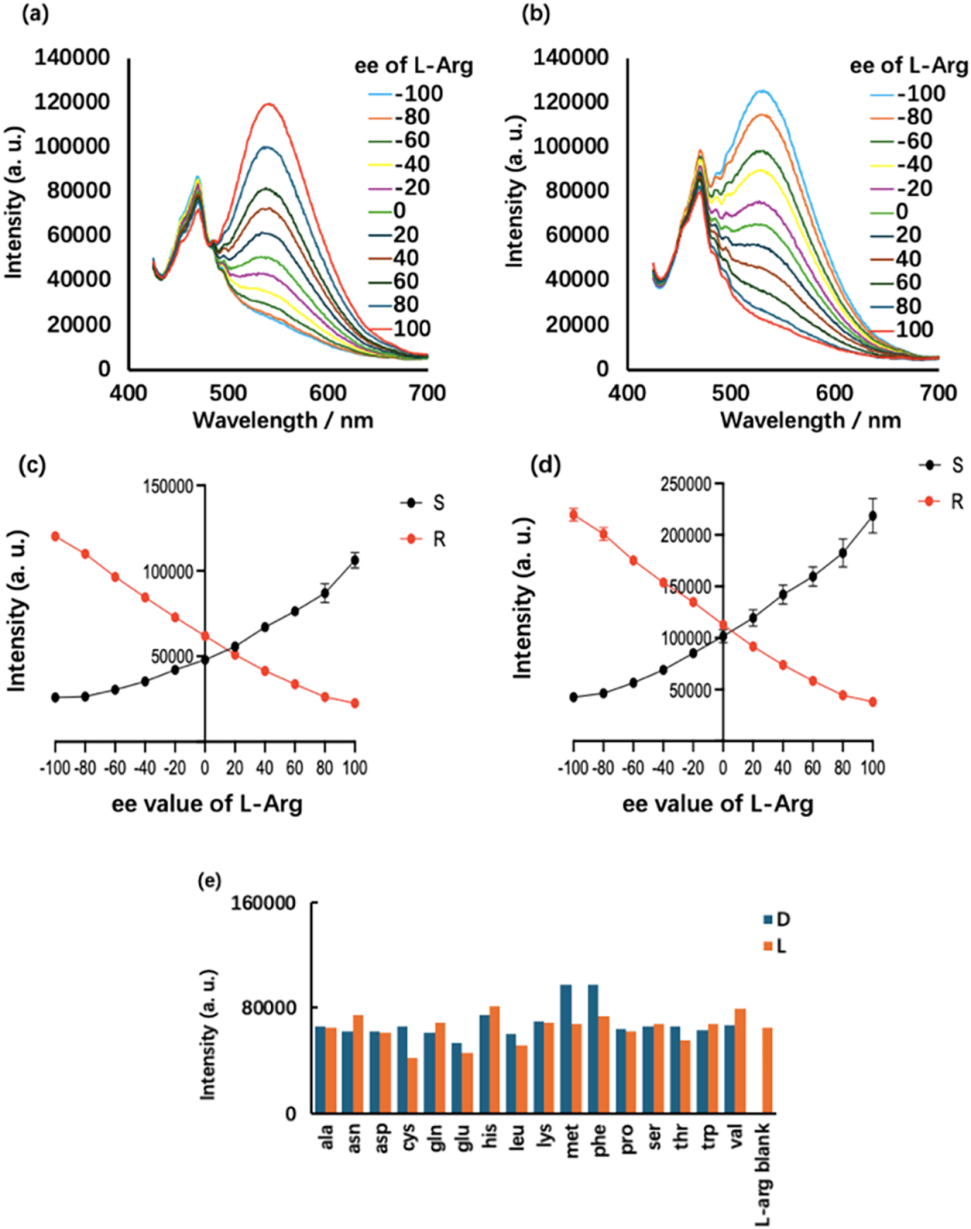
Fluorescence spectra of (a) (*S*,*S*)-**6** and (b) (*R*,*R*)-**6** with arginine at various ee’s (10 equiv) and Zn­(OAc)_2_ (1.0 equiv). Fluorescence intensity of (*S*,*S*)-**6** and (*R*,*R*)-**6** at λ_em_ = 550 nm versus the ee’s of arginine (c) λ_exc_ = 405 nm (d) λ_exc_ = 440 nm. (e) (*S*,*S*)-**6** (0.25 mM) + Zn­(OAc)_2_ (1.0 equiv) with l-Arg (3.0 equiv) + one of 16 common amino acids and their enantiomers (1.0 equiv) at 550 nm (λ_exc_ = 440 nm). (Conc: 0.25 mM. Solvent: DMSO/60% pH 7.0 phosphate buffer. Slit: 5/5 nm. Error bars are SEM, *n* = 3).

l-Arg is a semiessential amino acid involved in many biological processes such as protein biosynthesis, immune response, nitric oxide generation, cardiovascular system, nervous system, endothelial function, and urea cycle, etc.^7–11^ The enantiomer d-Arg has also been found to have interesting physiological and pharmacological functions.^12–18^ d-Arg has been identified only in the whole blood of patients with lung cancer which can be used for early cancer diagnosis.^19–21^ However, only a few reports have appeared for the enantioselective fluorescent analysis of arginine.^22^ Thus, the bisBINOL compounds (*S*,*S*)-**6** and (*R*,*R*)-**6** described here provides a new highly chemoselective as well as enantioselective fluorescent probe for arginine.

### 2.3. Study of the Interaction of (*S*,*S*)-6 with l-Arg

In order to gain a better understanding on the origin of the enantioselective fluorescent response of (*S*,*S*)-**6** toward arginine, we have conducted a NMR spectroscopic study on this reaction. Figure 6a gives the ^1^H NMR spectrum of (*S*,*S*)-**6** with l-Arg (2.0 equiv) in DMSO-*d*_6_ containing 17%D_2_O (reaction time 6 h) which shows complete reaction to form a new product. The gHSQC and gNOESY 2D NMR spectra of the new product were obtained, which supports the formation of compound **9**_L_ (Chart 3). The gHSQC spectrum (See Figure S28 in SI) gives a cross peak at δ 8.71/164.96 for the imine proton and carbon signals. In the gNOESY spectrum (See Figure S29 in SI), a cross peak at δ 8.71/3.89 between the imine proton and the α-proton of the arginine unit supports the condensation of the aldehyde group of (*S*,*S*)-**6** with the α-amine group of l-Arg rather than the guanidine group. This indicates that the more basic guanidine group of l-Arg may be in the protonated form under the reaction conditions which enables the unprotonated α-amine group to be more reactive with the aldehyde group of (*S*,*S*)-**6**. The high-resolution mass spectrum (TOF, ES+) of **9**_L_ (See Figure S30 in SI) gives an intense signal at *m*/*z* = 1023.4811 for **9**_L_+H (Calcd for C_60_H_63_N_8_O_8_: 1023.4769). A similar product, **9**_D_ (Chart 3), was obtained from the reaction of (*S,S*)-**6** with d-Arg whose NMR data support the formation of the same diimine structure (See Figure S20–S25 in SI). The high-resolution mass spectrum (TOF, ES-) of **9**_D_ (See Figure S25 in SI) gave an intense signal at *m*/*z* = 1021.4610 for **9**_D_-H (Calcd for C_60_H_61_N_8_O_8_: 1021.4612).

**Figure 6:**
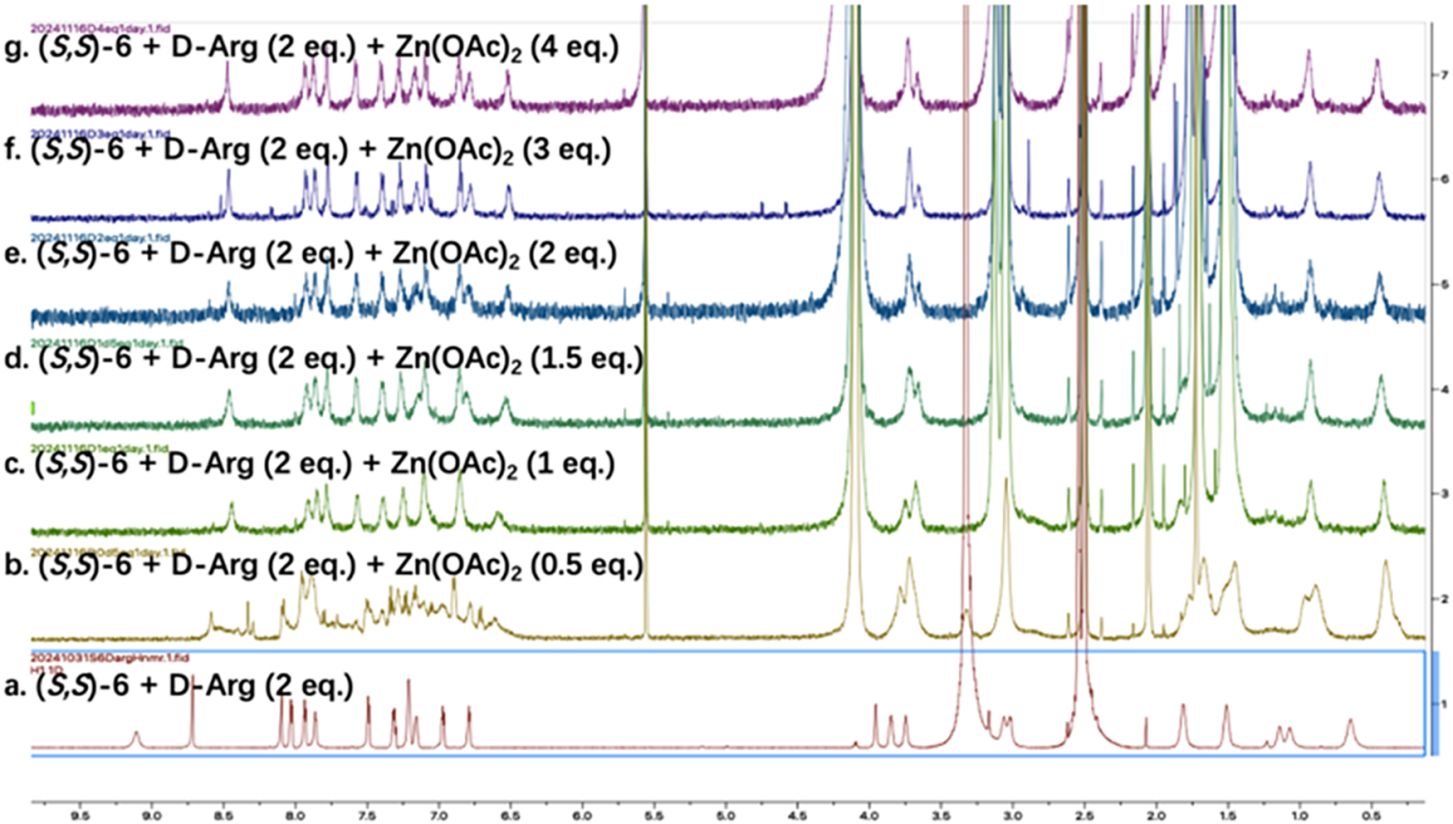
^1^H NMR spectra of (a) (*S,S*)-**6**+d-Arg and (b-g) the subsequent reaction with Zn­(OAc)_2_ (0.5 – 4 equiv) in DMSO-*d*_6_ (17% D_2_O) (See the full spectra in Figure S50 in SI).

**Chart 3:**
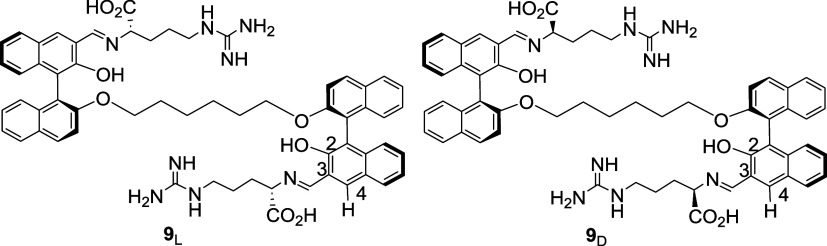
Structures of Compounds **9**_L_ and **9**_D_

We studied the reaction of the imine compound **9**_D_ with various equivalencies of Zn­(OAc)_2_ by observing changes in the ^1^H NMR spectra (Figure 6). Figure 6a is the ^1^H NMR spectrum of **9**_D_ generated from the reaction of (*S,S*)-**6** with d-Arg (2 equiv). When 1.0 equiv Zn­(OAc)_2_ was added, **9**_D_ was completely converted to a new product as shown in Figure 6c. No further significant change was observed when excess amount of Zn­(OAc)_2_ (up to 4.0 equiv) was added. This indicates that compound **9**_D_ reacts with 1.0 equiv Zn­(OAc)_2_ to form a stable complex which does not strongly coordinate with additional Zn^2+^.

Chart 4 shows a proposed structure **10** for this zinc complex.^5^ The *C*_2_ symmetric structure of **10** is consistent with the pattern of the observed ^1^H NMR signals. The gHSQC spectrum (See Figure S33b in SI) gives a cross peak at δ 8.52/168.66 for the imine proton and carbon signals. A cross peak at δ 3.15/53.47 (See Figure S33­(a) in SI) is observed for the α-proton and α-carbon of the arginine unit. According to the gTOCSY spectrum (See Figure S35 in SI), the peak at δ 3.75 could be assigned to the NH proton 21 because of its correlation with protons 16, 17, 18, and 19. The gHSQC spectrum (Figure S33a) indicates that this proton (δ 3.75) is not bonded to a carbon. In the gNOESY spectrum (See Figure S34 in SI), this signal (δ 3.72) is correlated with a few of the protons at 16, 17, 18, and 19 positions. In addition, this NH proton (21) also displays an intense NOE effect with the imine proton at δ 8.44 (See Figure S34 in SI). These observations suggest that in **10**, the guanidine group might be coordinated with the Zn^2+^ center to form a cyclic structure which brings the NH proton (21) close to the imine proton and the other protons on the arginine unit. In the high resolution mass spectrum (ESI+) for the reaction mixture of **9**_D_ + Zn­(OAc)_2_, a strong signal at *m*/*z* = 1085.39 was found for **9**_D_+Zn–H (calcd for C_60_H_61_N_8_O_8_Zn: 1085.39) (See Figure S36 in SI). At this stage, we are not able to obtain single crystals of this complex for X-ray analysis.

**Chart 4:**
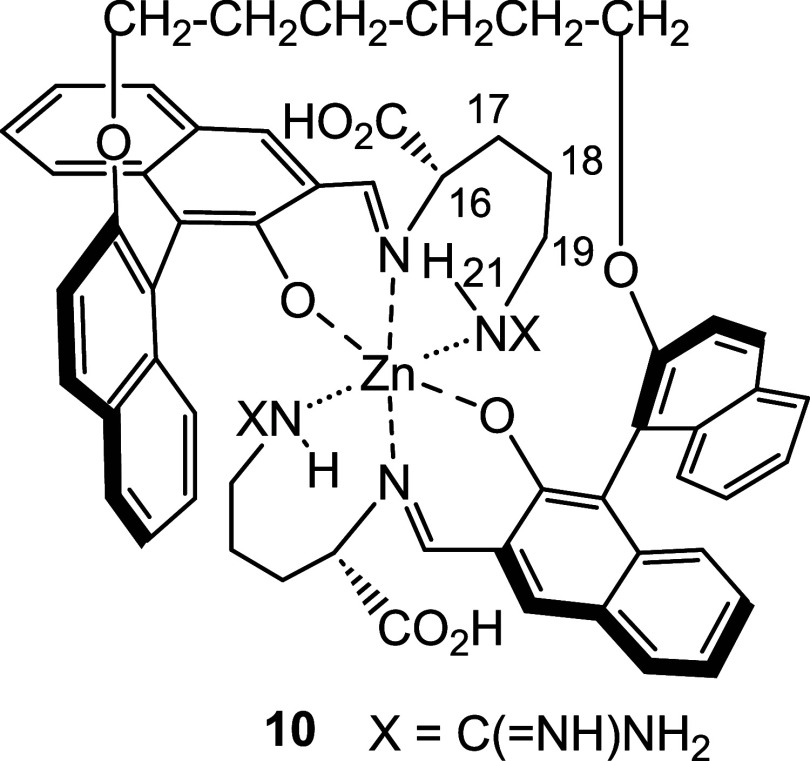
A Proposed Structure of the Zinc Complex **10** from **9**_**D**_+Zn^2+^

A NMR spectroscopic study on the reaction of the imine compound **9**_L_ with various equivalencies of Zn­(OAc)_2_ was also conducted. As shown in Figure 7, when the imine compound **9**_L_ was treated with 0.5–4 equiv Zn­(OAc)_2_, more than one product might be generated with probably one major product. The gHSQC (Figure S40), gNOESY (Figure S41) and gTOCSY (Figure S42) spectra of the product give patterns similar to those observed for **9**_D_+Zn­(OAc)_2_. In the high resolution mass spectrum (TOF, ES-) for the reaction mixture of **9**_L_ + Zn­(OAc)_2_ (See Figure S43 in SI), a weak signal at *m*/*z* = 1085.40 was found for **9**_L_+Zn–H (calcd for C_60_H_61_N_8_O_8_Zn: 1085.39). Another signal at *m*/*z* = 1067.48 was found for **9**_L_+Zn–H_2_O (calcd for C_60_H_59_N_8_O_7_Zn: 1067.38). It is proposed that the products formed from the reaction of **9**_L_ with Zn­(OAc)_2_ might contain structures similar to **10** but should also contain other different structures. Our study demonstrates that the mixture of **9**_L_+Zn­(OAc)_2_ emits more strongly than that of **9**_D_+Zn­(OAc)_2_. This indicates that the proposed structure **10** for **9**_D_+Zn­(OAc)_2_ should be less fluorescent than certain structures generated from **9**_L_+Zn­(OAc)_2_.

**Figure 7:**
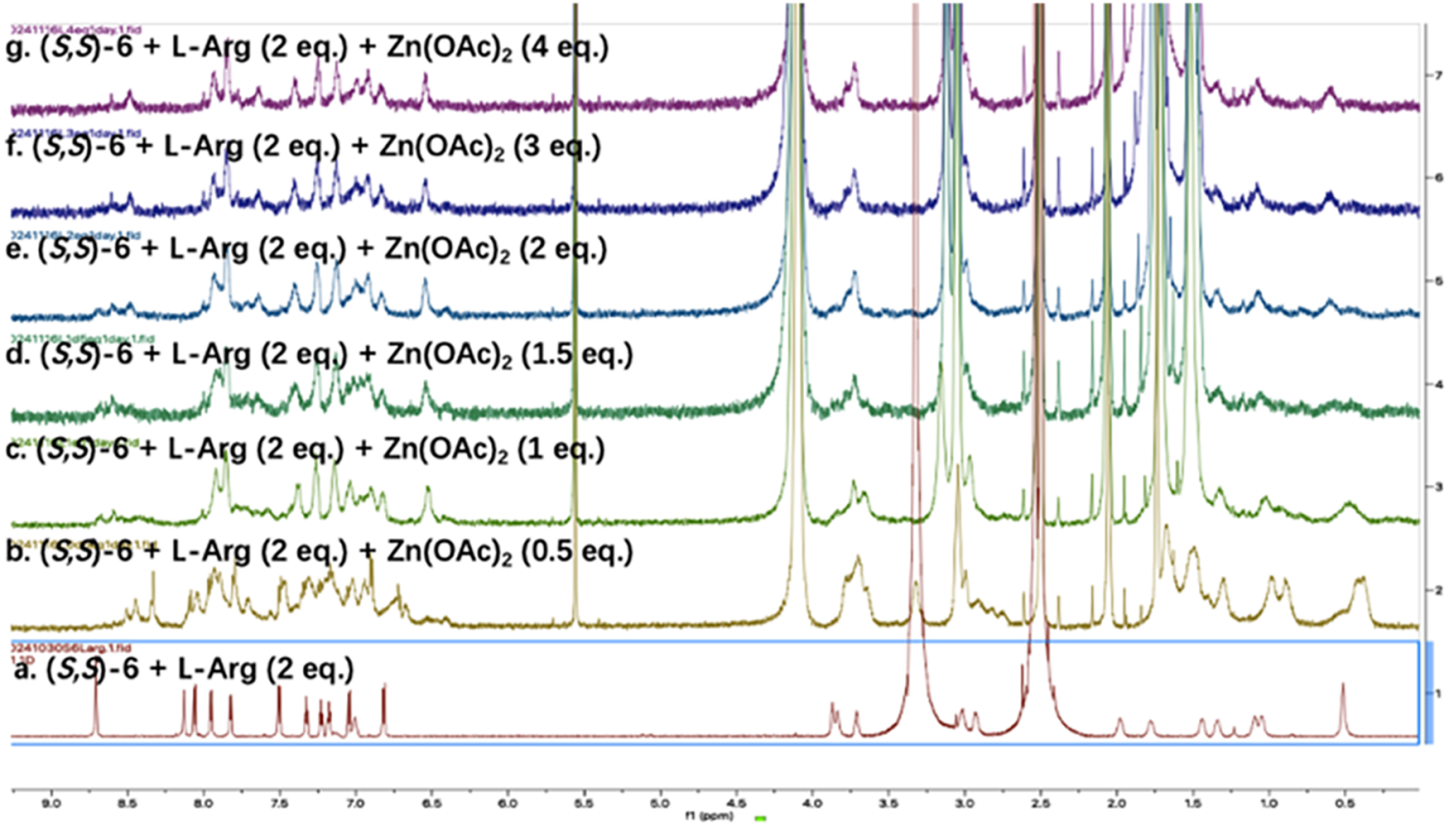
^1^H NMR spectra of (a) (*S,S*)-**6**+l-Arg and (b–g) the subsequent reaction with Zn­(OAc)_2_ in DMSO-*d*_6_ (17% D_2_O) (See the full spectra in Figure S51 in SI).

### 2.4 Comparison of the BisBINOL Probe (*S,S*)-6 with a MonoBINOL Compound in the Fluorescent Recognition of Amino Acids

We have synthesized the monoBINOL compound (*S*)-**11** (Chart 5)^23^ from the reaction of (*S*)-**4** with bromoethane followed by hydrolysis. The fluorescence response of (*S*)-**11** toward various amino acids in the presence of Zn­(OAc)_2_ was investigated. Figure 8 displays the fluorescence spectra of (*S*)-**11** (0.25 mM) with a variety of amino acid enantiomers (10.0 equiv) and Zn­(OAc)_2_ (1.0 equiv) in DMSO and 60% pH = 7.0 phosphate buffer while excited at 405 nm. It shows that this monoBINOL probe has much lower enantioselectivity and chemoselectivity than the bisBINOL probe (*S*,*S*)-**6** for the fluorescent recognition of arginine. Thus, the cooperation of the two BINOL units in (*S*,*S*)-**6** is important for the observed highly chemoselective and enantioselective fluorescent response.

**Figure 8:**
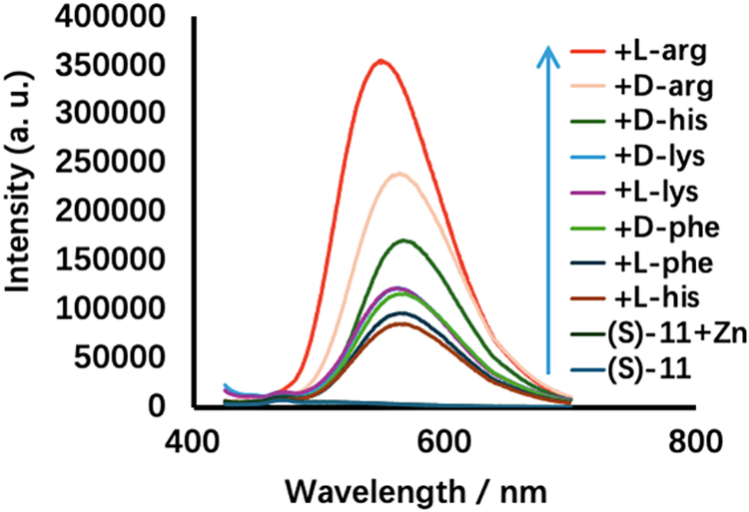
Fluorescence spectra of (*S*)-**11** (0.25 mM) in the presence of Zn­(OAc)_2_ (1.0 equiv) and various amino acids (λ_exc_ = 405 nm, solvent: DMSO/60% pH 7.0 phosphate buffer. Slit: 5/5 nm.).

**Chart 5:**
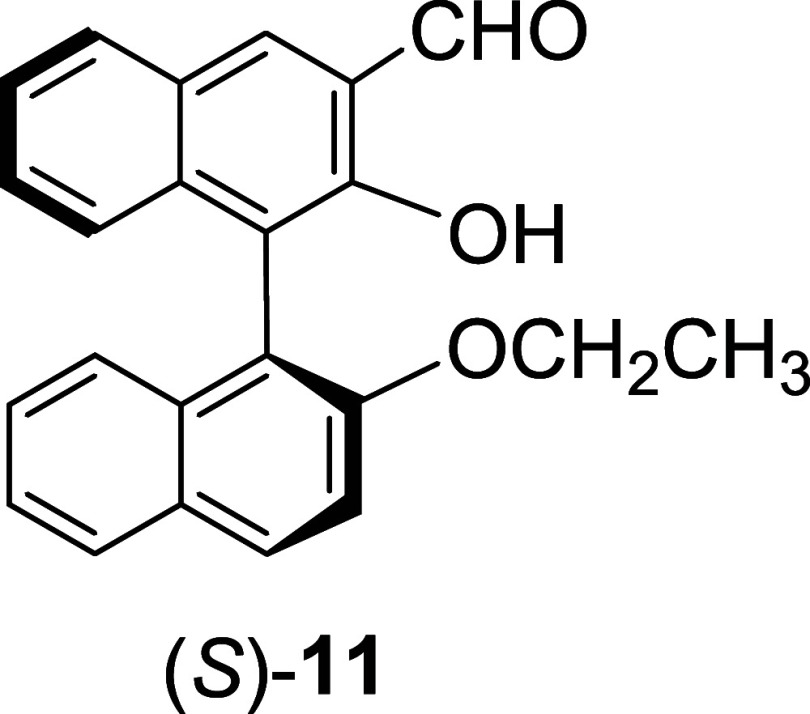
A monoBINOL Compound (*S*)-**11**

## 3 Summary

We have demonstrated that the length of the alkane group linking the two BINOL units of a series of bisBINOL-based compounds strongly influences their fluorescent response toward amino acids. It is found that compound (*S,S*)-**6** containing a 6-carbon alkyl chain linker shows much better sensitivity and selectivity than the other bisBINOL compounds of longer or shorter alkyl chain linkers in the fluorescent recognition of amino acids. This compound in combination with Zn­(OAc)_2_ exhibits highly chemoselective and enantioselective fluorescence enhancement in the presence of arginine but little response toward other common amino acids and their enantiomers. The NMR study shows that (*S,S*)-**6** reacts with arginine to generate a diimine product which can form Zn­(II) complexes. The structural rigidity of these Zn­(II) complexes might have contributed to the observed chemoselective and enantioselective fluorescence enhancement. The study of a monoBINOL analog demonstrates that the cooperation of the two BINOL units in (*S,S*)-**6** is essential for the observed selective fluorescent response.

## 4 Experimental Section

### 4.1 General Data

The commercially available chemicals were purchased from Sigma-Aldrich Chemical Co., Ambeed, Fisher Scientific, or TCI America. All reactions were set up under N_2_. All of the solvents used for reactions are anhydrous and stored in the Glove Box under N_2_. In the fluorescence measurements, all solvents were ACS, HPLC or spectroscopic grades. NMR spectra were recorded on a Varian-600 MHz spectrometer, a Bruker-400 MHz spectrometer, a Bruker-600 MHz spectrometer or a Bruker-800 MHz spectrometer. Optical rotation measurements were conducted by using a Jasco P-2000 digital polarimeter. Chemical shifts for ^1^H NMR spectra were measured in parts per million relative to solvent signals at 7.26 ppm for CDCl_3_ and 2.50 ppm for DMSO-*d*_6_. Chemical shifts for ^13^C NMR were measured relative to the centerline of a triplet at 77.16 ppm for CDCl_3_ and a septet at 39.52 ppm for DMSO-*d*_6_. Structural assignments were made with additional information from gHSQC, gNOESY and gTOCSY experiments. High-resolution mass spectra were obtained from the University of Illinois at Urbana–Champaign (UIUC) Mass Spectrometry Facility and the University of California Riverside Mass Spectrometry Facility. Steady-state fluorescence spectra were measured with a Horiba FluoroMax-4 spectrofluorometer. UV–vis spectra were measured by Shimadzu UV-2600 UV–vis spectrometer.

Based on a reported protocol,^24^ absolute quantum yield (Φ) values were directly determined using an integrating sphere. A (*S*,*S*)-**6** solution (1 μM, DMSO) was prepared with an maximum absorbance of 0.1 in the UV–vis spectrum. A SuperK EXTREME EXU-6 white laser (repetition rate: 77.79 MHz) was used as the excitation source and an InGaAs NIR detector was used to acquire fluorescence emission.

#### 4.1.1 A General Procedure for the Preparation of (*S*,*S*)-**5**, (*S*,*S*)-**6**, (*S*,*S*)-**7**, and (*S*,*S*)-**8**

(*S*)-**4** (0.5 g, 1.38 mmol, 2.5 equiv) and anhydrous K_2_CO_3_ (1.2 g, 8.80 mmol, 16 equiv) were dissolved in acetonitrile (15 mL) under N_2_. 1,4-Dibromobutane (0.06 mL, 0.55 mmol), or 1,6-dibromohexane (0.08 mL, 0.55 mmol), or 1,8-dibromooctane (0.10 mL, 0.55 mmol), or 1,10-dibromodecane (0.12 mL, 0.55 mmol) was added slowly at room temperature. The mixture was heated under reflux at 90 °C (oil bath temperature) with stirring for 16 h and the solvent was then removed by rotoevaporation. The residue was dissolved in methylene chloride or ethyl acetate (50 mL) and washed with brine (3 × 50 mL) for 3 times, and then dried over Na_2_SO_4_. After evaporation of the solvents by rotavapor, the residue was purified by column chromatography on silica gel eluted with 20% ethyl acetate in hexane. The product was dissolved in 50 mL anhydrous methylene chloride under N_2_. Trifluoroacetic acid (0.25 mL, 3.25 mmol, 5.9 equiv) was added dropwise into the solution at room temperature. The mixture was stirred under N_2_ for 2 h and brine (3 × 50 mL) was used to wash the organic phase for 3 times which was then dried over Na_2_SO_4_. The solvent was then removed, and the product was purified by column chromatography on silica gel, eluted with CH_2_Cl_2_ to give (*S*,*S*)-**5**, (*S*,*S*)-**6**, (*S*,*S*)-**7**, or (*S*,*S*)-**8** as light-yellow solid.

#### 4.1.2 Characterization of (*S*,*S*)-**5**

(*S*,*S*)-**5** (338 mg) was obtained as a yellow solid in 90% yield (eluted with CH_2_Cl_2_ by column chromatography on silica gel). ^1^H NMR (CDCl_3_, 600 MHz) δ 10.36 (s, 2H), 10.07 (s, 2H), 8.15 (s, 2H), 7.93 (d, 2H), 7.88 (d, 4H), 7.38–7.19 (m, 10H), 7.13 (d, 2H), 7.06 (d, 2H), 3.73–3.54 (m, 4H), 1.17–0.91 (m, 4H). ^13^C­{^1^H} NMR (150 MHz, CDCl_3_) δ 196.9, 154.5, 153.5, 138.0, 137.8, 133.7, 130.2, 130.1, 129.8, 129.4, 128.3, 127.5, 126.8, 125.5, 125.0, 124.2, 123.9, 122.1, 118.8, 118.1, 115.6, 68.8, 25.4. HRMS: *m*/*z* calcd. for C_46_H_35_O_6_ [M+H^+^]: 683.2434, found 683.2462. Mp 230–235 °C. [α]_D_^25^ = −137.3 (*c* = 0.2, CH_2_Cl_2_).

#### 4.1.3 Characterization of (*S*,*S*)-**6**

(*S,S*)-**6** (332 mg) was obtained as a yellow solid in 85% yield (eluted with CH_2_Cl_2_ by column chromatography on silica gel). ^1^H NMR (CDCl_3_, 600 MHz) δ 10.41 (s, 2H), 10.05 (s, 2H), 8.09 (s, 2H), 8.00 (d, 2H), 7.91 (d, 2H), 7.77 (d, 2H), 7.46–7.32 (m, 4H), 7.32–7.14 (m, 8H), 7.12–7.01 (m, 2H), 3.89–3.75 (m, 4H), 1.24–1.00 (m, 4H), 0.70–0.52 (m, 4H). ^13^C­{^1^H} NMR (150 MHz, CDCl3) δ 197.0, 154.7, 153.5, 138.0, 137.8, 133.8, 130.3, 130.1, 129.8, 129.5, 128.3, 127.6, 126.8, 125.4, 125.0, 124.2, 123.9, 122.1, 118.9, 118.2, 115.6, 69.4, 29.2, 25.2. HRMS: *m*/*z* calcd. for C_48_H_39_O_6_ [M+H^+^]: 711.2747, found 711.2766. Mp 220–223 °C. [α]_D_^25^ = −151.2 (*c* = 0.2, CH_2_Cl_2_). Fluorescence quantum yield: Φ (%) = 0.17 (DMSO).

#### 4.1.4 Characterization of (*R,R*)-**6**

(*R,R*)-**6** (336 mg) was obtained as a yellow solid in 86% yield (eluted with CH_2_Cl_2_ by column chromatography on silica gel). ^1^H NMR (CDCl_3_, 600 MHz) δ 10.41 (s, 2H), 10.05 (s, 2H), 8.09 (s, 2H), 8.00 (d, 2H), 7.91 (d, 2H), 7.77 (d, 2H), 7.46–7.32 (m, 4H), 7.32–7.14 (m, 8H), 7.12–7.01 (m, 2H), 3.89–3.75 (m, 4H), 1.24–1.00 (m, 4H), 0.70–0.52 (m, 4H). Mp 219–223 °C. [α]_D_^25^ = +152.4 (*c* = 0.2, CH_2_Cl_2_).

#### 4.1.5 Characterization of (*S,S*)-**7**

(*S,S*)-**7** (350 mg) was obtained as a yellow solid in 86% yield (eluted with CH_2_Cl_2_ by column chromatography on silica gel). ^1^H NMR (CDCl_3_, 600 MHz) δ 10.43 (s, 2H), 10.09 (s, 2H), 8.15 (s, 2H), 8.02 (d, 2H), 7.92 (d, 2H), 7.83 (d, 2H), 7.47 (d, 2H), 7.37 (t, 2H), 7.30–7.26 (m, 6H), 7.18 (d, 2H), 7.15 (d, 2H), 4.06–3.90 (m, 4H), 1.42–1.29 (m, 4H), 0.82–0.64 (m, 8H). ^13^C­{^1^H} NMR (150 MHz, CDCl3) δ 197.0, 154.8, 153.6, 138.1, 137.8, 133.8, 130.3, 130.2, 129.8, 129.5, 128.3, 127.6, 126.8, 125.5, 125.0, 124.3, 123.9, 122.1, 119.0, 118.2, 115.7, 69.7, 29.3, 28.9, 25.5. HRMS: *m*/*z* calcd. for C_50_H_43_O_6_ [M+H^+^]: 739.3060, found 739.3071. Mp 95–102 °C. [α]_D_^25^ = −163.9 (*c* = 0.2, CH_2_Cl_2_).

#### 4.1.6 Characterization of (*S,S*)-**8**

(*S,S*)-**8** (371 mg) was obtained as a yellow solid in 88% yield (eluted with CH_2_Cl_2_ by column chromatography on silica gel). ^1^H NMR (CDCl_3_, 600 MHz) δ 10.41 (s, 2H), 10.11 (s, 2H), 8.20 (s, 2H), 7.99 (d, 2H), 7.92–7.85 (m, 4H), 7.45 (d, 2H), 7.37–7.27 (m, 6H), 7.26–7.22 (m, 2H), 7.20–7.13 (m, 4H), 4.08–3.93 (m, 4H), 1.46–1.36 (m, 4H), 0.95–0.80 (m, 12H). ^13^C­{^1^H} NMR (150 MHz, CDCl_3_) δ 196.9, 154.9, 153.4, 138.1, 137.8, 133.8, 130.3, 130.1, 129.8, 129.5, 128.3, 127.6, 126.8, 125.6, 125.0, 124.3, 123.9, 122.2, 119.0, 118.3, 115.8, 70.0, 29.5, 29.3, 29.1, 25.7. HRMS: *m*/*z* calcd. for C_52_H_47_O_6_ [M+H^+^]: 767.3373, found 767.3373. Mp 90–95 °C. [α]_D_^25^ = −152.8 (*c* = 0.2, CH_2_Cl_2_).

#### 4.1.7 Preparation of (*S*)-**11**

(*S*)-**4** (0.3 g, 0.84 mmol) and anhydrous K_2_CO_3_ (730 mg, 5.3 mmol, 6.3 equiv) were dissolved in acetonitrile (15 mL) under N_2_. Bromoethane (0.07 mL, 0.93 mmol, 1.1 equiv) was added slowly at room temperature. The mixture was heated under reflux at 90 °C (oil bath temperature) with stirring for 16 h and the solvent was then removed by rotoevaporation. The mixture was dissolved in methylene chloride or ethyl acetate (30 mL) and washed with brine (3 × 30 mL) for 3 times, and then dried over Na_2_SO_4_. After evaporation of the solvents by rotavapor, the product was obtained as a colorless to very light-yellow oil in 97% yield. The product was dissolved in anhydrous methylene chloride (10 mL) under N_2_. Trifluoroacetic acid (0.38 mL, 5.0 mmol, 5.9 equiv) was added dropwise into the solution at room temperature. The mixture was stirred under N_2_ for 1 h and brine (3 × 30 mL) was used to wash the organic phase for 3 times which was dried over Na_2_SO_4_. The solvent was then removed, and the product (*S*)-**11** was obtained as light-yellow crystals in 99% yield (283 mg).

#### 4.1.8 Characterization of (*S*)-**11**

^1^H NMR (CDCl_3_, 600 MHz) δ 10.41 (s, 1H), 10.20 (s, 1H), 8.30 (s, 1H), 8.00–7.95 (m, 2H), 7.88 (d, 1H), 7.45 (d, 1H), 7.39–7.35 (m, 2H), 7.33 (t, 1H), 7.25 (t, 1H), 7.20–7.16 (m, 1H), 7.15 (d, 1H), 4.14–4.04 (m, 2H), 1.06 (t, 3H). ^13^C­{^1^H} NMR (150 MHz, CDCl_3_) δ 196.9, 154.7, 153.6, 138.1, 137.9, 133.8, 130.3, 130.2, 129.8, 129.5, 128.3, 127.7, 126.7, 125.6, 125.0, 124.3, 123.9, 122.2, 119.0, 118.4, 115.9, 65.5, 15.1. Mp 135–138 °C. [α]_D_^25^ = – 169.6 (*c* = 0.2, CH_2_Cl_2_).

### 4.2 A General Procedure for the Preparation of Samples for Fluorescence Measurement

BisBINOL compounds (*S,S*)-**5** – (*S*,S)-**8** in DMSO (1.2 mL, 0.625 mM), Zn­(OAc)_2_ in deionized H_2_O (0.9 mL, 0.833 mM), and amino acids in 12.5 mM pH 7.0 phosphate buffer (0.9 mL, 8.333–50 mM) were freshly prepared and mixed for each measurement. The total volume was maintained at 3 mL (DMSO/H_2_O = 1:1.5) for each sample. The solution was shaken to react at room temperature for 4 h, unless otherwise noted.

### 4.3 A General Procedure for the Preparation of Samples for NMR Spectroscopic Study

The following stock solutions were freshly prepared for each measurement: (*S,S*)-**6** (9.6 mM) in DMSO-*d*_6_, Zn­(OAc)_2_ (4.8–38.4 mM) in DMSO-*d*_6_, and d- or l-Arg (48.0 mM) in D_2_O. In each measurement, 250 μL of (*S,S*)-**6**, 100 μL of d-/l-Arg (2.0 equiv), and 250 μL of Zn­(OAc)_2_ (0–4.0 equiv) in DMSO-*d*_6_ were mixed and reacted for 16 h (or as noted) [The final concentration of (*S*,*S*)-**6**: 4.0 mM].

## Supplementary Material



## Data Availability

The data underlying this study are available in the published article and its Supporting Information.

## Notes

The spectra of 1D/2D NMR and mass analysis data; Plots of fluorescence competitive experiments and limit of detection study (PDF)
