# Supplementary material for: BisBINOL-Based Fluorescent Probes: Effect of Alkane Linkers and Chemo- and Enantioselective Recognition of Arginine
Source: J Org Chem. 2025 Nov 5;90(45):16007–16. doi: 10.1021/acs.joc.5c01699 (PMC12624842; doi:10.1021/acs.joc.5c01699)
Supplement: Supplementary file 1 [file jo5c01699_si_001.pdf]

Supporting information for

BisBINOL-Based Fluorescent Probes: Effect of Alkane Linkers and Chemo- and Enantioselective Recognition of Arginine

Yichen Li, Carson Patrick, Franklin He, Sydney Rohrbach, Yifan Mao, and Lin Pu*

Department of Chemistry, University of Virginia, Charlottesville, Virginia 22904, USA.

E-mail: lp6n@virginia.edu

Table of contents

1. ^{1}H NMR, ^{13}C{^{1}H} NMR, gHSQC, gNOESY and HRMS Spectra of (*S,S*)-**5,** (*S,S*)-**6**, (*S,S*)-**7**, (*S,S*)-**8**, and (*S*)-**11**
2. NMR and Mass Spectral Studies on the Reaction of (*S,S*)-**6** with Arginine in DMSO-d_6/17% D_2O
   2.1. NMR and mass spectra of (*S,S*)-**6** with D-Arg
   2.2. NMR and mass spectra of (*S,S*)-**6** with L-Arg
3. NMR and Mass Spectral Studies on the Reaction of (*S,S*)-**6** with Arginine and $Zn(OAc)_2$ in DMSO-d_6/14% D_2O
   3.1. NMR and mass spectra of (*S,S*)-**6** + 2 eq. D-Arg with 1 eq. $Zn(OAc)_2$
   3.2. NMR and mass spectra of (*S,S*)-**6** with+ 2 eq. L-Arg with 1 eq. $Zn(OAc)_2$
   3.3. NMR of (*S,S*)-**6**, L-Arg and $Zn(OAc)_2$
4. NMR Spectroscopic Study on the Reaction of (*S,S*)-**6** + Arginine in DMSO-d_6/17% D_2O with $Zn(OAc)_2$
   4.1. ^{1}H NMR spectra of (*S,S*)-**6** +2 eq. D-Arg with addition of various equivalents of $Zn(OAc)_2$
   4.2. ^{1}H NMR spectra of (*S,S*)-**6** + 2 eq. L-Arg with addition of various equivalents of $Zn(OAc)_2$
5. Fluorescence Competitive Study
6. Limit of Detection of L-Arg by (*S,S*)-**6**

1. ^{1}H NMR, $^{13}C\{^{1}H\}$ NMR, gHSQC, gNOESY and HRMS Spectra of (*S,S*)-5, (*S,S*)-6, (*S,S*)-7, (*S,S*)-8, (*S*)-11

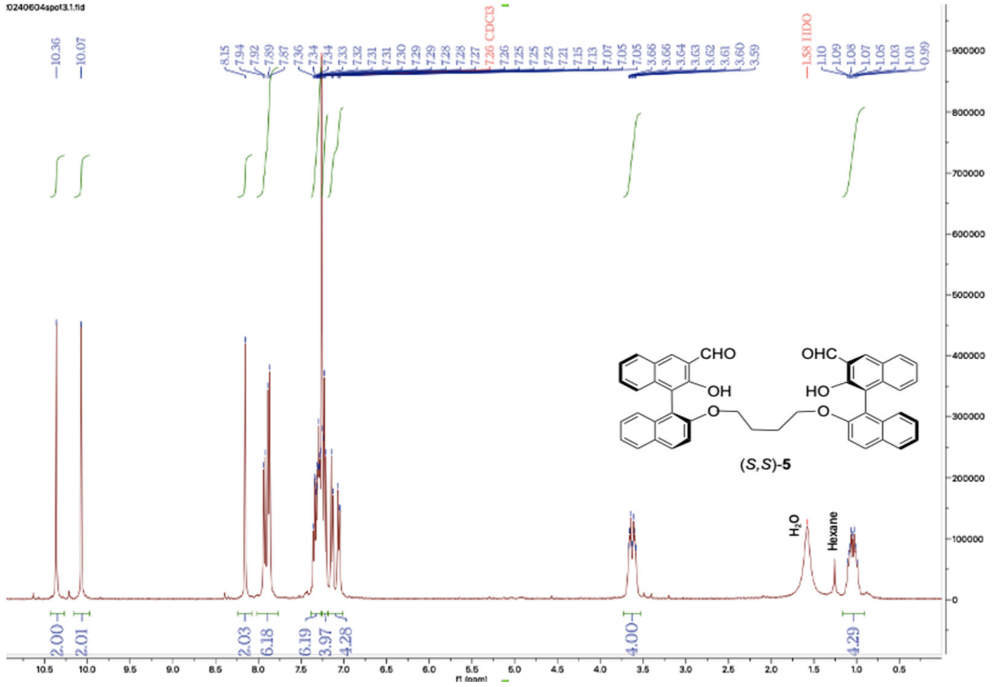

Figure S1. ^{1}H NMR spectrum of (*S,S*)-**5** in $CDCl_3$. (600 MHz)

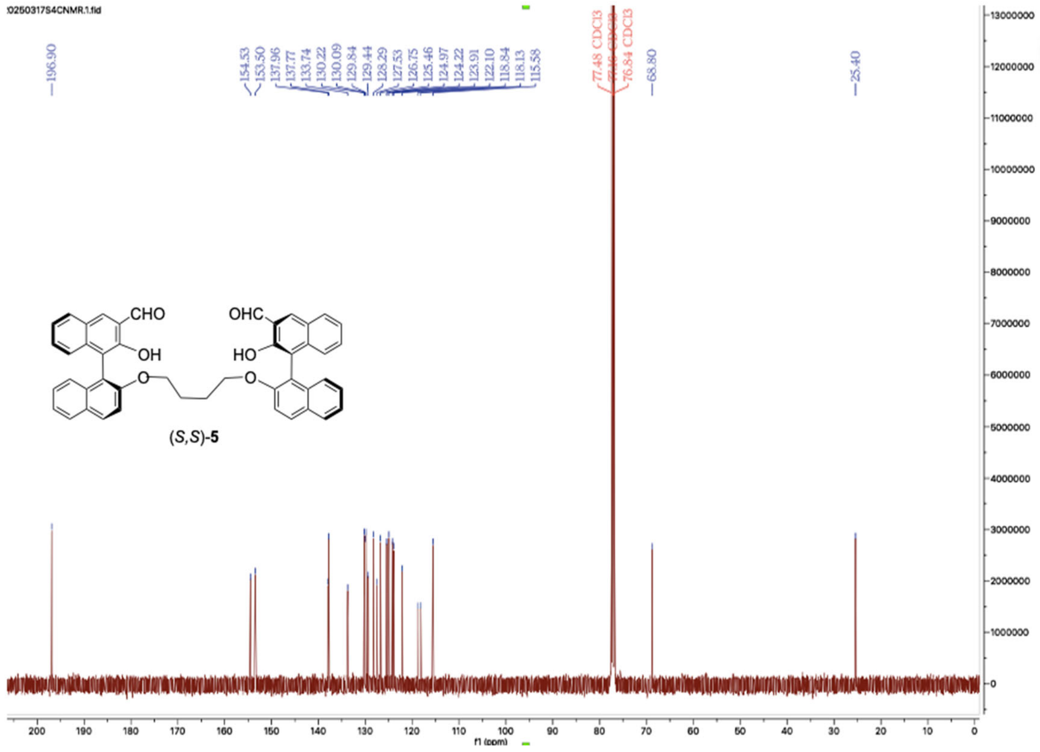

Figure S2. $^{13}C\{^{1}H\}$ NMR (150 MHz, $CDCl_3$) spectrum of (*S,S*)-**5** in $CDCl_3$.

HRMS of (*S,S*)-**5**

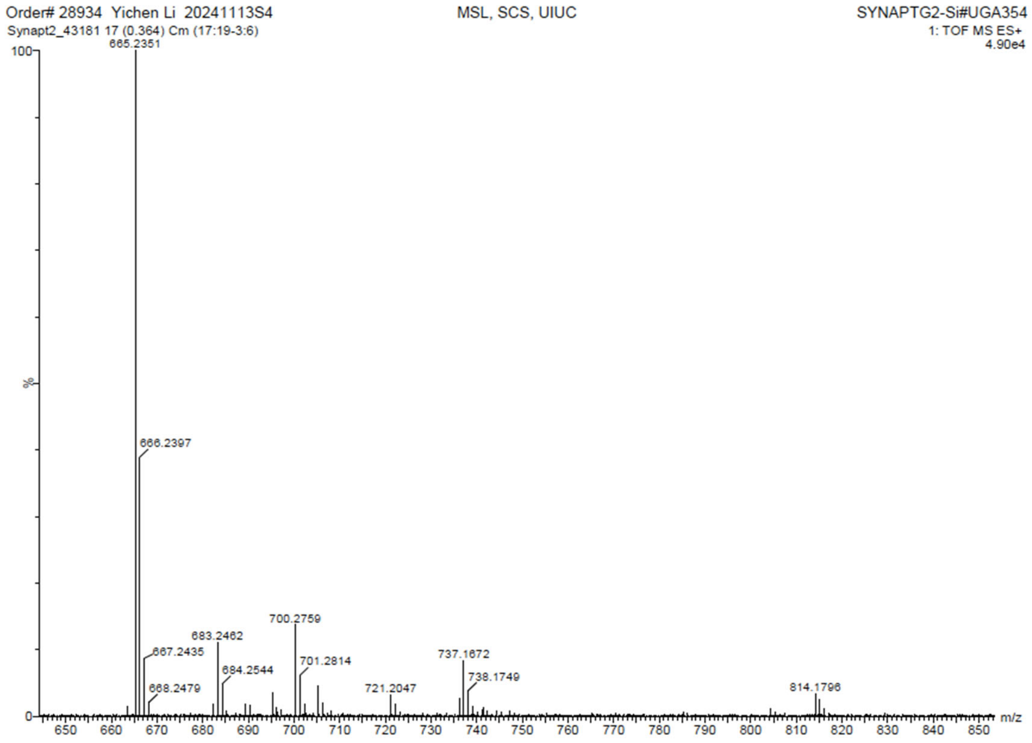

Elemental Composition Report

Page 1

Single Mass Analysis

Tolerance = 5.0 PPM / DBE: min = -1.5, max = 100.0
Element prediction: Off
Number of isotope peaks used for i-FIT = 8

Monoisotopic Mass, Even Electron Ions
682 formula(e) evaluated with 4 results within limits (up to 50 best isotopic matches for each mass)
Elements Used:
C: 0-70 H: 0-80 N: 0-9 O: 0-9

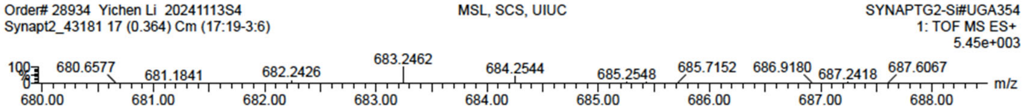

Minimum:				-1.5				
Maximum:		5.0	5.0	100.0				
Mass	Calc. Mass	mDa	PPM	DBE	i-FIT	Norm	Conf(%)	Formula
683.2462	683.2466	-0.4	-0.6	21.5	273.7	0.113	89.28	C35 H35 N6 O9
	683.2447	1.5	2.2	34.5	276.6	3.018	4.89	C47 H31 N4 O2
	683.2434	2.8	4.1	29.5	276.6	3.053	4.72	C46 H35 O6
	683.2487	-2.5	-3.7	38.5	278.1	4.507	1.10	C52 H31 N2

Figure S3. HRMS of (*S,S*)-**5**

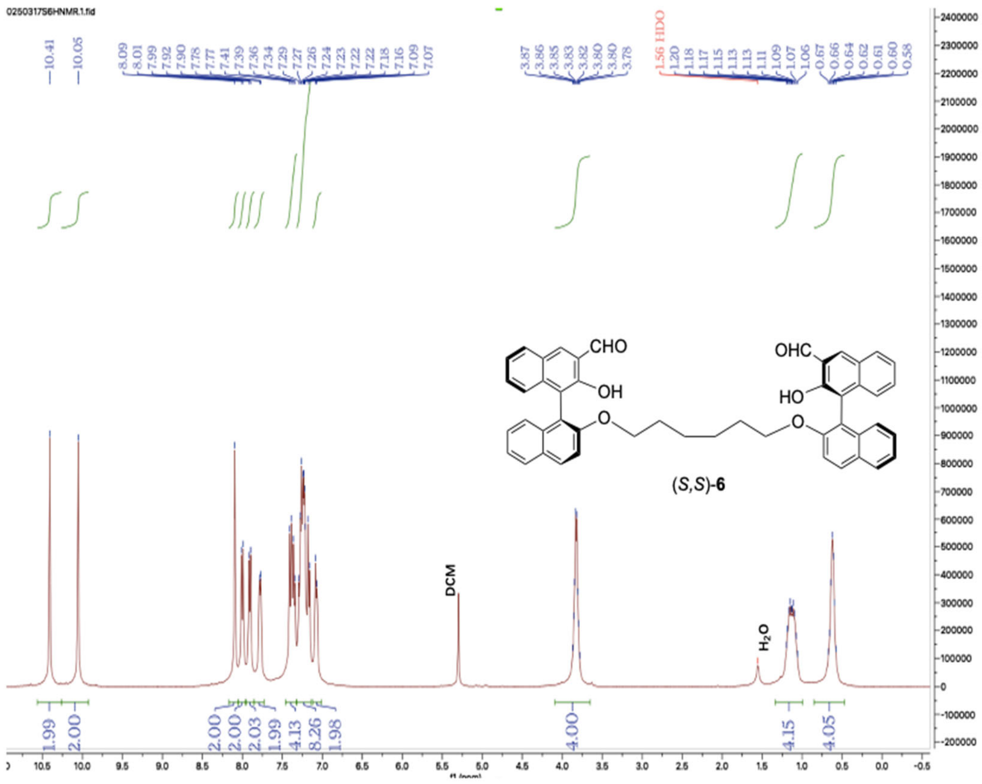

Figure S4. ^{1}H NMR spectrum of (*S,S*)-**6** in $CDCl_3$. (600 MHz)

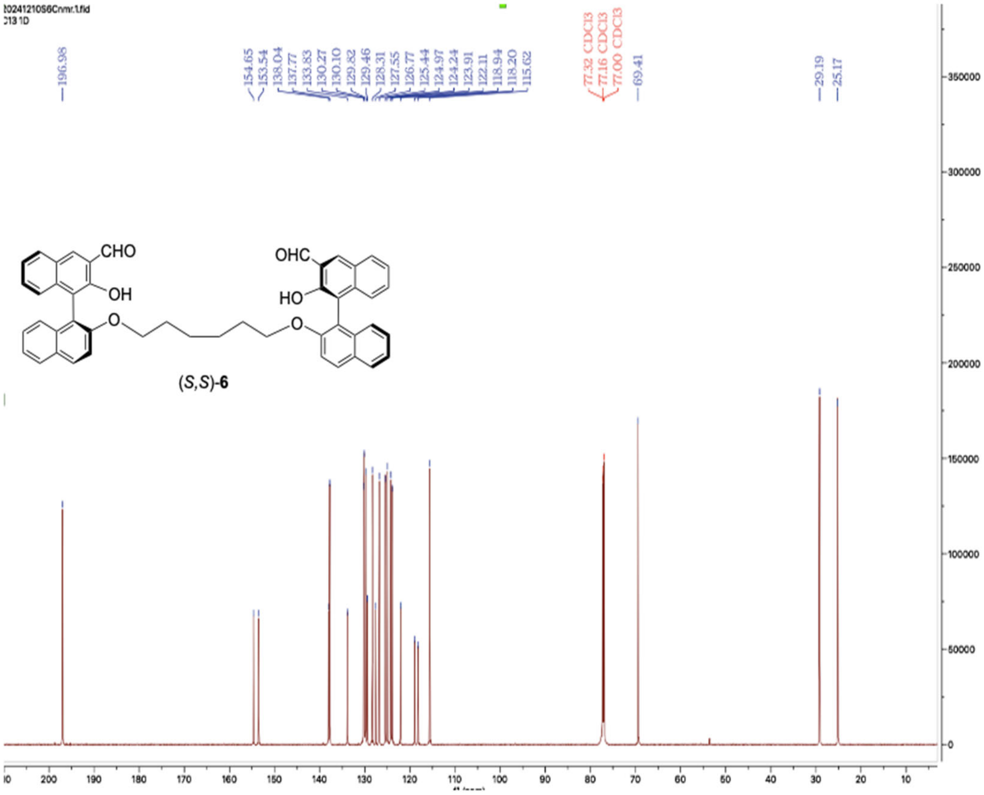

Figure S5. $^{13}C\{^{1}H\}$ NMR (150 MHz, $CDCl_3$) NMR spectrum of (*S,S*)-**6** in $CDCl_3$.

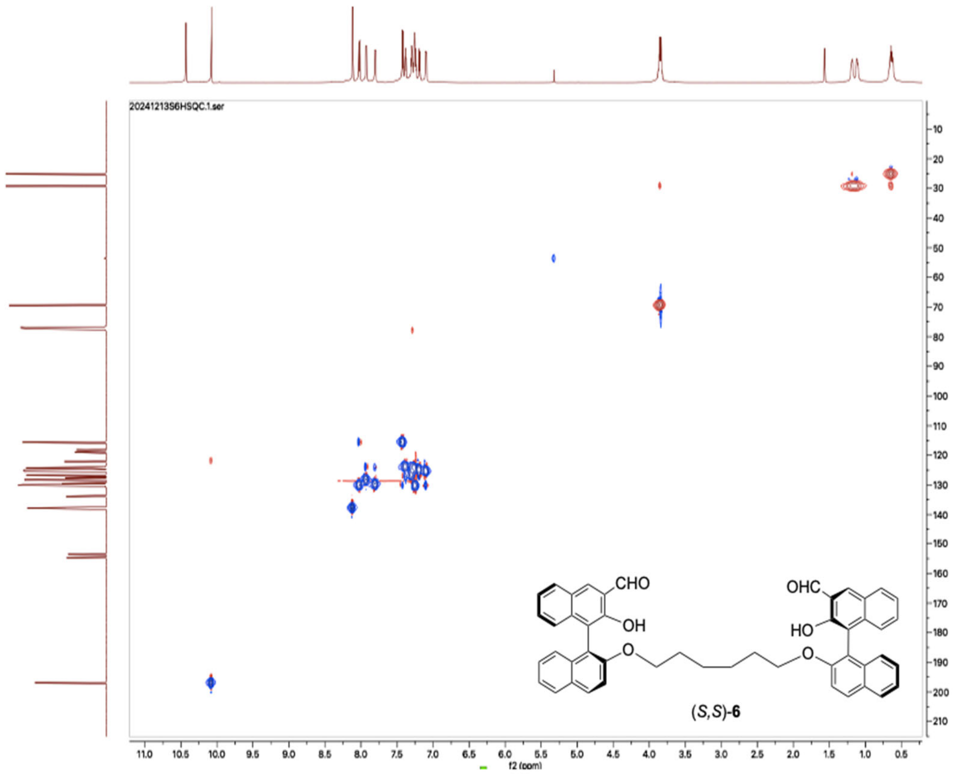

Figure S6. gHSQC spectrum of (*S,S*)-**6** in $CDCl_3$. (600 MHz)

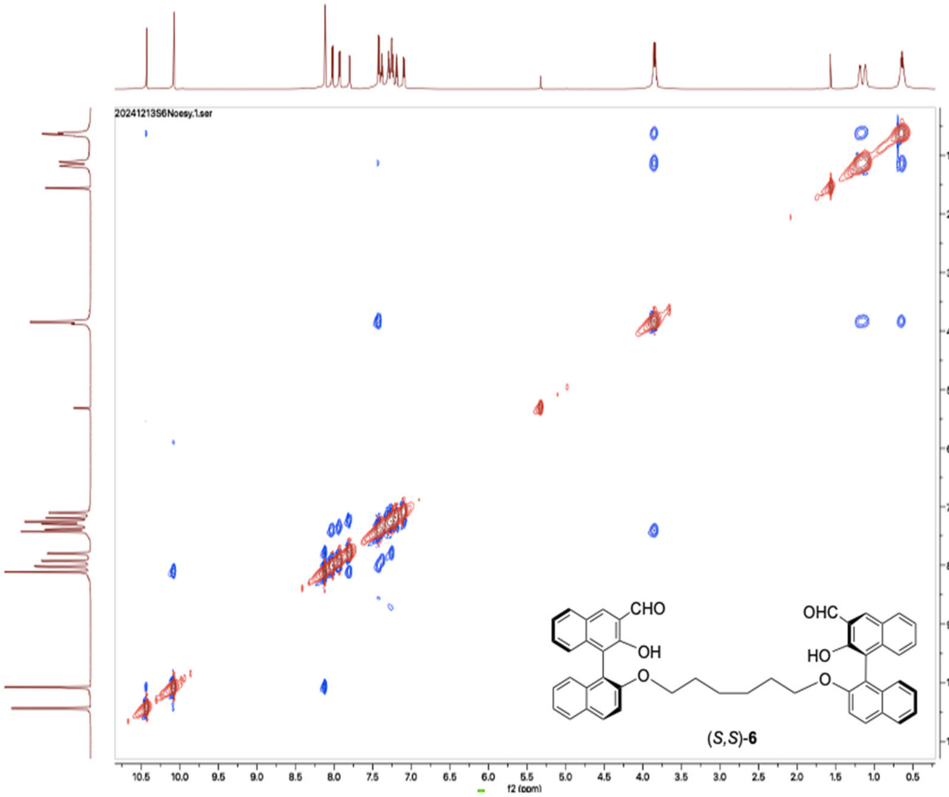

Figure S7. gNOESY spectrum of (*S,S*)-**6** in $CDCl_3$. (600 MHz)

HRMS of (*S*,*S*)-**6**

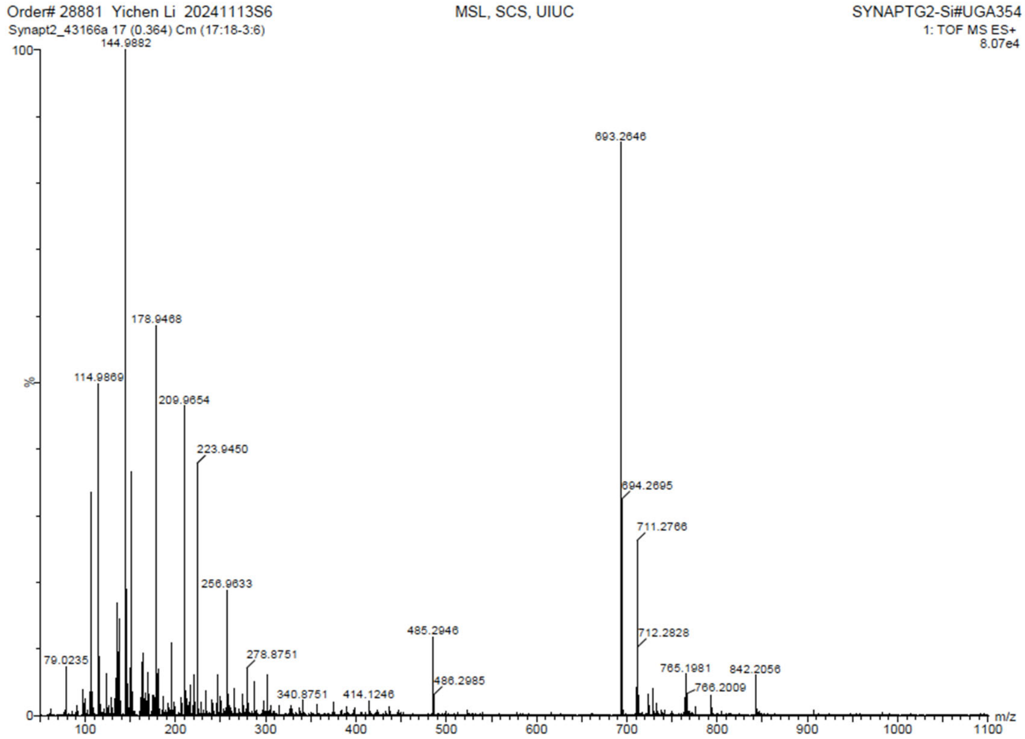

Elemental Composition Report **Page 1**

Single Mass Analysis
Tolerance = 5.0 PPM / DBE: min = -1.5, max = 100.0
Element prediction: Off
Number of isotope peaks used for i-FIT = 8

Monoisotopic Mass, Even Electron Ions
683 formula(e) evaluated with 4 results within limits (up to 50 best isotopic matches for each mass)
Elements Used:
C: 0-70 H: 0-80 N: 0-9 O: 0-9

Order# 28881 Yichen Li 20241113S6 MSL, SCS, UIUC SYNAPTG2-Si#UGA354
Synapt2_43166a 17 (0.364) Cm (17:18-3:6) 1: TOF MS ES+
2.12e+004

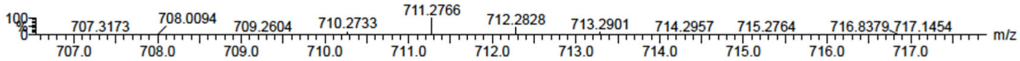

Minimum:				-1.5				
Maximum:		5.0	5.0	100.0				
Mass	Calc. Mass	mDa	PPM	DBE	i-FIT	Norm	Conf(%)	Formula
711.2766	711.2779	-1.3	-1.8	21.5	253.2	0.002	99.75	C37 H39 N6 O9
	711.2747	1.9	2.7	29.5	259.7	6.458	0.16	C48 H39 O6
	711.2760	0.6	0.8	34.5	260.3	7.140	0.08	C49 H35 N4 O2
	711.2800	-3.4	-4.8	38.5	262.3	9.061	0.01	C54 H35 N2

Figure S8. HRMS of (*S*,*S*)-**6**

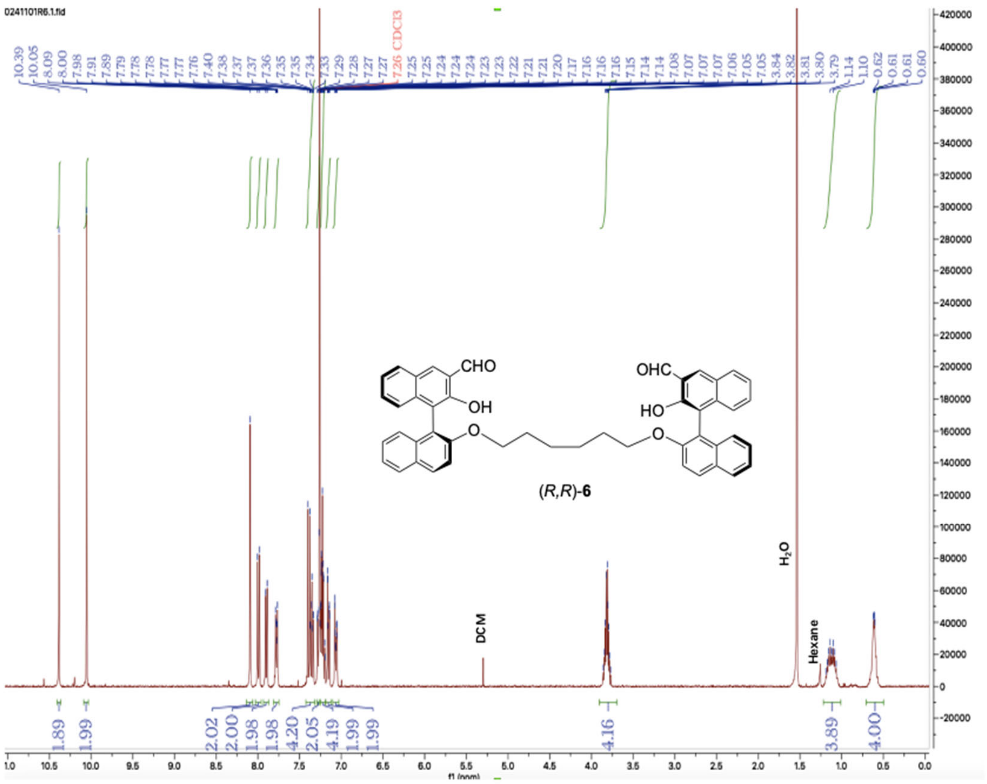

Figure S9. ^{1}H NMR spectrum of (*R*,*R*)-**6** in $CDCl_3$. (600 MHz)

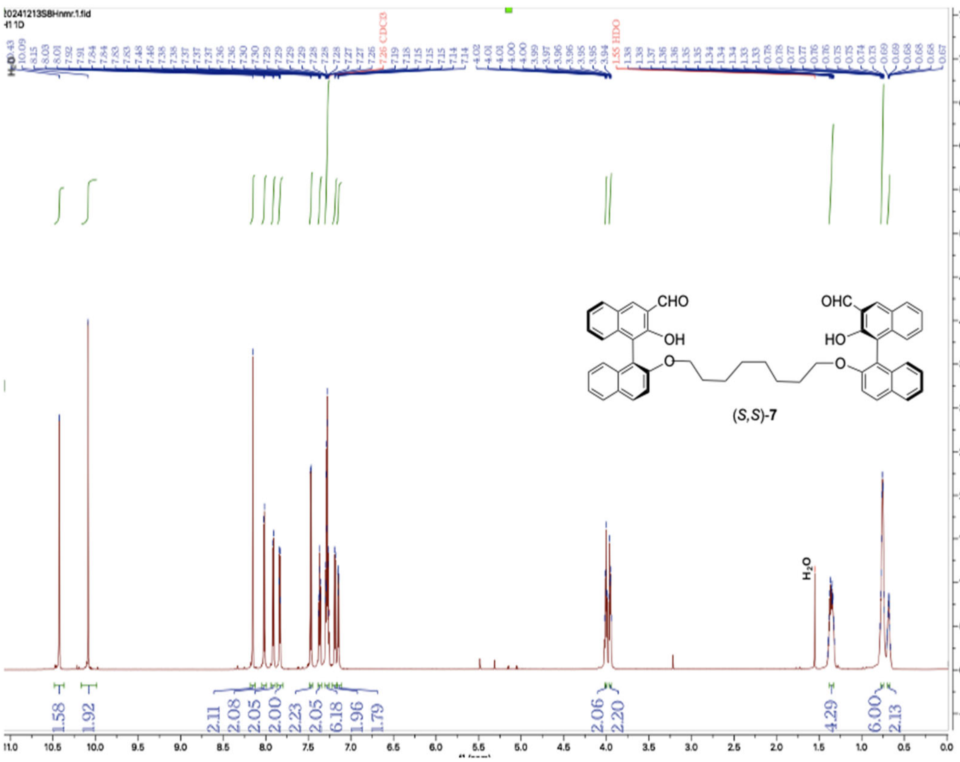

Figure S10. ^{1}H NMR spectrum of (*S*,*S*)-**7** in $CDCl_3$. (600 MHz)

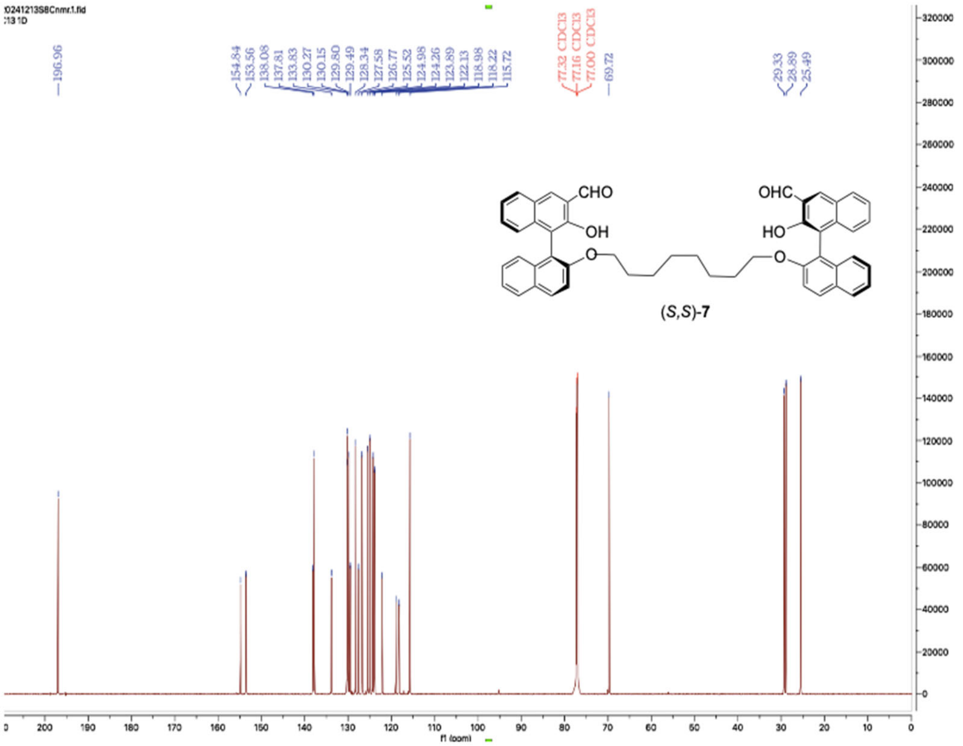

Figure S11. $^{13}C\{^1H\}$ NMR (150 MHz, $CDCl_3$) spectrum of (*S,S*)-**7** in $CDCl_3$.

HRMS of (*S,S*)-**7**

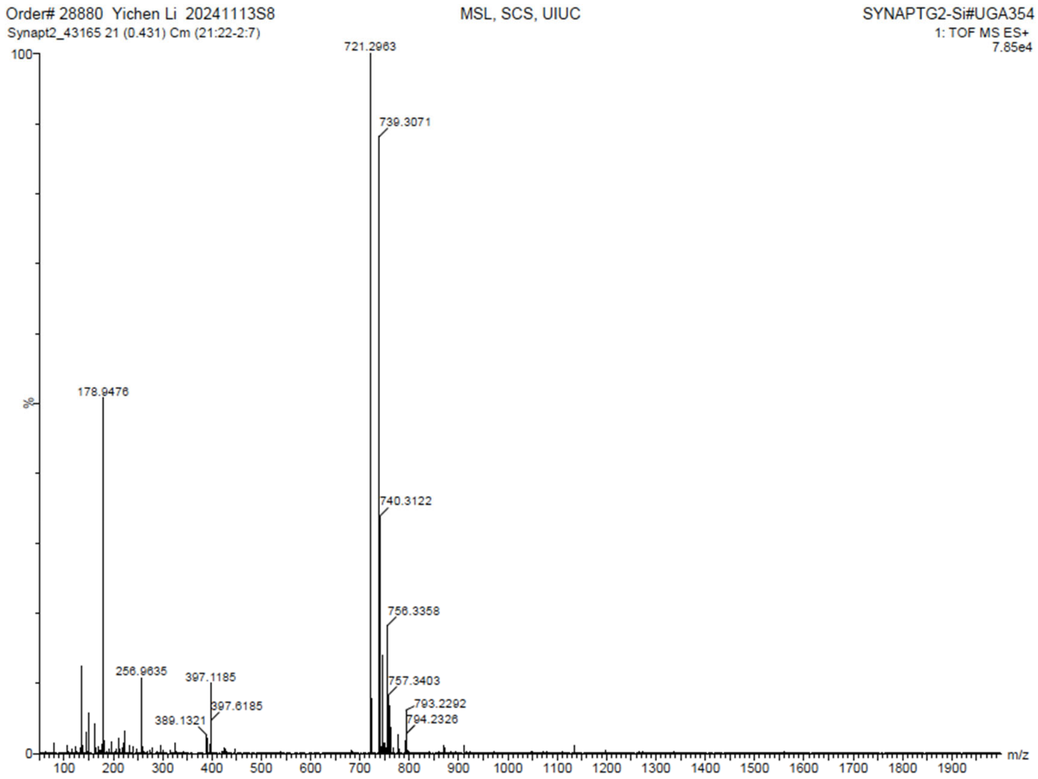

Elemental Composition Report **Page 1**

Single Mass Analysis
Tolerance = 5.0 PPM / DBE: min = -1.5, max = 100.0
Element prediction: Off
Number of isotope peaks used for i-FIT = 8

Monoisotopic Mass, Even Electron Ions
336 formula(e) evaluated with 2 results within limits (up to 50 best isotopic matches for each mass)
Elements Used:
C: 0-70 H: 0-80 N: 0-6 O: 0-6

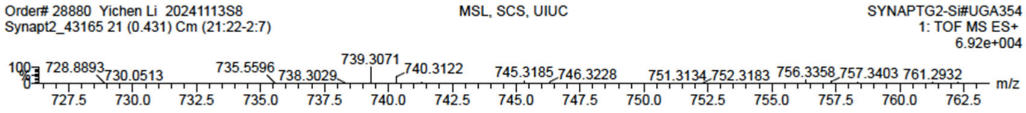

Minimum:				-1.5				
Maximum:		5.0	5.0	100.0				
Mass	Calc. Mass	mDa	PPM	DBE	i-FIT	Norm	Conf(%)	Formula
739.3071	739.3073	-0.2	-0.3	34.5	218.2	0.605	54.60	C51 H39 N4 O2
	739.3060	1.1	1.5	29.5	218.4	0.790	45.40	C50 H43 O6

Figure S12. HRMS of (*S,S*)-**7**

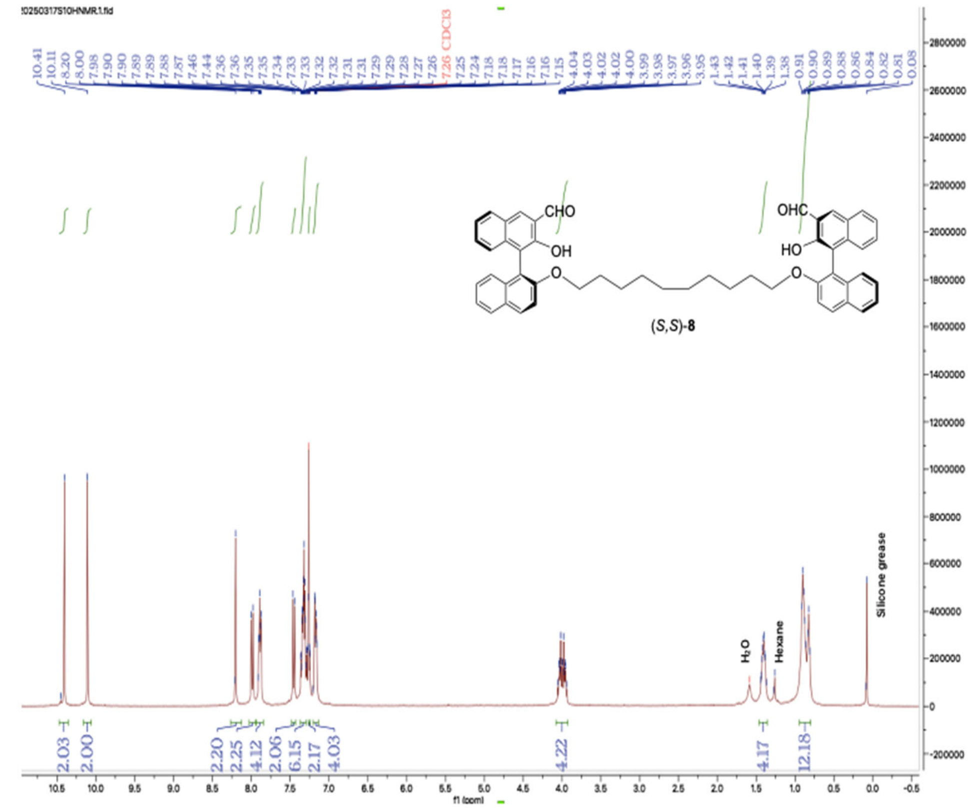

Figure S13. ^{1}H NMR spectrum of (*S,S*)-**8** in $CDCl_3$. (600 MHz)

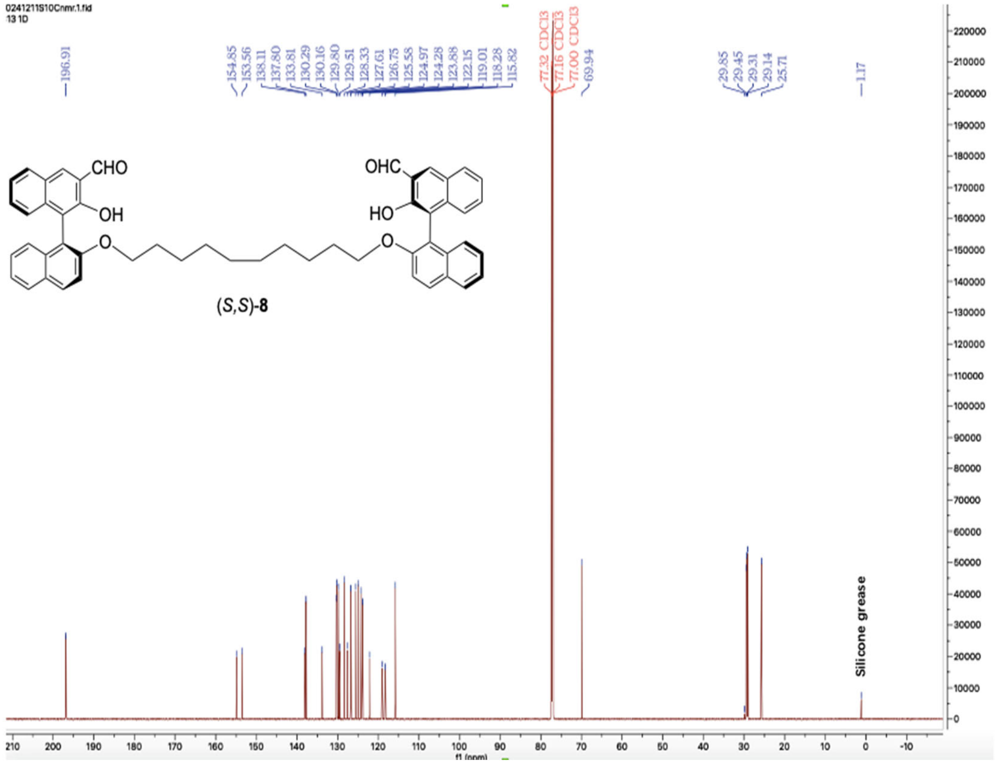

Figure S14. $^{13}C\{^{1}H\}$ NMR (150 MHz, $CDCl_3$) spectrum of (*S,S*)-**8** in $CDCl_3$.

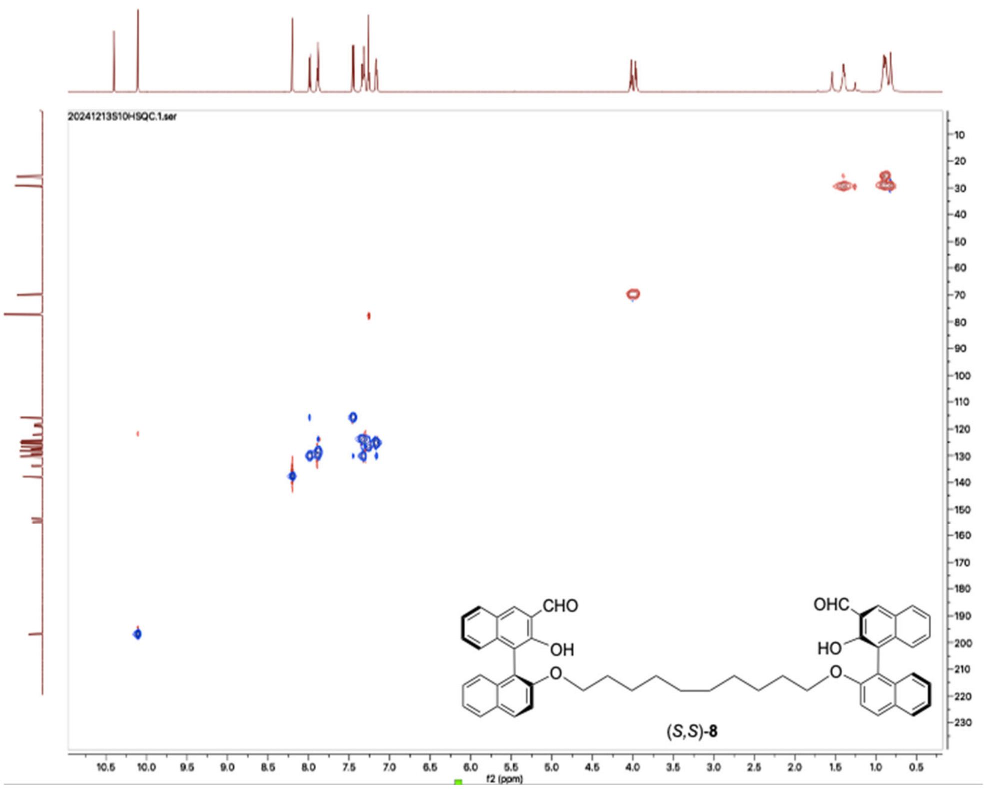

Figure S15. gHSQC NMR spectrum of (*S*,*S*)-**8** in $CDCl_3$. (600 MHz)

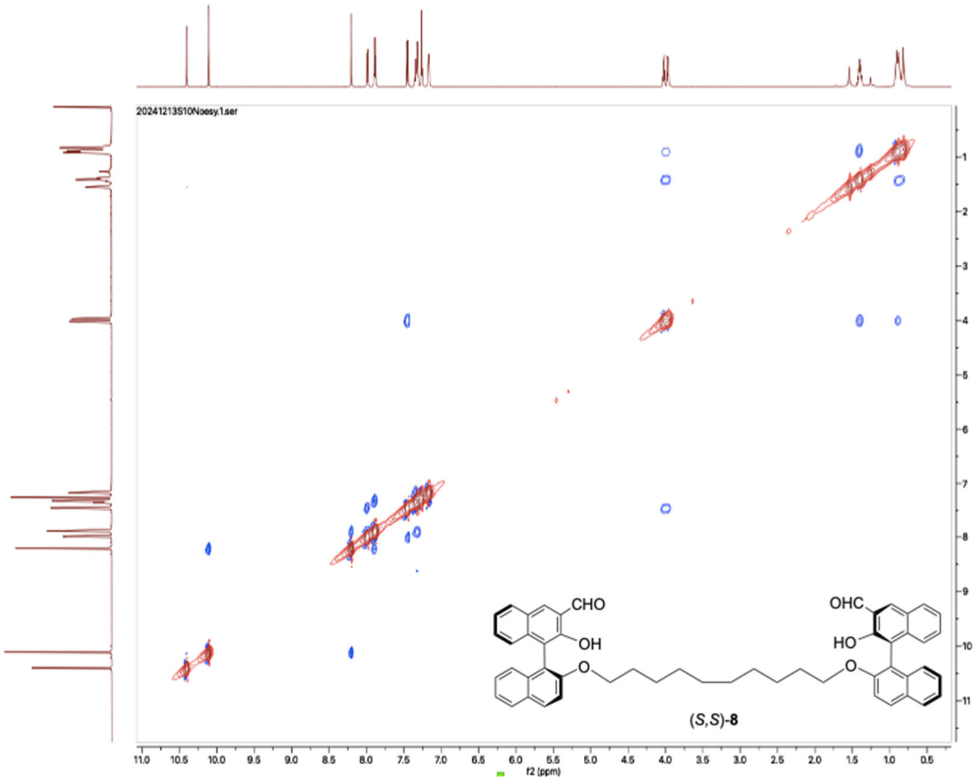

Figure S16. gNOESY spectrum of (*S*,*S*)-**8** in $CDCl_3$. (600 MHz)

HRMS of (*S*,*S*)-**8**

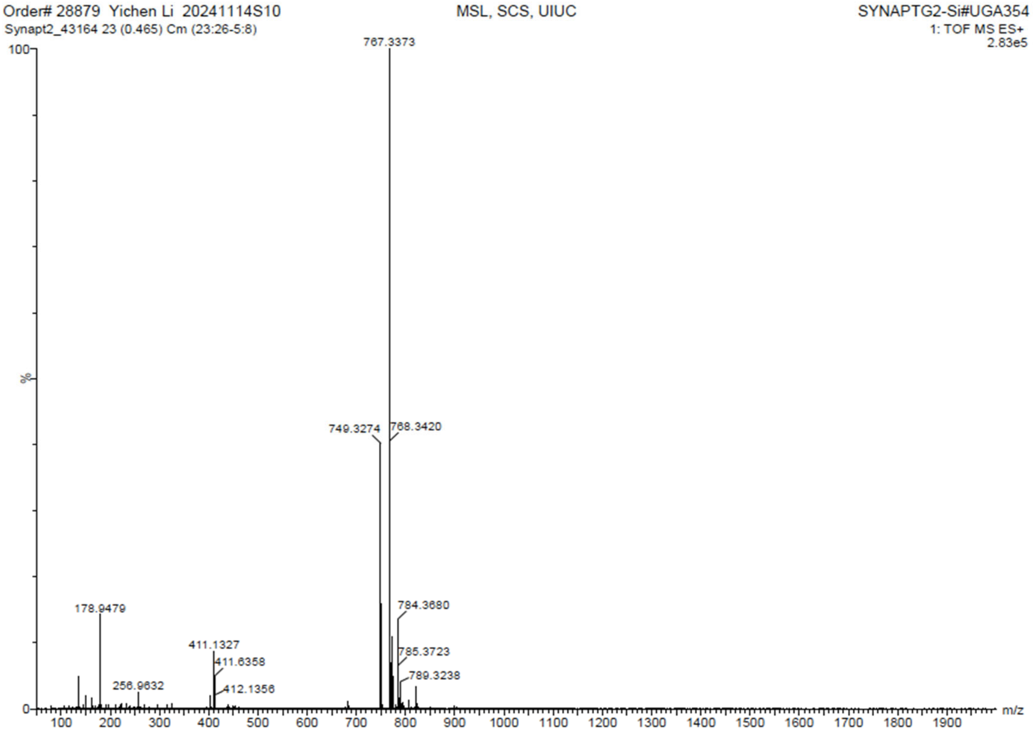

Elemental Composition Report

Page 1

Single Mass Analysis

Tolerance = 5.0 PPM / DBE: min = -1.5, max = 100.0
Element prediction: Off
Number of isotope peaks used for i-FIT = 8

Monoisotopic Mass, Even Electron Ions
336 formula(e) evaluated with 3 results within limits (up to 50 best isotopic matches for each mass)
Elements Used:
C: 0-70 H: 0-80 N: 0-6 O: 0-6

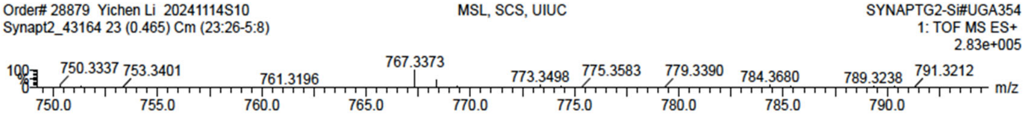

Mass	Calc. Mass	mDa	PPM	DBE	i-FIT	Norm	Conf(%)	Formula
Minimum:				-1.5				
Maximum:		5.0	5.0	100.0				
767.3373	767.3346	2.7	3.5	30.5	625.5	0.573	56.41	C48 H43 N6 O4
	767.3373	0.0	0.0	29.5	626.3	1.344	26.07	C52 H47 O6
	767.3386	-1.3	-1.7	34.5	626.7	1.742	17.52	C53 H43 N4 O2

Figure S17. HRMS of (*S*,*S*)-**8**

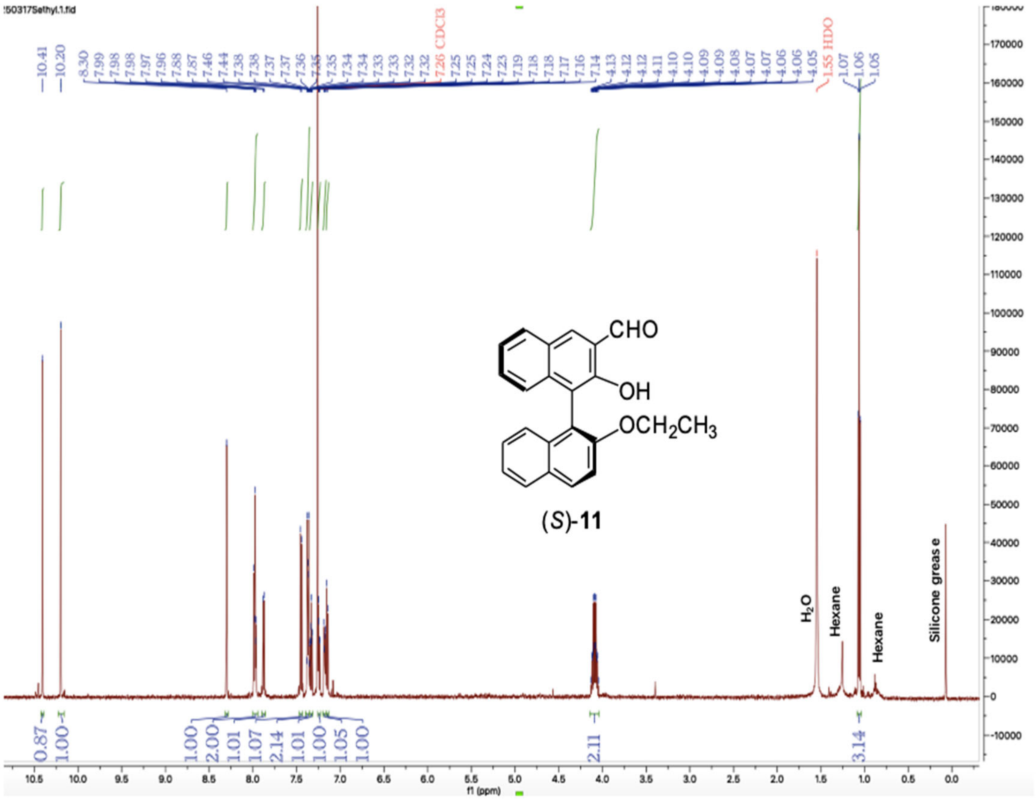

Figure S18. ^{1}H NMR spectrum of (*S*)-**11** in $CDCl_3$. (600 MHz)

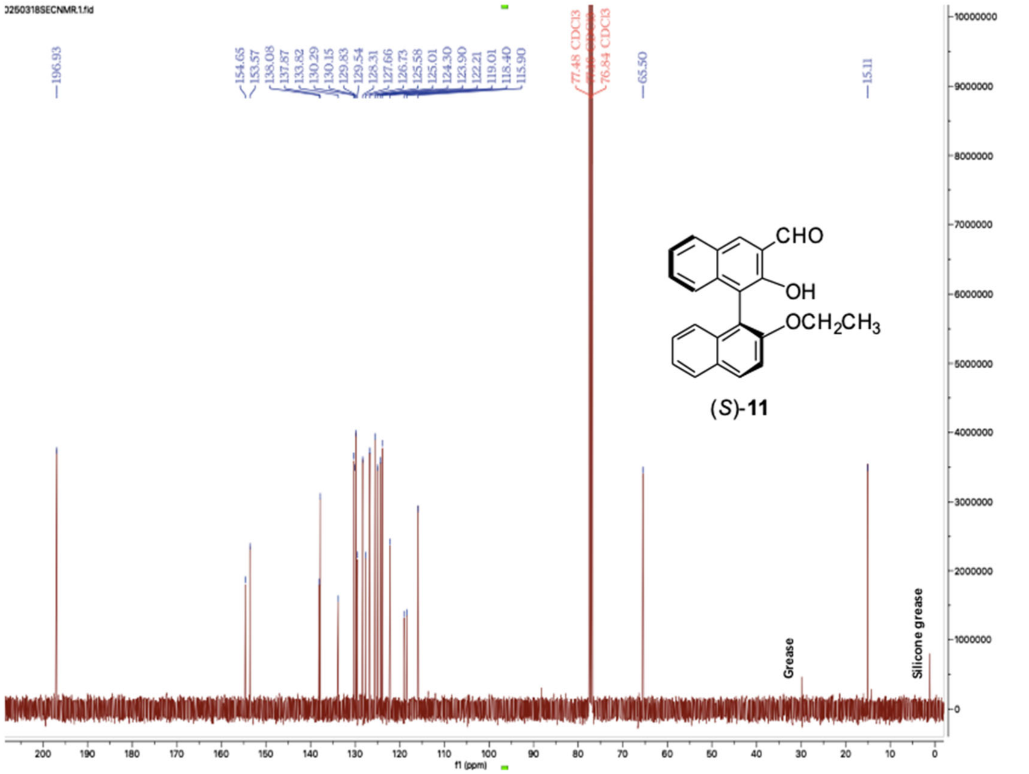

Figure S19. $^{13}C\{^{1}H\}$ NMR (150 MHz, $CDCl_3$) spectrum of (*S*)-**11** in $CDCl_3$.

2. NMR and Mass Spectral Studies on the Reaction of (*S,S*)-6 with Arginine in DMSO-d_6/17% D_2O

2.1. NMR and Mass Spectra of (*S,S*)-**6** with D-Arg

(1) ^1H NMR of (*S,S*)-**6** with 2 eq. D-Arg

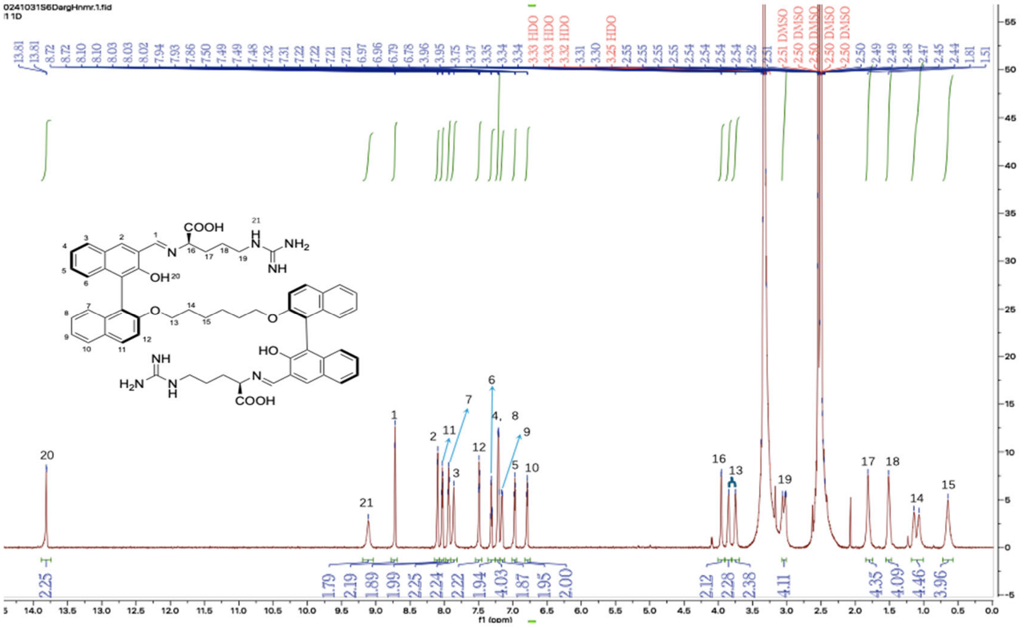

Figure S20. ^1H NMR of (*S,S*)-**6** with 2 eq. D-Arg in DMSO-d_6/17%D_2O. (600 MHz)

(2) $^{13}C\{^1H\}$ NMR of (*S,S*)-**6** with 2 eq. D-Arg

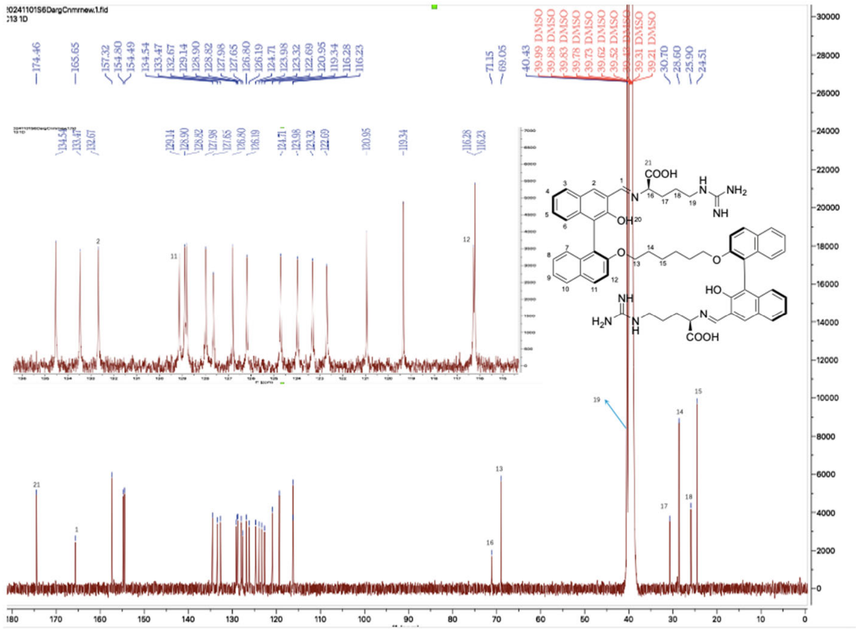

Figure S21. $^{13}C\{^1H\}$ NMR of (*S,S*)-**6** with 2 eq. D-Arg in DMSO-d_6/17%D_2O. (150 MHz)

(3) gHSQC NMR of (*S*,*S*)-**6** with 2 eq. D-Arg

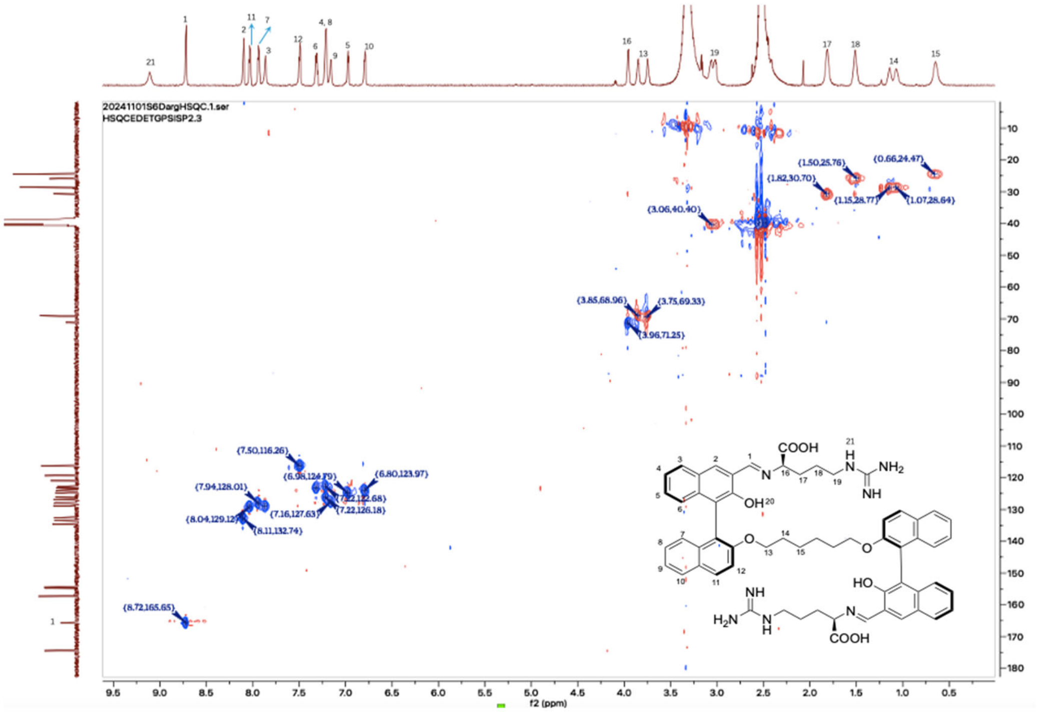

Figure S22. gHSQC of (*S*,*S*)-**6** with 2 eq. D-Arg in DMSO-d_6/17%D_2O. (600 MHz)

(4) gNOESY NMR of (*S*,*S*)-**6** with 2 eq. D-Arg

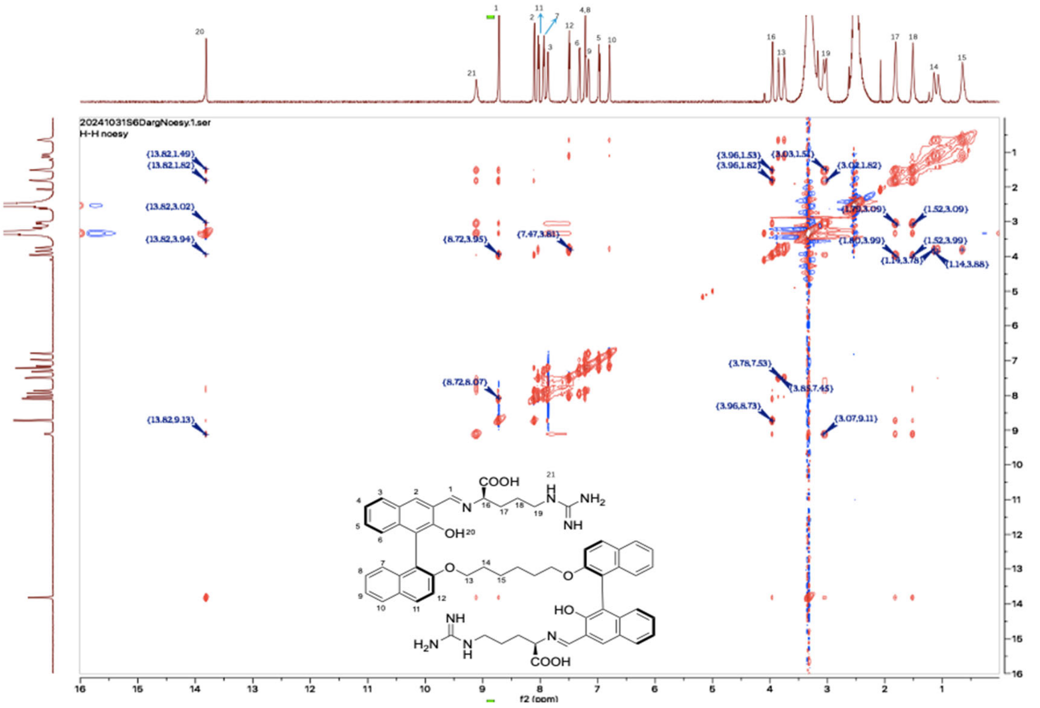

Figure S23. gNOESY of (*S*,*S*)-**6** with 2 eq. D-Arg in DMSO-d_6/17%D_2O. (600 MHz)

（5）gTOCSY NMR of (S,S)-**6** with 2 eq. D-Arg

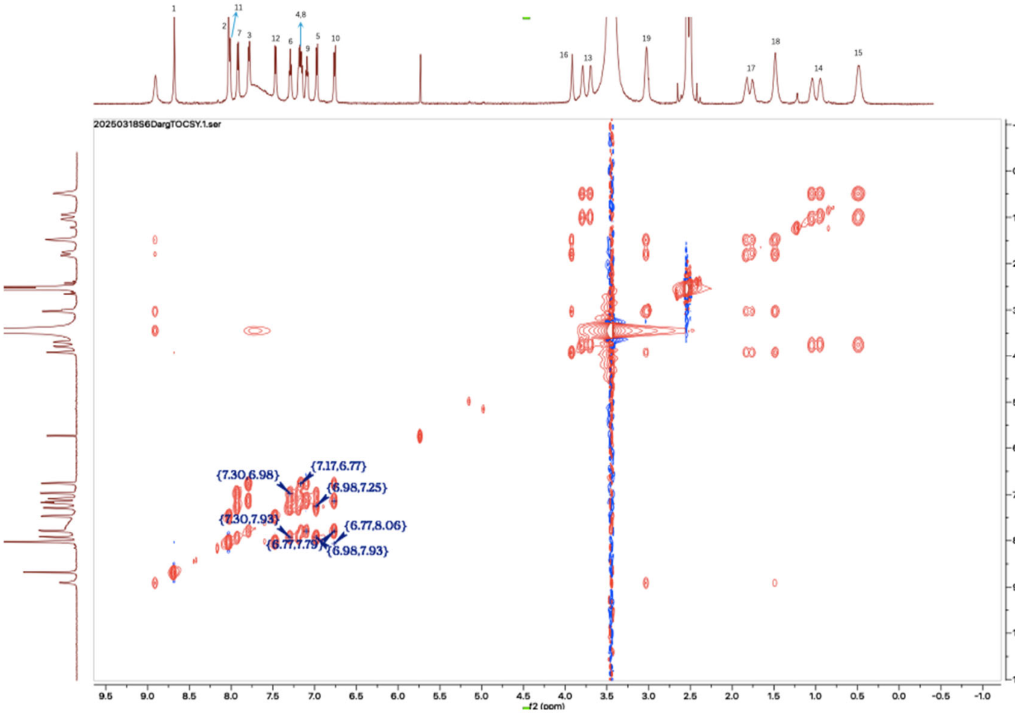

Figure S24. gTOCSY of (*S,S*)-**6** with 2 eq. D-Arg in DMSO-d_6/17%D_2O. (600 MHz)

(6) HRMS of (*S,S*)-**6** with 2 eq. D-Arg

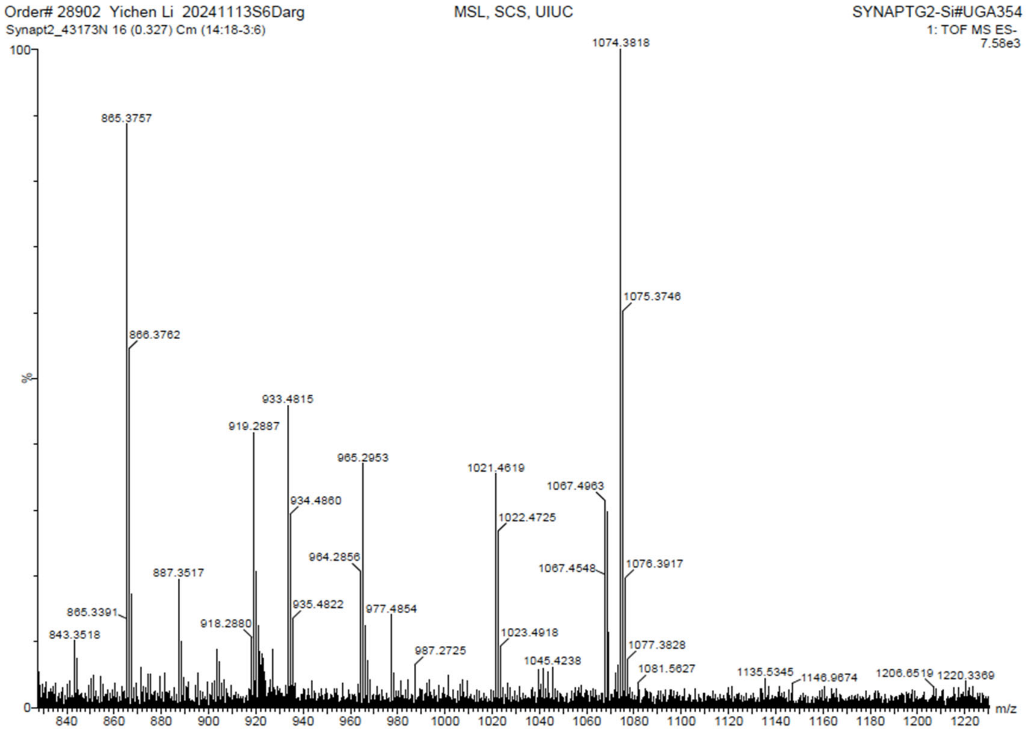

Elemental Composition Report **Page 1**

Single Mass Analysis
Tolerance = 5.0 PPM / DBE: min = -1.5, max = 100.0
Element prediction: Off
Number of isotope peaks used for i-FIT = 8

Monoisotopic Mass, Even Electron Ions
341 formula(e) evaluated with 2 results within limits (up to 50 best isotopic matches for each mass)
Elements Used:
C: 0-70 H: 0-80 N: 0-9 O: 0-9

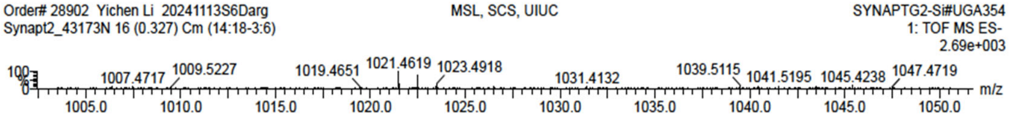

Minimum:				-1.5				
Maximum:		5.0	5.0	100.0				
Mass	Calc. Mass	mDa	PPM	DBE	i-FIT	Norm	Conf(%)	Formula
1021.4619	1021.4653	-3.4	-3.3	38.5	740.7	0.557	57.32	C65 H61 N6 O6
	1021.4612	0.7	0.7	34.5	741.0	0.851	42.68	C60 H61 N8 O8

Figure S25. HRMS of (*S,S*)-**6** with 2 eq. D-Arg

A signal at m/z = 1021.4610 for **9**$_D$-H (Calcd for $C_{60}H_{61}N_8O_8$: 1021.4612).

A signal at m/z = 1074.3818 for **9**$_D$+3Na-NH$_3$ (Calcd for $C_{60}H_{59}N_7Na_3O_8$: 1074.4118).

A signal at m/z = 1067.4548 for **9**$_D$+2Na-H (Calcd for $C_{60}H_{61}N_8Na_2O_8$: 1067.4408).

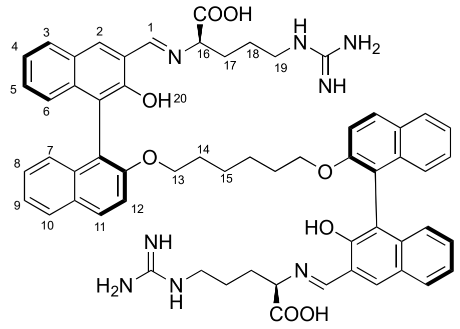

Compound $\mathbf{9_D}$

Assignment for the ^{1}H NMR signals of compound $\mathbf{9_D}$: The down-field imine proton 1 shows a NOESY correlation with the aromatic singlet proton 2 and α-aliphatic proton 16 of the arginine unit. Proton 2 shows a NOESY correlation with the aromatic proton 3. Proton 3 shows a cross peak with proton 4 and proton 4 shows a cross peak with proton 5 in NOESY. Aliphatic protons 13 shows an NOE effect with the aromatic proton 12, and proton 12 shows a cross peak with proton 11 in NOESY. Proton 11 shows a cross peak with proton 10 in TOCSY. In NOESY, proton 10 shows a cross peak with proton 9. Proton 9 shows a cross peak with proton 8 in NOESY. Proton 8 shows a cross peak with proton 7 and proton 7 shows a cross peak with proton 6 in NOESY.

2.2. NMR and Mass Spectra of (*S,S*)-**6** with L-Arg

（1）^{1}H NMR spectrum of (*S,S*)-**6** with 2 eq. L-Arg

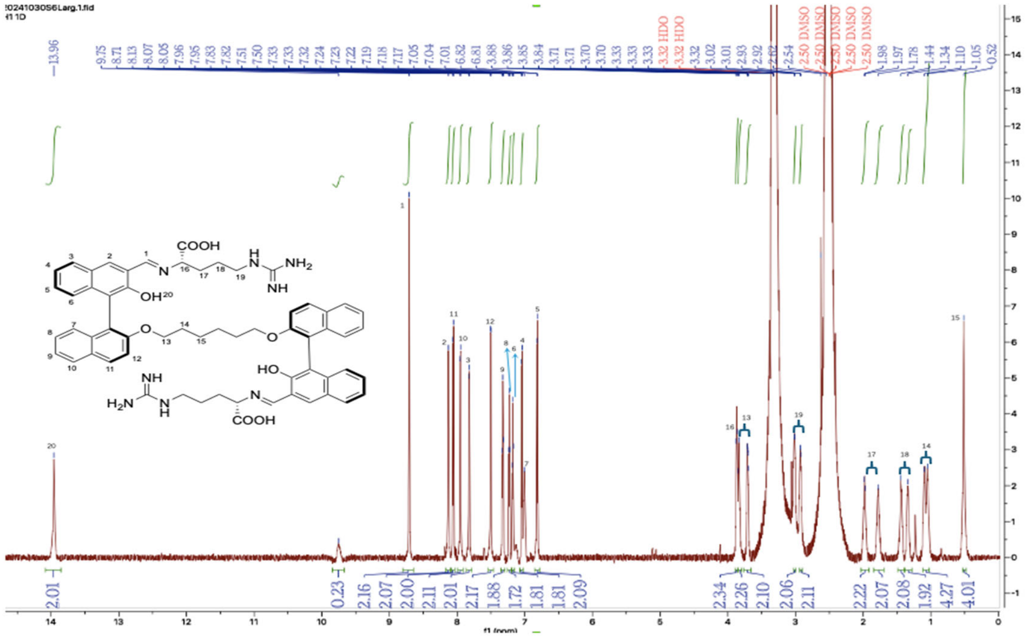

Figure S26. ^{1}H NMR of (*S,S*)-**6** with 2 eq. L-Arg in DMSO-d_6/17%D_2O. (600 MHz)

（2）$^{13}C\{^{1}H\}$ NMR of (*S,S*)-**6** with 2 eq. L-Arg

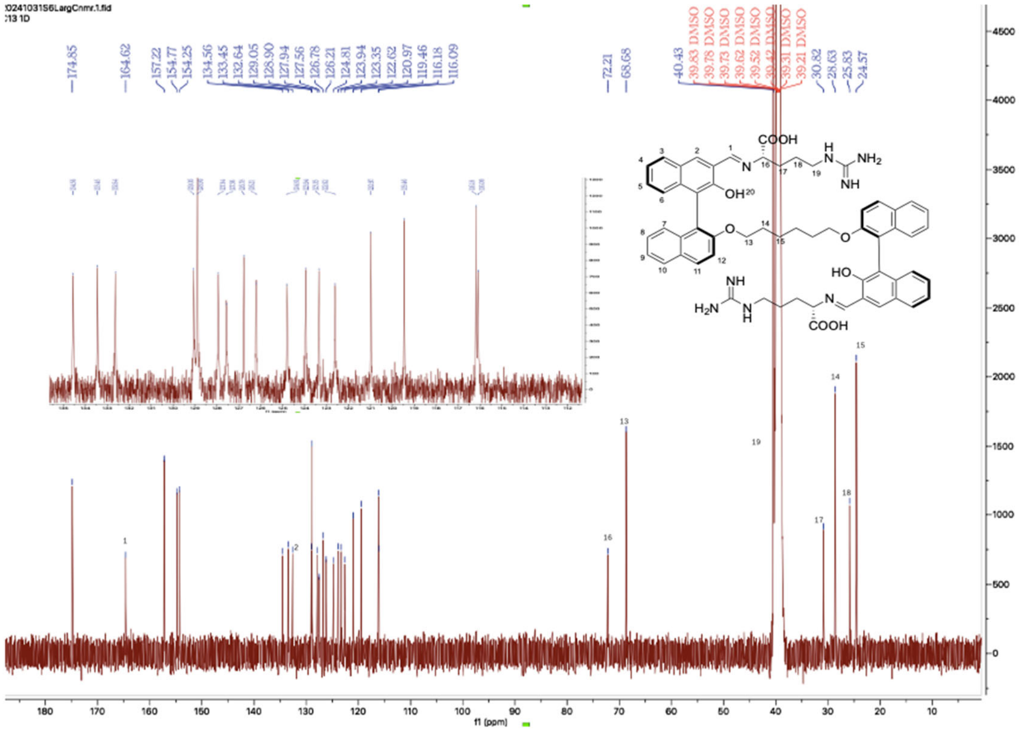

Figure S27. $^{13}C\{^{1}H\}$ NMR of (*S,S*)-**6** with 2 eq. L-Arg in DMSO-d_6/17%D_2O. (150 MHz)

（3）gHSQC NMR of (*S,S*)-**6** with 2 eq. L-Arg

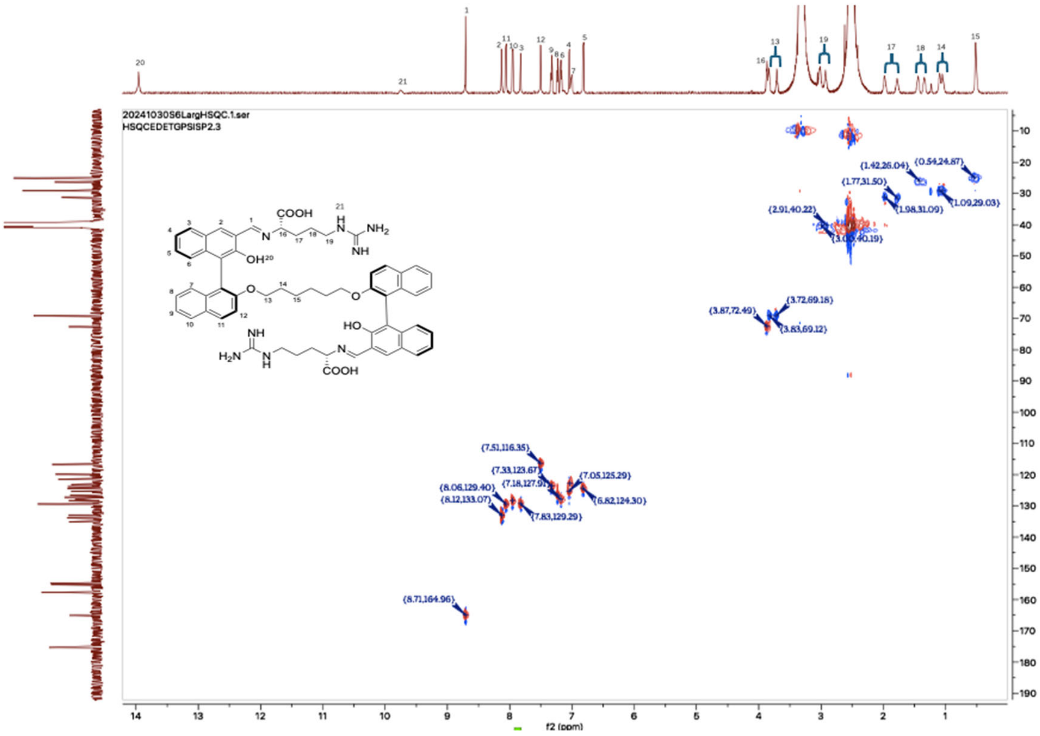

Figure S28. gHSQC of (*S,S*)-**6** with 2 eq. L-Arg in DMSO-d$_6$/17%D$_2$O. (600 MHz)

（4）gNOESY NMR of (*S,S*)-**6** with 2 eq. L-Arg

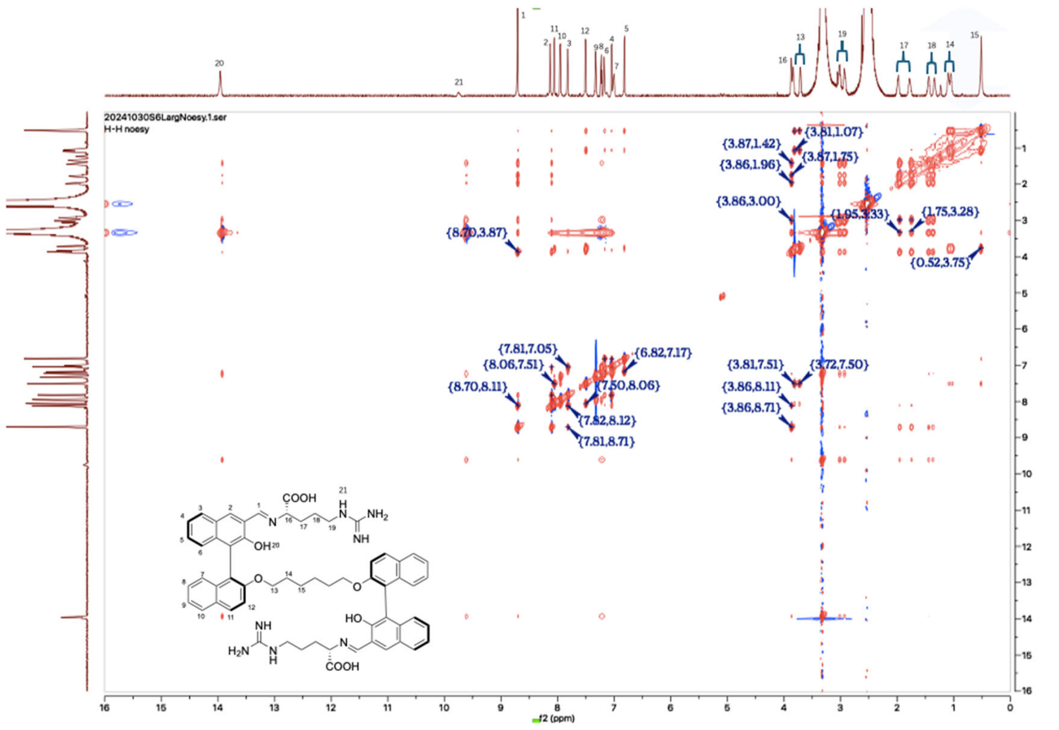

Figure S29. gNOESY of (*S,S*)-**6** with 2 eq. L-Arg in DMSO-d$_6$/17%D$_2$O. (600 MHz)

（5）HRMS of (*S,S*)-**6** with 2 eq. L-Arg

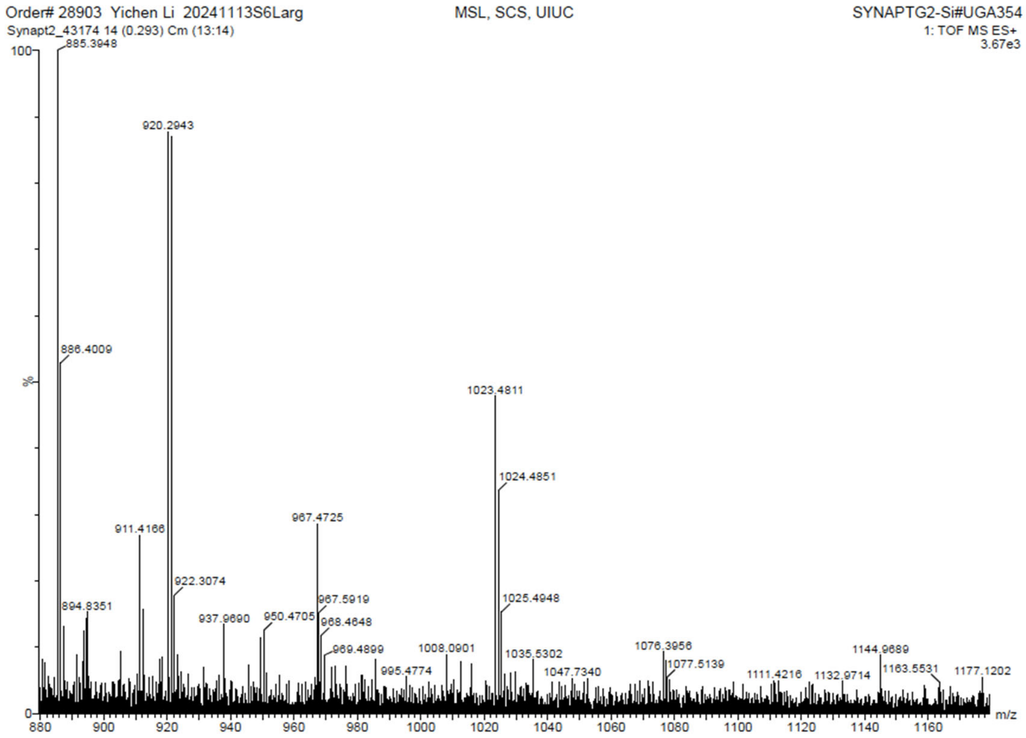

Elemental Composition Report **Page 1**

Single Mass Analysis
Tolerance = 5.0 PPM / DBE: min = -1.5, max = 100.0
Element prediction: Off
Number of isotope peaks used for i-FIT = 8

Monoisotopic Mass, Even Electron Ions
333 formula(e) evaluated with 4 results within limits (up to 50 best isotopic matches for each mass)
Elements Used:
C: 0-70 H: 0-80 N: 0-9 O: 0-9

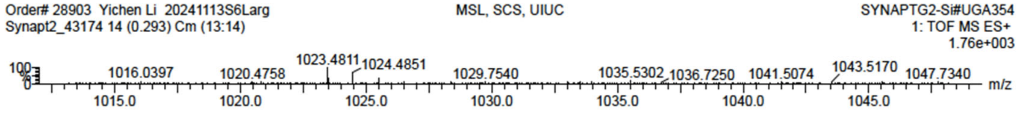

Minimum:				-1.5				
Maximum:		5.0	5.0	100.0				

Mass	Calc. Mass	mDa	PPM	DBE	i-FIT	Norm	Conf(%)	Formula
1023.4811	1023.4836	-2.5	-2.4	36.5	887.6	0.964	38.15	C69 H67 O8
	1023.4849	-3.8	-3.7	41.5	887.7	1.049	35.02	C70 H63 N4 O4
	1023.4809	0.2	0.2	37.5	888.3	1.645	19.30	C65 H63 N6 O6
	1023.4769	4.2	4.1	33.5	889.2	2.586	7.53	C60 H63 N8 O8

Figure S30. HRMS of (*S,S*)-**6** with 2 eq. L-Arg

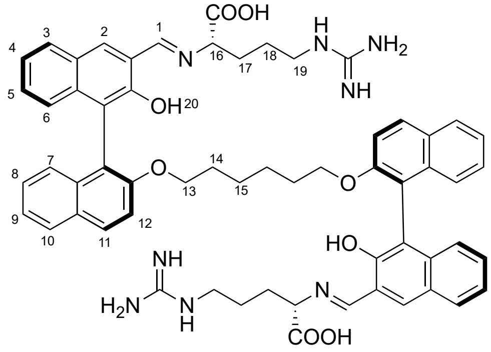

Compound $\mathbf{9_L}$

Assignment for the ^{1}H NMR signals of compound $\mathbf{9_L}$: The down-field imine proton 1 shows a NOESY correlation with the aromatic singlet proton 2 and the aliphatic proton 16. Proton 2 shows a NOESY correlation with the aromatic proton 3. Proton 3 shows a cross peak with proton 4 and proton 4 shows a cross peak with proton 5 in NOESY. The aliphatic protons 13 shows an NOE effect with the aromatic proton 12, and proton 12 shows a cross peak with proton 11. In NOESY, proton 10 shows a cross peak with proton 9. Proton 9 shows a cross peak with proton 8. Proton 8 shows a cross peak with proton 7 and proton 7 shows a cross peak with proton 6 in NOESY.

3. NMR and Mass Spectral Studies on the Reaction of (*S,S*)-6 with Arginine and $Zn(OAc)_2$ in DMSO-d_6/14% D_2O

3.1. NMR and mass spectra of (*S,S*)-**6** + 2 eq. D-Arg with 1 eq. $Zn(OAc)_2$

(1) ^{1}H NMR of (*S,S*)-**6** + 2 eq. D-Arg with 1 eq. $Zn(OAc)_2$

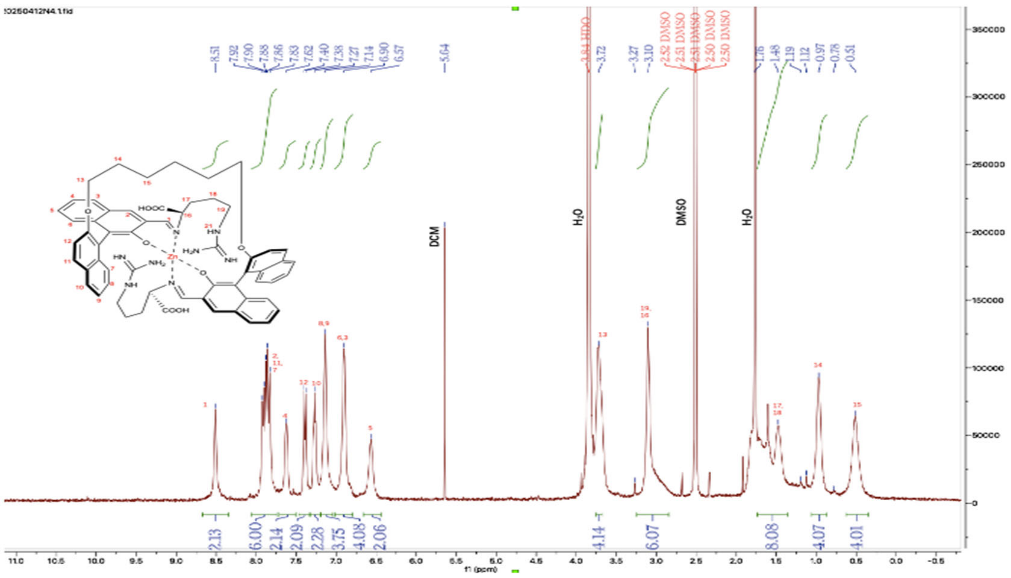

Figure S31. ^{1}H NMR spectrum of (*S,S*)-**6** + 2 eq. D-Arg with 1 eq. $Zn(OAc)_2$ in DMSO-d_6/14%D_2O. (600 MHz)

(2) $^{13}C\{^{1}H\}$ NMR of (*S,S*)-**6** + 2 eq. D-Arg with 1 eq. $Zn(OAc)_2$

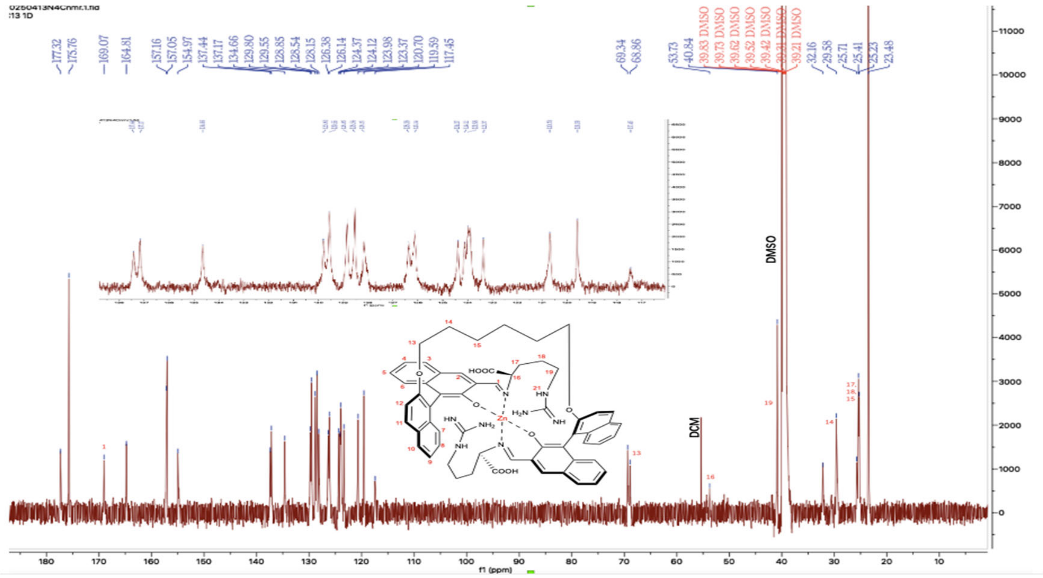

Figure S32. $^{13}C\{^{1}H\}$ NMR of (*S,S*)-**6** + 2 eq. D-Arg with 1 eq. $Zn(OAc)_2$ in DMSO-d_6/14%D_2O. (150 MHz)

（3）gHSQC NMR of (*S*,*S*)-**6** + 2 eq. D-Arg with 1 eq. $Zn(OAc)_2$

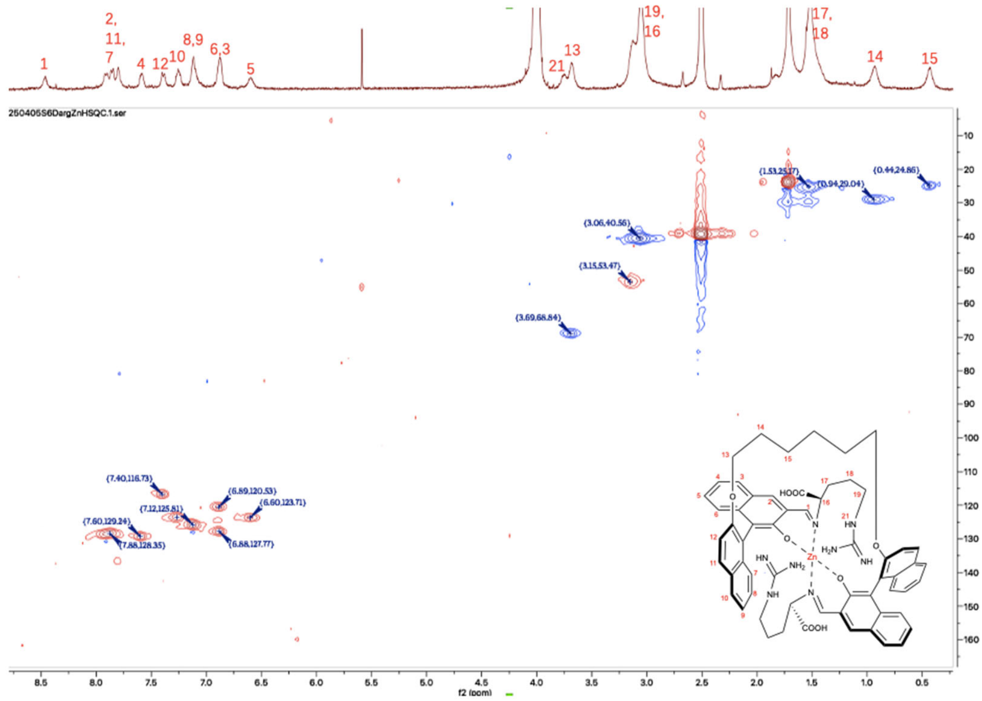

Figure S33. (a) gHSQC of (*S*,*S*)-**6** + 2 eq. D-Arg with 1 eq. $Zn(OAc)_2$ in DMSO-d_6/14%D_2O. (600 MHz)

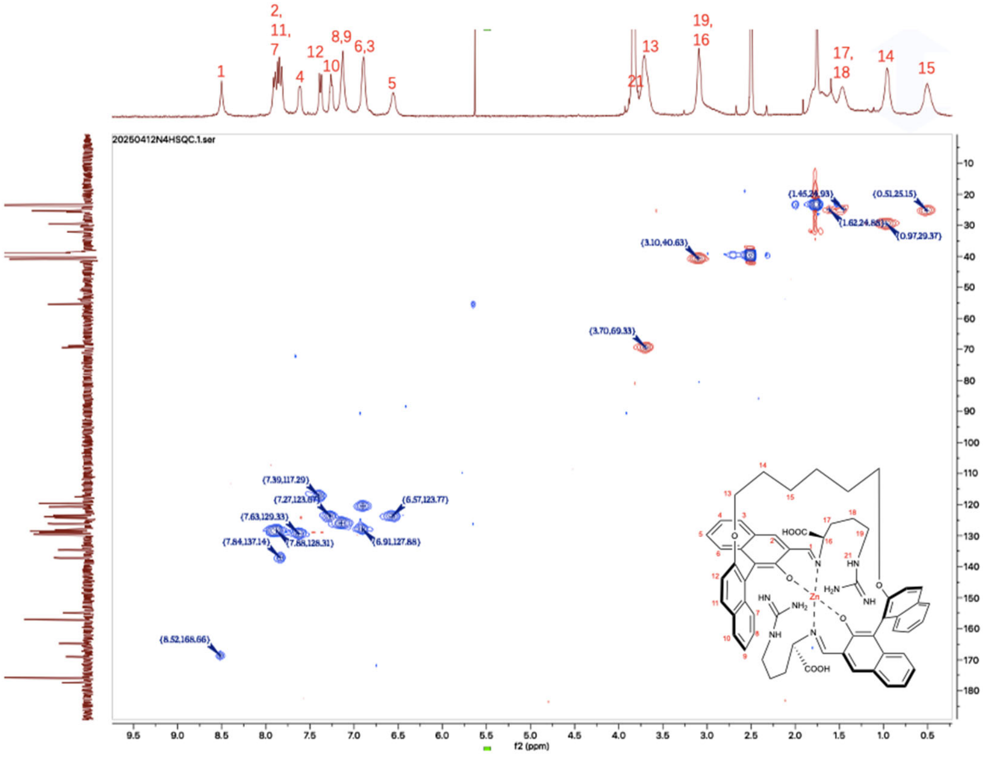

Figure S33. (b) gHSQC of (*S*,*S*)-**6** + 2 eq. D-Arg with 1 eq. $Zn(OAc)_2$ in DMSO-d_6/14%D_2O. (600 MHz)

In Fig. S33 (a), the cross peak (3.15, 53.47) corresponds to the chiral methine CH group (16) next to the imine. In Fig. S33 (b), the cross peak (8.52, 168.66) corresponds to the imine CH (1). Different instruments had to be used to collect these two spectra to get clear proton signals of the imine group (1) and the chiral α-CH (16) of the arginine unit.

Bruker Avance III 800 (4 RF Channels) was used to obtain Fig. S33 (b). 800 MHz field strength (18.8 Tesla). The default coupling constant of the instrument for the proton-carbon is 145 Hz. D1(delays) = 2 sec; Aq (Acquisition Time) (F2,F1): 0.0799, 0.00265.

Bruker Neo 400 MHz (2 RF Channels) was used to obtain Fig. S33 (a). 400 MHz field strength (9.4 Tesla). The default coupling constant of the instrument for the proton-carbon is 145 Hz. D1(delays) = 1.5 Sec; Aq (Acquisition Time) (F2, F1): 0.0819, 0.0798.

In Fig. S33 (a), there is no cross peak for H-21 which indicate that this is an amine proton not bonded to a carbon. In the NOESY spectrum (Fig. S34), H-21 has cross peaks with H-1, H-16, H-17, H-18, H-19 which indicate the spatial adjacency and give evidence of the guanidine group coordination with the zinc ion. After coordination, it formed a cycle and showed the cross peaks between each other in the NOESY spectrum.

(4) gNOESY NMR of (*S,S*)-**6** with 2 eq. D-Arg by adding 1 eq. $Zn(OAc)_2$

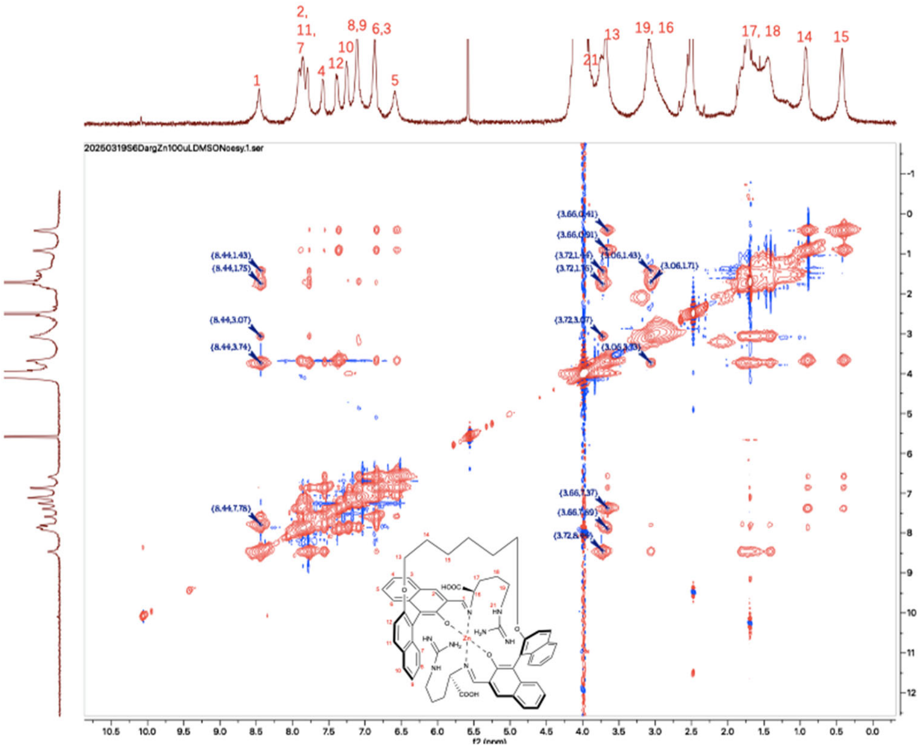

Figure S34. gNOESY of (*S,S*)-**6** + 2 eq. D-Arg with 1 eq. $Zn(OAc)_2$ in DMSO-d_6/14%D_2O. (600 MHz)

(5) gTOCSY NMR of (*S,S*)-**6** + 2 eq. D-Arg with 1 eq. $Zn(OAc)_2$

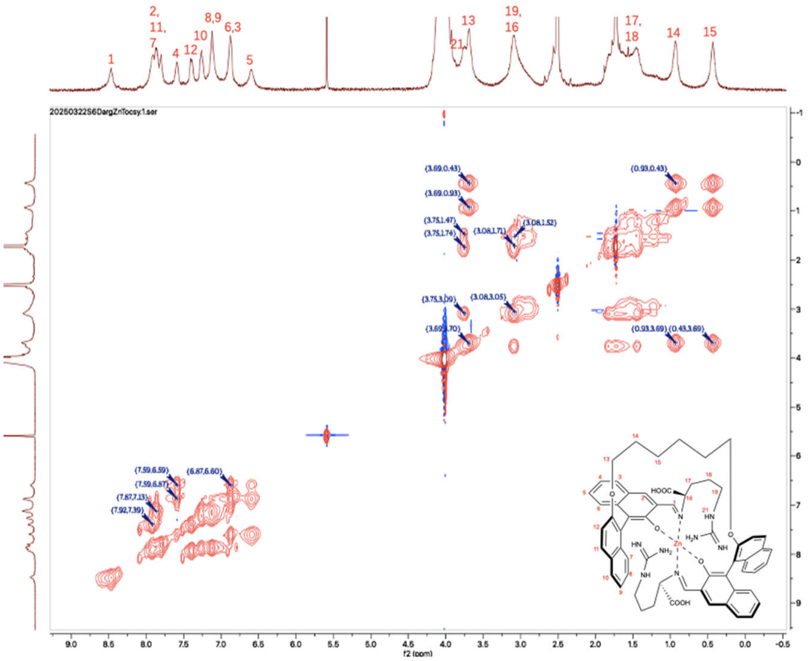

Figure S35. gTOCSY of (*S,S*)-**6** + 2 eq. D-Arg with 1 eq. $Zn(OAc)_2$ in DMSO-d_6/14%D_2O. (600 MHz)

（6）HRMS of (*S,S*)-**6** + 2 eq. D-Arg with 1 eq. $Zn(OAc)_2$

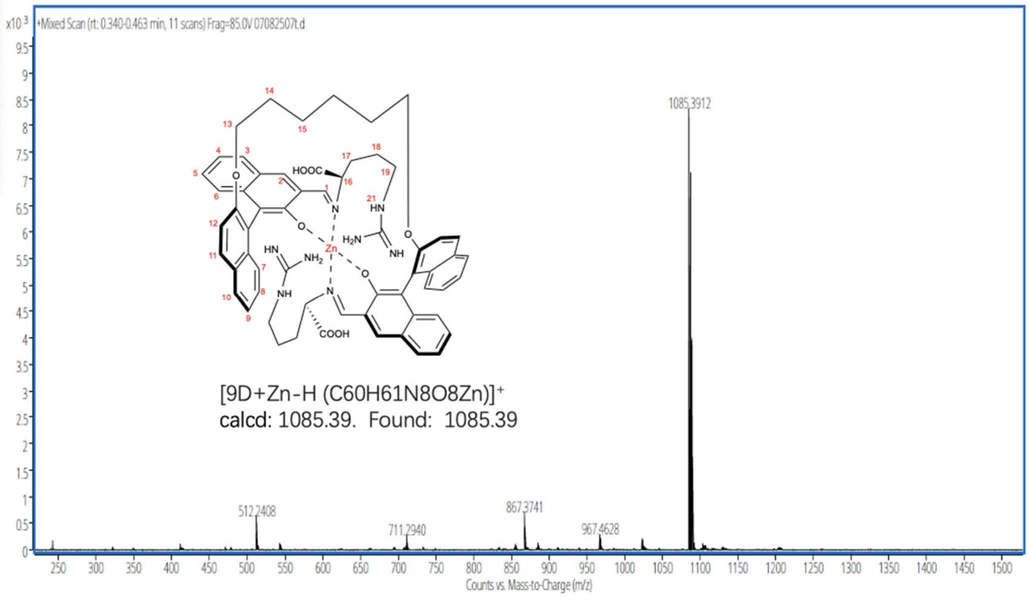

Figure S36. HRMS of (*S,S*)-**6** + 2 eq. D-Arg with 1 eq. $Zn(OAc)_2$ in DMSO-d_6/14%D_2O).

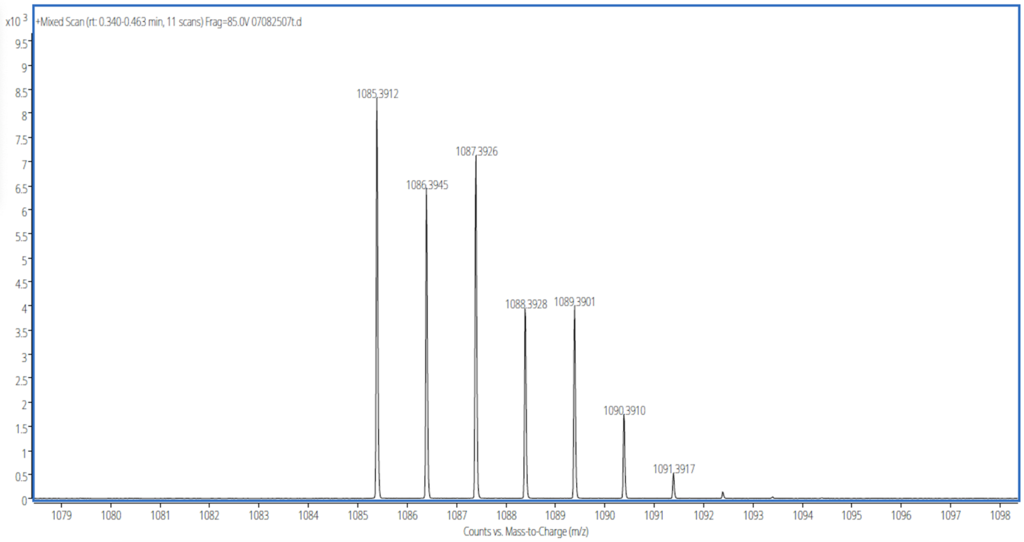

Figure S37. HRMS of (*S,S*)-**6** + 2 eq. D-Arg with 1 eq. $Zn(OAc)_2$ in DMSO-d_6/14%D_2O).

High-resolution mass spectra were obtained from the University of California Riverside Mass Spectrometry Facility (method ESI+, direct infusion).

3.2. NMR and Mass Spectra of (*S,S*)-**6** + 2 eq. L-Arg with 1 eq. $Zn(OAc)_2$

(1) ^1H NMR of (*S,S*)-**6** + 2 eq. L-Arg with 1 eq. $Zn(OAc)_2$

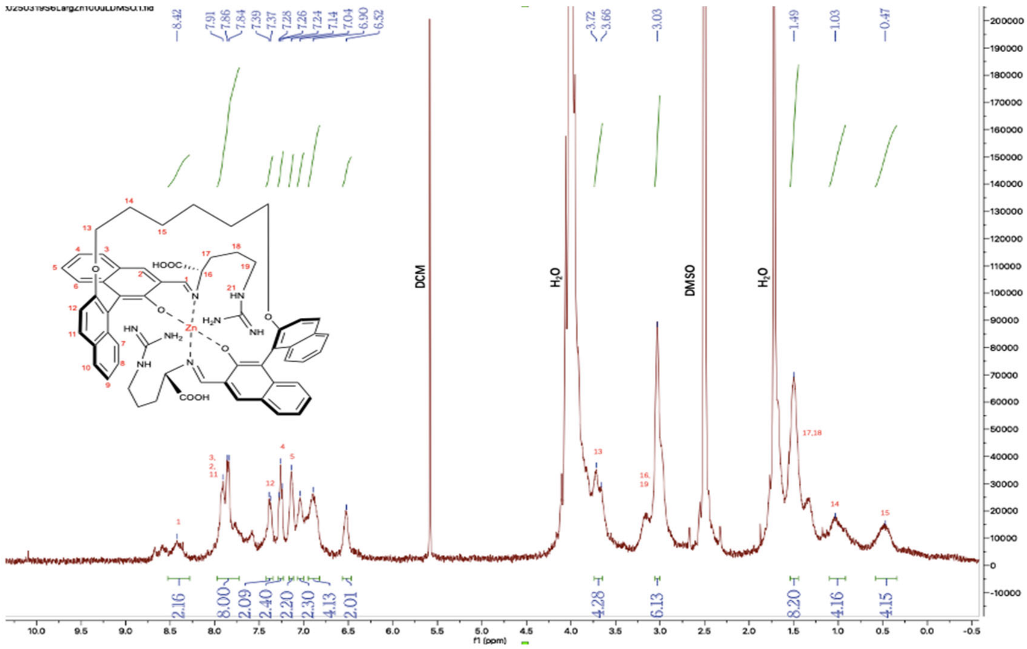

Figure S38. ^1H NMR of (*S,S*)-**6** + 2 eq. L-Arg with 1 eq. $Zn(OAc)_2$ in DMSO-d_6/14%D_2O. (600 MHz)

(2) $^{13}C\{^1H\}$ NMR of (*S,S*)-**6** + 2 eq. L-Arg with 1 eq. $Zn(OAc)_2$

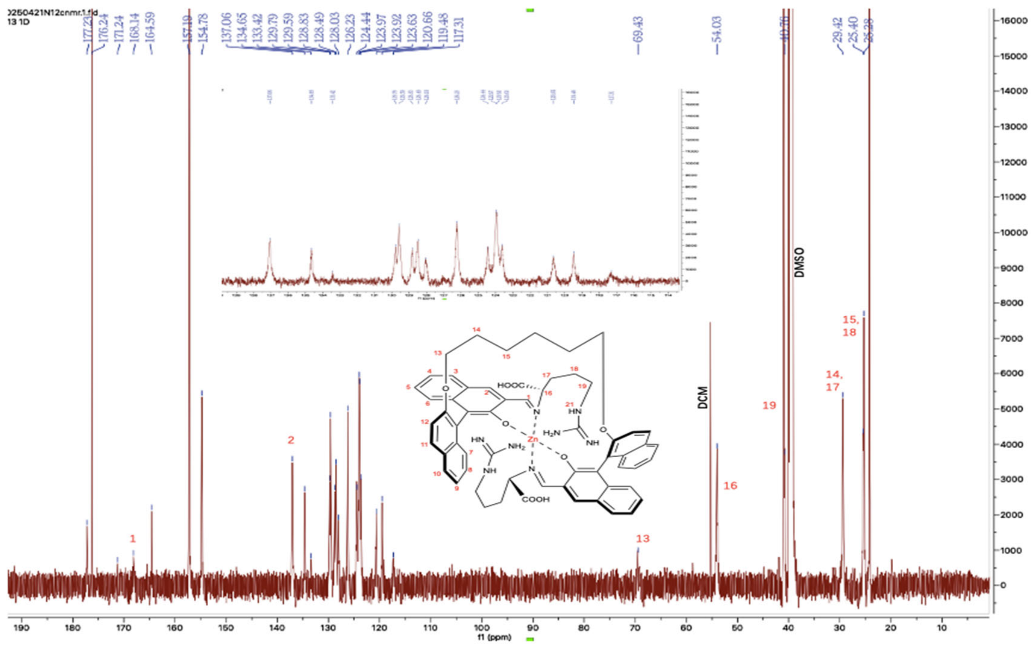

Figure S39. $^{13}C\{^1H\}$ NMR of (*S,S*)-**6** + 2 eq. L-Arg with 1 eq. $Zn(OAc)_2$ in DMSO-d_6/14%D_2O. (150 MHz)

（3）gHSQC NMR of (*S*,*S*)-**6** + 2 eq. L-Arg with 1 eq. $Zn(OAc)_2$

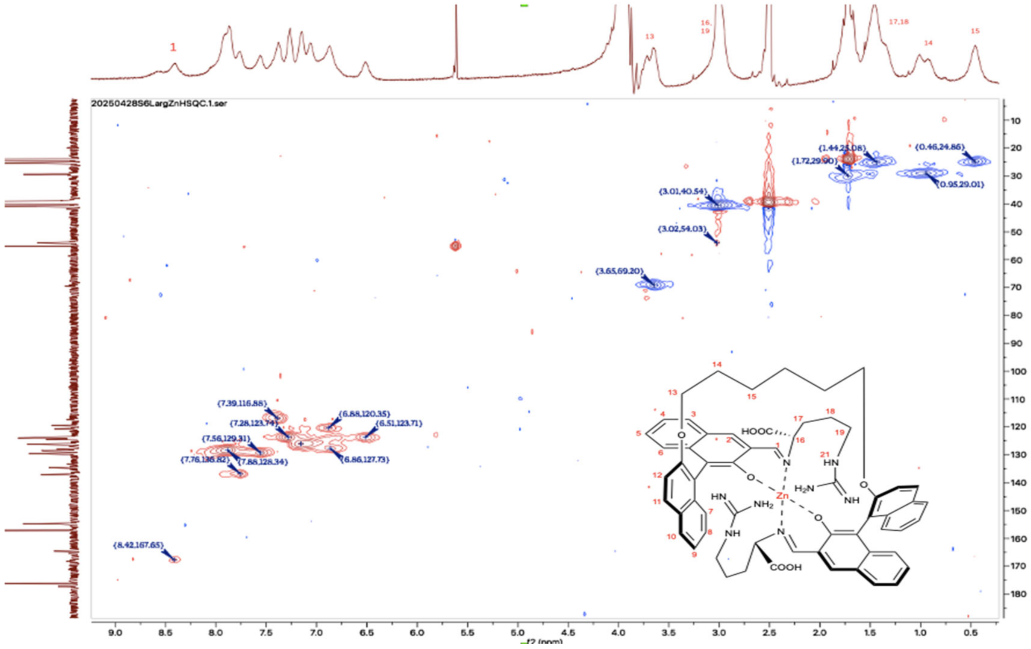

Figure S40. gHSQC of (*S*,*S*)-**6** + 2 eq. L-Arg with 1 eq. $Zn(OAc)_2$ in DMSO-d_6/14%D_2O. (600 MHz)

（4）gNOESY Spectrum of (*S*,*S*)-**6** + 2 eq. L-Arg with 1 eq. $Zn(OAc)_2$

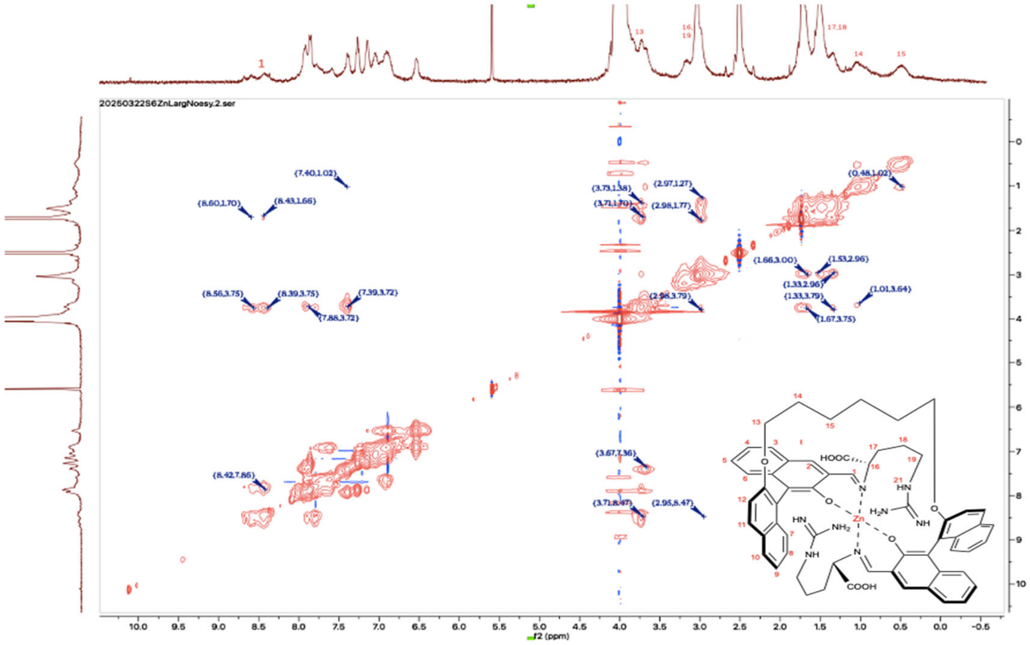

Figure S41. gNOESY of (*S*,*S*)-**6** + 2 eq. L-Arg with 1 eq. $Zn(OAc)_2$ in DMSO-d_6/14%D_2O. (600 MHz)

(5) gTOCSY Spectrum of (*S,S*)-**6** + 2 eq. L-Arg with 1 eq. $Zn(OAc)_2$

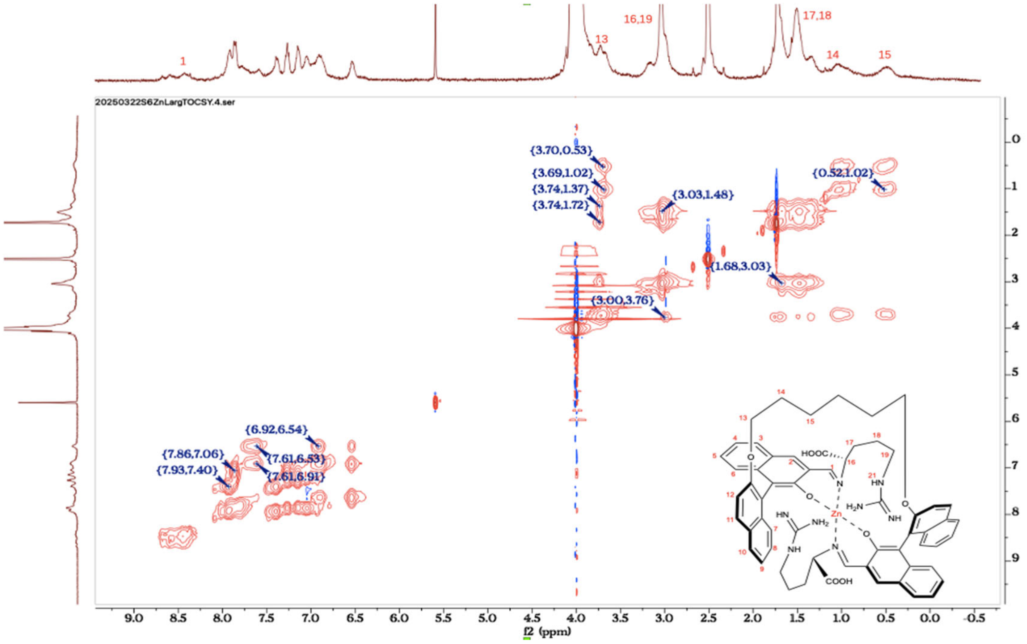

Figure S42. gTOCSY of (*S,S*)-**6** + 2 eq. L-Arg with 1 eq. $Zn(OAc)_2$ in DMSO-d_6/14%D_2O. (600 MHz)

(6) HRMS of (*S,S*)-**6** + 2 eq. L-Arg with 1 eq. $Zn(OAc)_2$

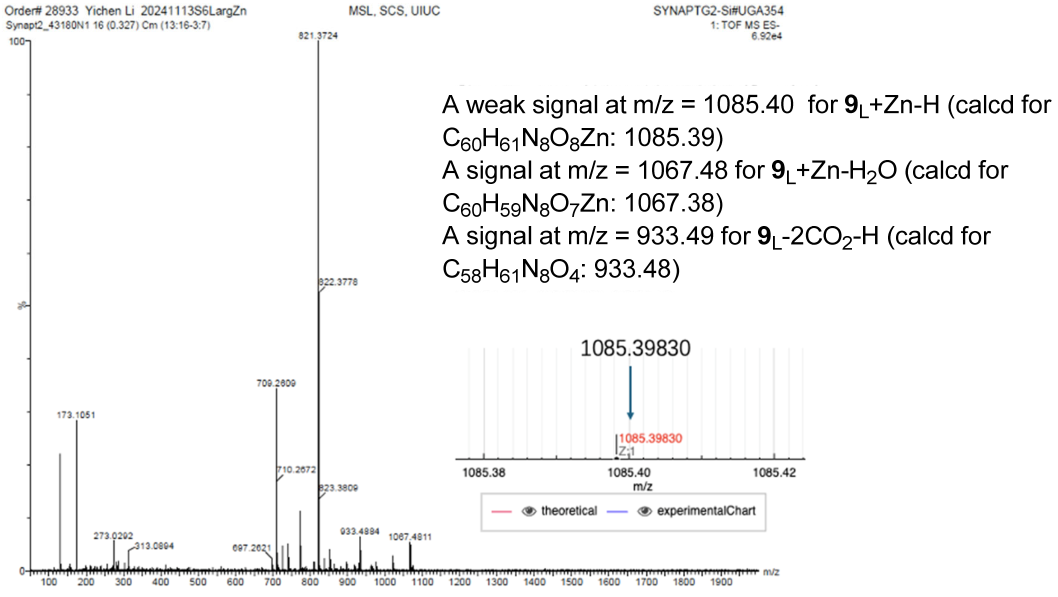

Figure S43. HRMS of (*S,S*)-**6** + 2 eq. L-Arg with 1 eq. $Zn(OAc)_2$ in DMSO-d_6/14%D_2O. The high-resolution mass spectra were obtained from University of Illinois at Urbana-Champaign (UIUC) Mass Spectrometry Facility (method ESI-, direct infusion).

3.3. NMR and Mass Spectra of (*S,S*)-**6** + 2 eq. L-Arg with 1 eq. $Zn(OAc)_2$

(1) ^{1}H NMR spectrum of (*S,S*)-**6** in DMSO-d_6/14%D_2O

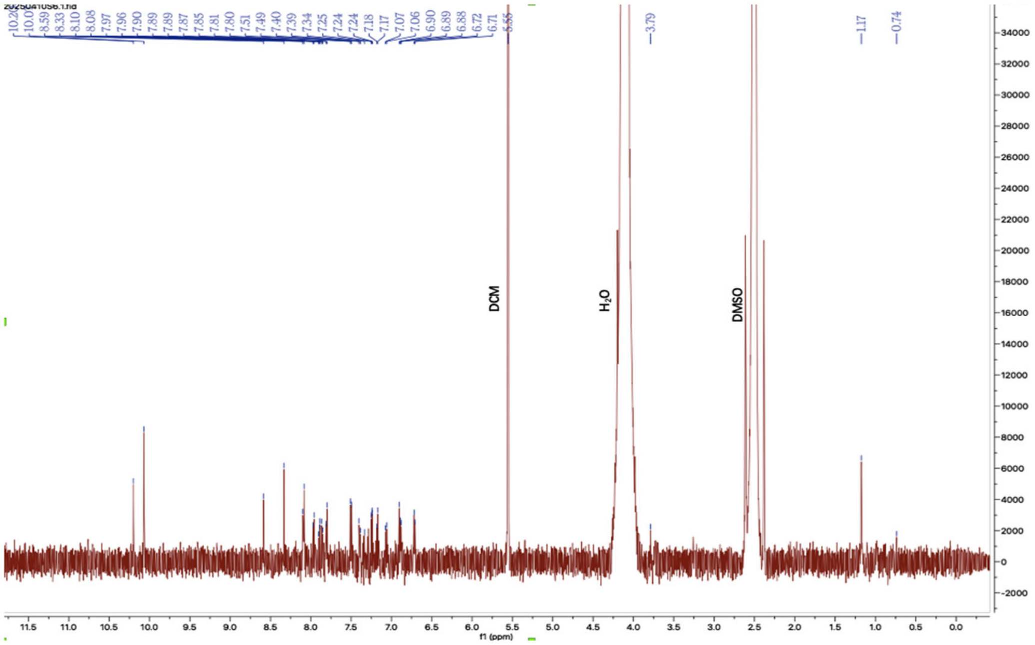

Figure S44. ^{1}H NMR of (*S,S*)-**6** in DMSO-d_6/14%D_2O. (600 MHz)

(2) ^{1}H and $^{13}C\{^{1}H\}$ NMR Spectra of D-Arg in DMSO-d_6/14%D_2O

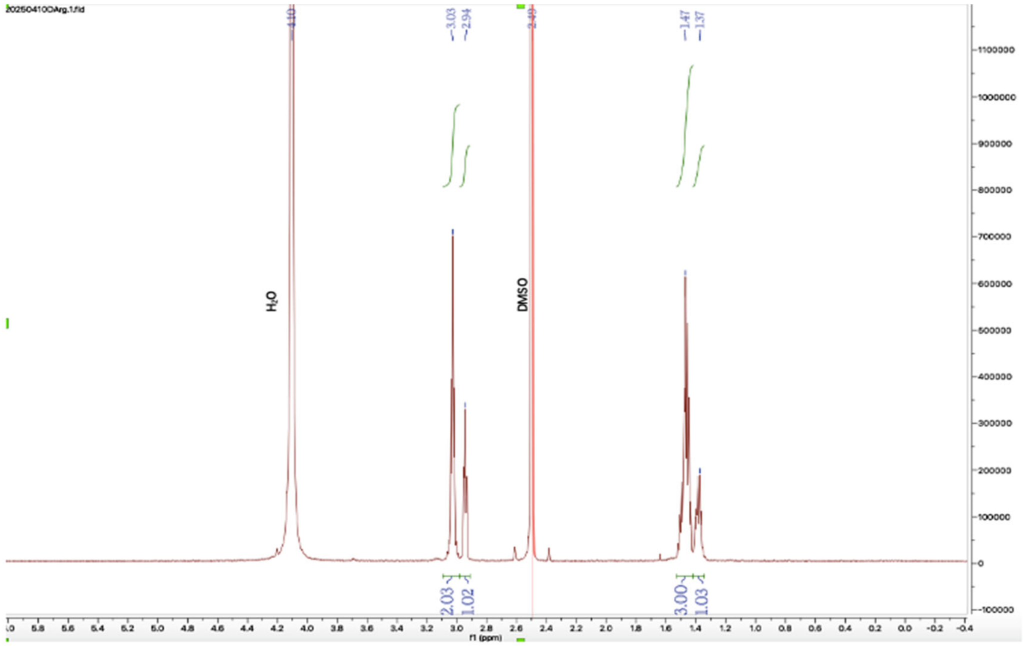

Figure S45. ^{1}H NMR of D-Arg in DMSO-d_6/14%D_2O. (600 MHz)

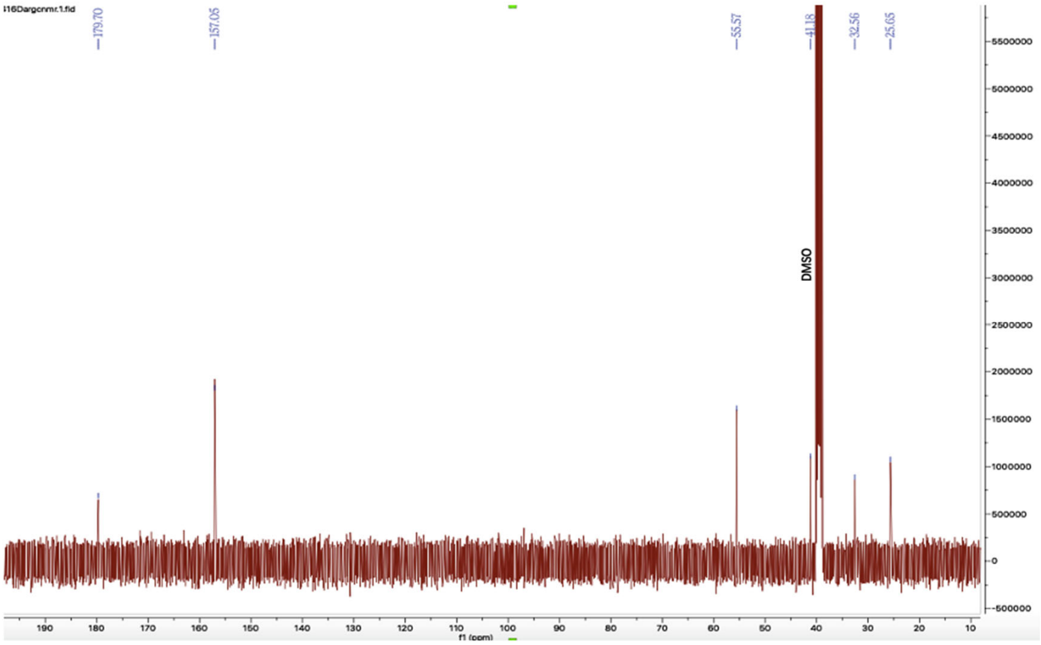

Figure S46. $^{13}C\{^{1}H\}$ NMR of D-Arg in DMSO-d_6/14%D_2O. (150 MHz)

(3) ^{1}H NMR of L-Arg in DMSO-d_6/14%D_2O

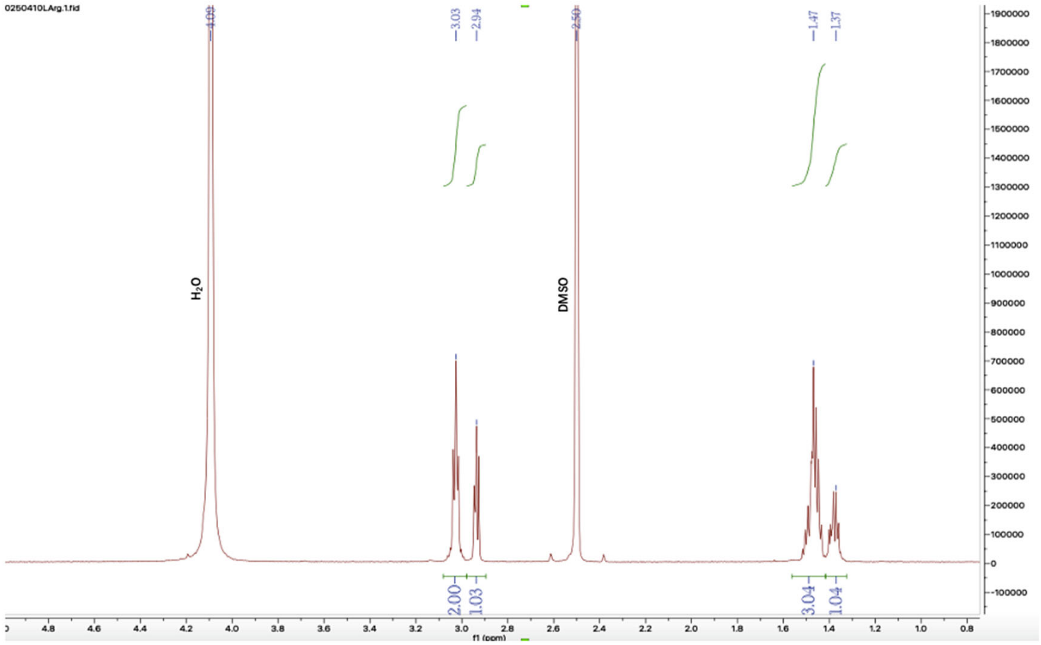

Figure S47. ^{1}H NMR of L-Arg in DMSO-d_6/14%D_2O. (600 MHz)

（4）^{1}H NMR and $^{13}C\{^{1}H\}$ NMR of $Zn(OAc)_2$ in DMSO-d_6/14%D_2O

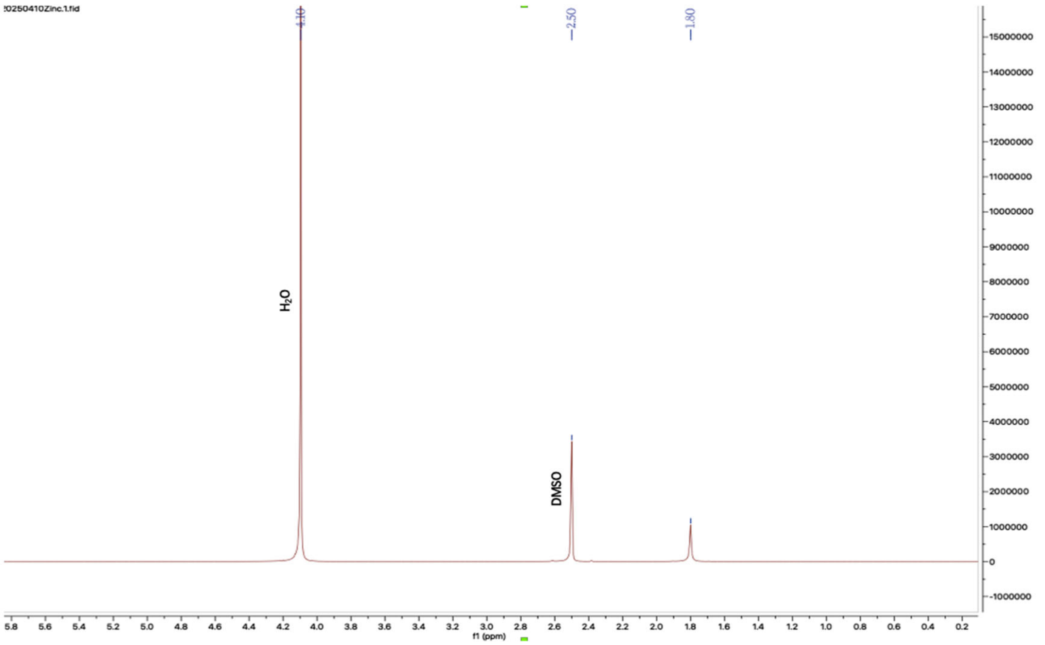

Figure S48. ^{1}H NMR of $Zn(OAc)_2$ in DMSO-d_6/14%D_2O. (600 MHz)

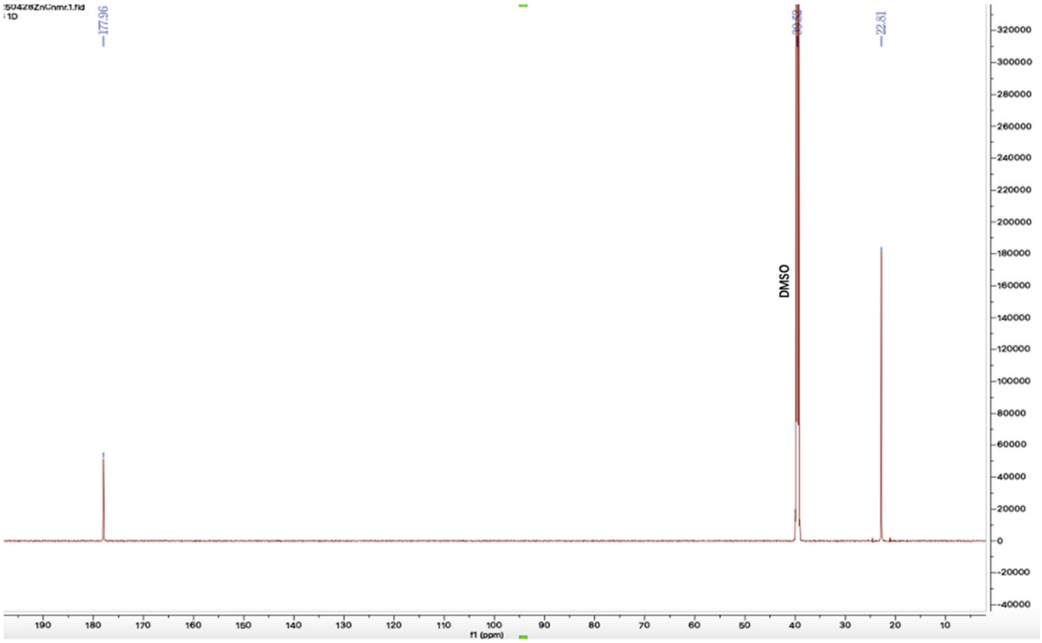

Figure S49. $^{13}C\{^{1}H\}$ NMR of $Zn(OAc)_2$ in DMSO-d_6/14%D_2O. (150 MHz)

4. NMR Spectroscopic Study on the Reaction of (*S,S*)-6 + Arginine in DMSO-d_6/17% D_2O with $Zn(OAc)_2$

(1) ^{1}H NMR spectra of (*S,S*)-**6** + 2 eq. D-Arg with the addition of various equivalents of $Zn(OAc)_2$

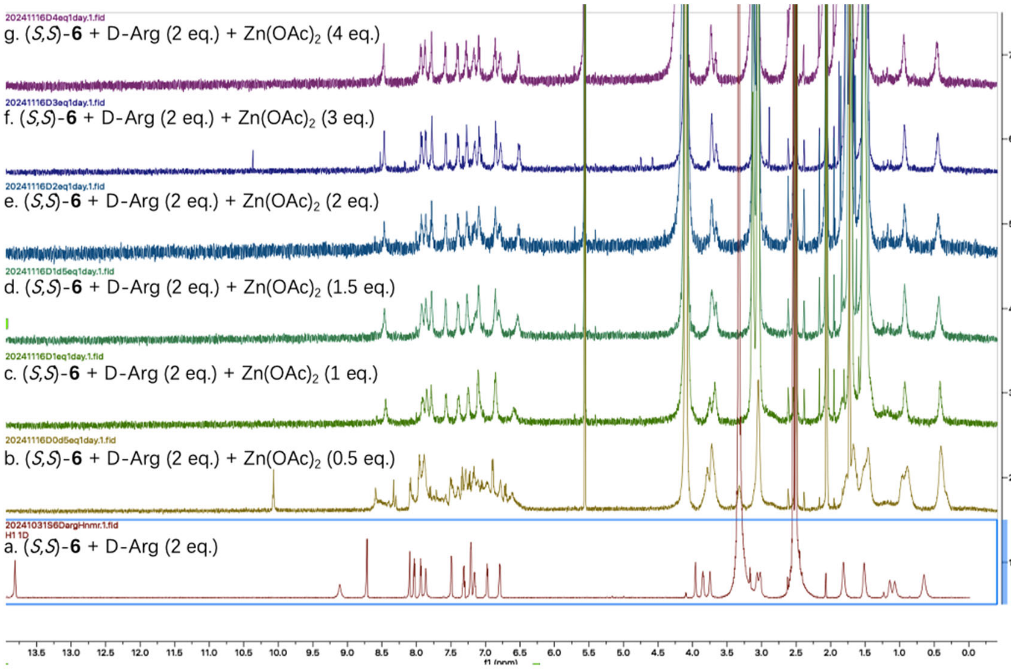

Figure S50. ^{1}H NMR spectra of (*S,S*)-**6** + 2 eq. D-Arg with the addition of various equivalents of $Zn(OAc)_2$ in DMSO-d_6/17%D_2O. (600 MHz)

(2) ^{1}H NMR spectra of (*S,S*)-**6** + 2 eq. L-Arg with addition of various equivalents of $Zn(OAc)_2$

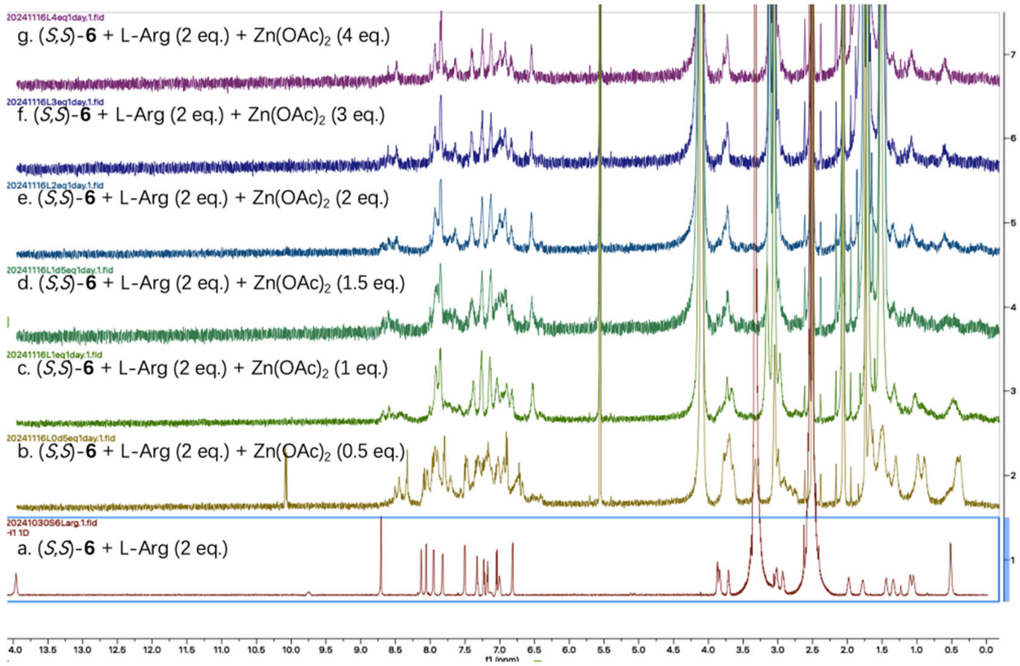

Figure S51. ^{1}H NMR of (*S,S*)-**6** + 2 eq. L-Arg with addition of various equivalents of $Zn(OAc)_2$ in DMSO-d_6/17%D_2O. (600 MHz)

5. Fluorescence Competitive Study

(1) Sample preparation procedure: competitive study procedure followed the general procedure by using L-Arg (3 equiv) + another amino acid enantiomers (1 equiv).

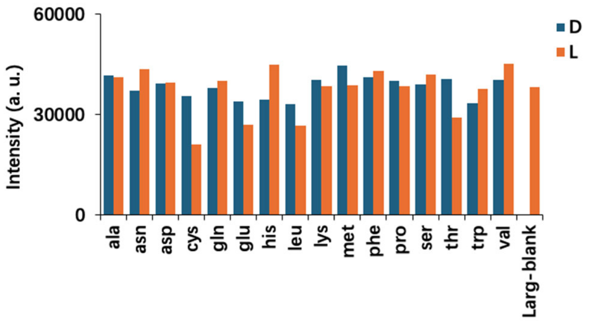

Figure S52. Fluorescence intensity of (*S,S*)-**6** (0.25 mM) + $Zn(OAc)_2$ (1.0 equiv) with L-Arg (3.0 equiv) + one of 16 common amino acids and their enantiomers (1.0 equiv) at 550 nm. (λ_{exc} = 405 nm. Slit: 5/5 nm. Solvent: DMSO / water = 1 / 1.5 with 12.5 mM pH 7.0 phosphate buffer)

(2) Sample preparation procedure: competitive study procedure followed the general procedure by using L-Arg (3 equiv) + another amino acid enantiomers (0.1 equiv).

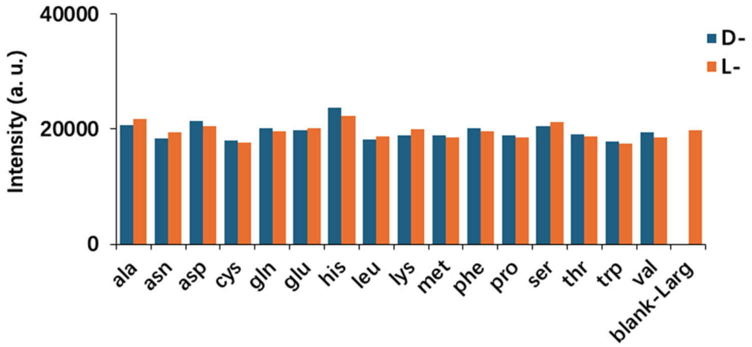

Figure S53. Fluorescence intensity of (*S,S*)-**6** (0.25 mM) + Zn(OAc)$_2$ (1.0 equiv) with L-Arg (3 equiv) + one of 16 common amino acids and their enantiomers (0.1 equiv) at 550 nm. (λ_{exc} = 405 nm. Slit: 5/5 nm. Solvent: DMSO / water = 1 / 1.5 with 12.5 mM pH 7.0 phosphate buffer)

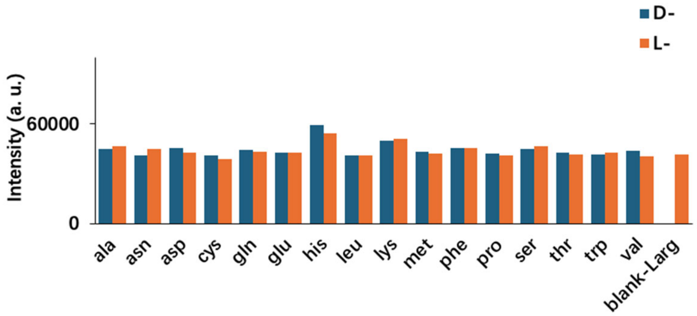

Figure S54. Fluorescence intensity of (*S,S*)-**6** (0.25 mM) + Zn(OAc)$_2$ (1.0 equiv) with L-Arg (3 equiv) + one of 16 common amino acids and their enantiomers (0.1 equiv) at 550 nm. (λ_{exc} = 440 nm. Slit: 5/5 nm. Solvent: DMSO / water = 1 / 1.5 with 12.5 mM pH 7.0 phosphate buffer)

6. Limit of Detection of L-Arg by (*S,S*)-6

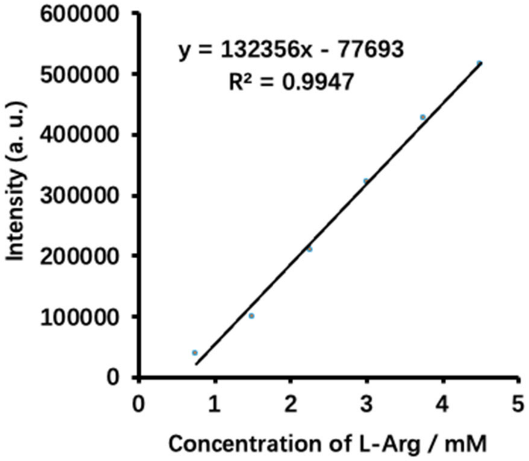

Figure S55. The fluorescence intensity at 550 nm of (*S,S*)-**6** (0.25 mM) + $Zn(OAc)_2$ (1.0 equiv) with varying concentration of L-Arginine. (Error bars from three independent experiments. λ_{exc} = 440 nm. Slit: 5/5 nm. Solvent: DMSO / water = 1 / 1.5 with 12.5 mM pH 7.0 buffer)

The Limit of Detection (LOD) is calculated by the following equation:

$$\text{LOD} = 3.3(\sigma/s) = 3.3(154.6634/132356) = 3.8562\ \mu\text{M}$$

Where $\sigma =$ the standard deviation of the blank responses (3 times)

$s =$ the slope of the titration curve.
